# Cytoplasmic contractile injection systems mediate cell death in *Streptomyces*

**DOI:** 10.1038/s41564-023-01341-x

**Published:** 2023-03-09

**Authors:** Bastien Casu, Joseph W. Sallmen, Susan Schlimpert, Martin Pilhofer

**Affiliations:** 1 Department of Biology, Institute of Molecular Biology and Biophysics, Eidgenössische Technische Hochschule Zürich, Zürich, Switzerland; 2 John Innes Centre, Department of Molecular Microbiology, Norwich Research Park, Norwich, UK

**Keywords:** Cellular microbiology, Cryoelectron tomography

## Abstract

Contractile injection systems (CIS) are bacteriophage tail-like structures that mediate bacterial cell–cell interactions. While CIS are highly abundant across diverse bacterial phyla, representative gene clusters in Gram-positive organisms remain poorly studied. Here we characterize a CIS in the Gram-positive multicellular model organism *Streptomyces coelicolor* and show that, in contrast to most other CIS, *S. coelicolor* CIS (CIS^Sc^) mediate cell death in response to stress and impact cellular development. CIS^Sc^ are expressed in the cytoplasm of vegetative hyphae and are not released into the medium. Our cryo-electron microscopy structure enabled the engineering of non-contractile and fluorescently tagged CIS^Sc^ assemblies. Cryo-electron tomography showed that CIS^Sc^ contraction is linked to reduced cellular integrity. Fluorescence light microscopy furthermore revealed that functional CIS^Sc^ mediate cell death upon encountering different types of stress. The absence of functional CIS^Sc^ had an impact on hyphal differentiation and secondary metabolite production. Finally, we identified three putative effector proteins, which when absent, phenocopied other CIS^Sc^ mutants. Our results provide new functional insights into CIS in Gram-positive organisms and a framework for studying novel intracellular roles, including regulated cell death and life-cycle progression in multicellular bacteria.

## Abstract (web summary)

Characterization of a contractile injection system (CIS) in the Gram-positive multicellular model organism *Streptomyces coelicolor* finds that CIS mediate cell death in response to stress that impacts cellular development.

## Main

Bacteria exist in highly competitive environments that require them to interact with a range of organisms. To respond to potential stressors, bacteria have evolved complex strategies to mediate potential antagonistic interactions^1^. One such response is the deployment of cell-puncturing nanodevices called contractile injection systems (CIS), which are large macromolecular protein machines that can translocate cytotoxic effectors into the extracellular space or directly into target cells^2–5^. In general, CIS are composed of a contractile sheath that surrounds an inner tube loaded with effectors, fitted with a baseplate complex. A conformational change in the baseplate complex triggers the contraction of the outer sheath, which leads to the propulsion of the inner tube into the target^6,7^.

Phylogenetic analyses have indicated that these CIS are conserved across diverse microbial phyla, including Gram-negative and Gram-positive bacteria, as well as archaea^8,9^. CIS are commonly classified as Type VI secretion systems (T6SS) or extracellular CIS (eCIS) on the basis of their mode of action. Anchored at the host’s cytoplasmic membrane, T6SSs function via a cell–cell contact-dependent mechanism wherein the T6SS injects effectors directly into a neighbouring cell^10–14^. In contrast, eCIS are assembled in the bacterial cytoplasm of the donor cell and are subsequently released into the extracellular space where they can bind to the surface of a target cell, contract and puncture the cell envelope^15–18^. Recently, a third mode of action was described in multicellular *Cyanobacteria*^19^. This system is also assembled in the bacterial cytoplasm and then attaches to the thylakoid membrane where it potentially induces lysis of the cell upon stress, resulting in the formation of ‘ghost cells’, which may in turn proceed to interact with other organisms^19^.

Of the hundreds of putative CIS gene clusters detected in bacteria, all well-characterized examples have come from two closely related clades and have been exclusively examined in Gram-negative bacteria. Characterized CIS representatives include ‘metamorphosis-associated contractile structures’ (MACs) from *Pseudoalteromonas luteoviolacea*^16^, the ‘T6SS subtype *iv*’ (T6SS^*iv*^) in *Candidatus* Amoebophilus asiaticus^7^, ‘antifeeding prophages’ (AFPs) from *Serratia*^17^, ‘*Photorhabdus* virulence cassettes’ (PVCs) from *P. asymbiotica*^18^ and two newly characterized CIS from the marine bacteria *Algoriphagus machipongonensis*^15^ and cyanobacteria^19^.

Strikingly, 94 of 116 sequenced Gram-positive actinomycetes of the genus *Streptomyces* were shown to encode a potential CIS gene cluster^8,9^. A previous report suggested that CIS from *Streptomyces lividans* were involved in microbial competition; however, the mechanism remains unknown^20^. *Streptomyces* species are multicellular soil bacteria, renowned for their complex developmental life cycle and their ability to produce an array of clinically relevant secondary metabolites^21^. The *Streptomyces* life cycle begins with the germination of a spore and generation of germ tubes, which grow by apical tip extension and hyphal branching to form a dense vegetative mycelium. Upon nutrient depletion, non-branching aerial hyphae are erected, which synchronously divide into chains of uni-nucleoid spores^22^. Notably, the production of important secondary metabolites is tightly coordinated with the developmental life cycle^21^.

Here we provide evidence that CIS from the model organism *Streptomyces coelicolor* (CIS^Sc^) function intracellularly and belong to a new class of CIS that exist as free-floating fully assembled particles in the cytoplasm and mediate cell death in response to stress conditions. Additionally, we find that the absence of CIS affects the coordinated cellular development and secondary metabolite production of *S. coelicolor*, indicating a wider role of CIS from *Streptomyces* in the multicellular biology of these important bacteria. Our data are consistent with a recent paper^23^.

## Results

### *Streptomyces* express cytoplasmic CIS in vegetative growth

**Fig. 1:**
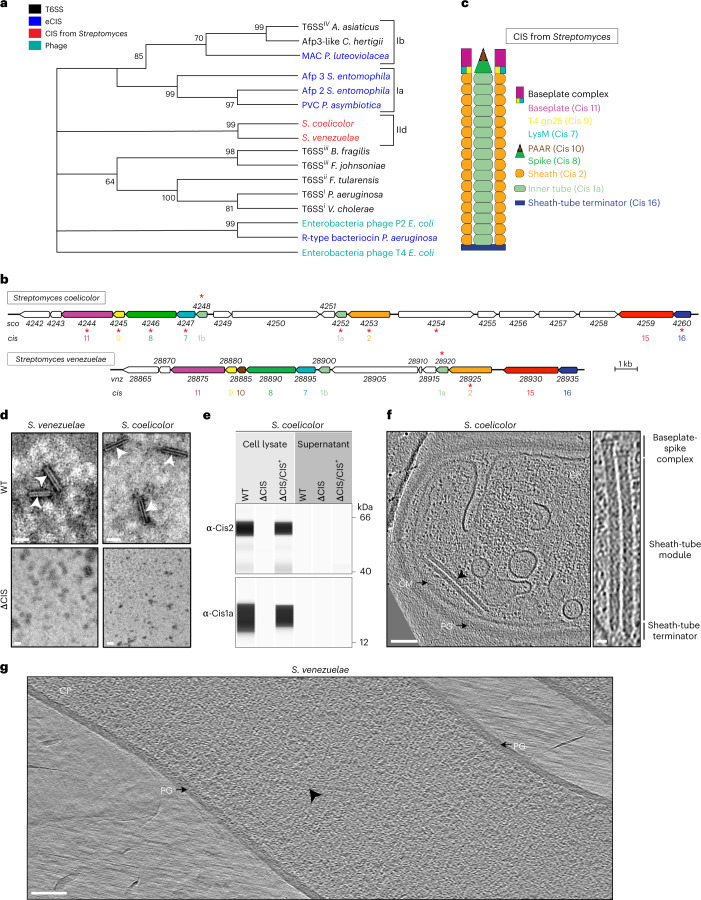
Different *Streptomyces* species express cytoplasmic CIS assemblies. **a**, Phylogenetic analysis of representative sheath protein sequences shows that homologues from *Streptomyces* form a monophyletic clade. Numbers indicate bootstrap values, colour code denotes different modes of action. Subclades Ia, Ib and IId are based on the dbeCIS database^8^. **b**, Representative gene clusters from *Streptomyces* encode conserved CIS components. The schematic shows the gene arrangement of the CIS gene clusters from *S. coelicolor A3(2)* (CIS^Sc^) and *S. venezuelae NRRL B-65442* with gene locus tags. Colour code indicates conserved gene products. CIS components were numbered on the basis of similarities to previously studied CIS (AFP)^19,67^. Asterisks indicate gene products that were detected by mass spectrometry after CIS purification (Supplementary Table 1). **c**, The schematic illustrates a putative CIS assembly from *Streptomyces*. Colour code is based on the predicted gene function shown in **b**. **d**, *cis2* is required for CIS assembly. Shown are negative-stain EM images of crude sheath preparations from WT and ΔCIS mutant strains of *S. coelicolor* and *S. venezuelae*. White arrowheads indicate contracted sheath-like structures. Shown are representative micrographs of three independent preparations. Scale bars, 80 nm. **e**, CIS^Sc^ proteins are detected in the cell lysate but not secreted into the supernatant. Shown is the automated western blot analysis of cultures of WT *S. coelicolor*, ΔCIS mutant and a complementation (ΔCIS/CIS^+^). The presence of the sheath protein (Cis2) and the inner tube protein (Cis1a) in whole-cell lysates and concentrated culture supernatants was probed using polyclonal antibodies against Cis1a/2. Experiments were performed in biological triplicates. For the control sodium dodecyl sulfate–polyacrylamide gel electrophoresis (SDS–PAGE) gel, see Extended Data Fig. 1c. **f**, Cryo-electron tomogram of a WT *S. coelicolor* hypha revealing two cytoplasmic extended CIS^Sc^ assemblies (arrowhead). Eighty tomograms were collected from 10 independent datasets. PG, peptidoglycan; CM, cytoplasmic membrane; CP, cytoplasm. Putative structural components are indicated on the right. Scale bars, 75 nm and 12.5 nm (magnified inset). **g**, Tomogram of a cryoFIB-milled WT *S. venezuelae* hypha revealing one cytoplasmic extended CIS^Sv^ assembly (arrowhead). Free-floating extended CIS^Sv^ were observed in 12 tomograms from 3 independent datasets. Scale bar, 75 nm.

Previous bioinformatic studies revealed that the majority of sequenced *Streptomyces* genomes harbour a highly conserved cluster of eCIS genes related to the poorly studied CIS IId subtype^8,9^. This was further confirmed by our phylogenetic analyses using sheath protein sequences from known producers of CIS and from two representative *Streptomyces* species, namely *S. coelicolor* and *Streptomyces venezuelae* (Fig. 1a).

Closer inspection of the *Streptomyces* CIS gene clusters from *S. coelicolor* (*sco4242–sco4260*) and *S. venezuelae* (*vnz_28865–vnz_28935*) suggested that both species encode 10 or 11 core structural components of the phage tail-like systems, respectively^8,9^ (Fig. 1b,c). On the basis of this sequence similarity, we renamed the genes from *Streptomyces* to *cis1–16*. Both CIS gene clusters encode two inner tube homologues (*cis1a* and *cis1b*), as well as additional proteins of unknown function. Cis10, a PAAR-repeat containing protein, is only present in *S. venezuelae*. Absent from both gene clusters are the following factors: a gene encoding a conventional tail fibre protein (Afp13)^24^, which mediates eCIS binding to target cells; a typical tape measure protein (Afp14)^25^, which is involved in regulating eCIS length^17^; and a ClpV homologue, which is implicated in recycling some T6SSs^26^.

**Extended Data Fig. 1:**
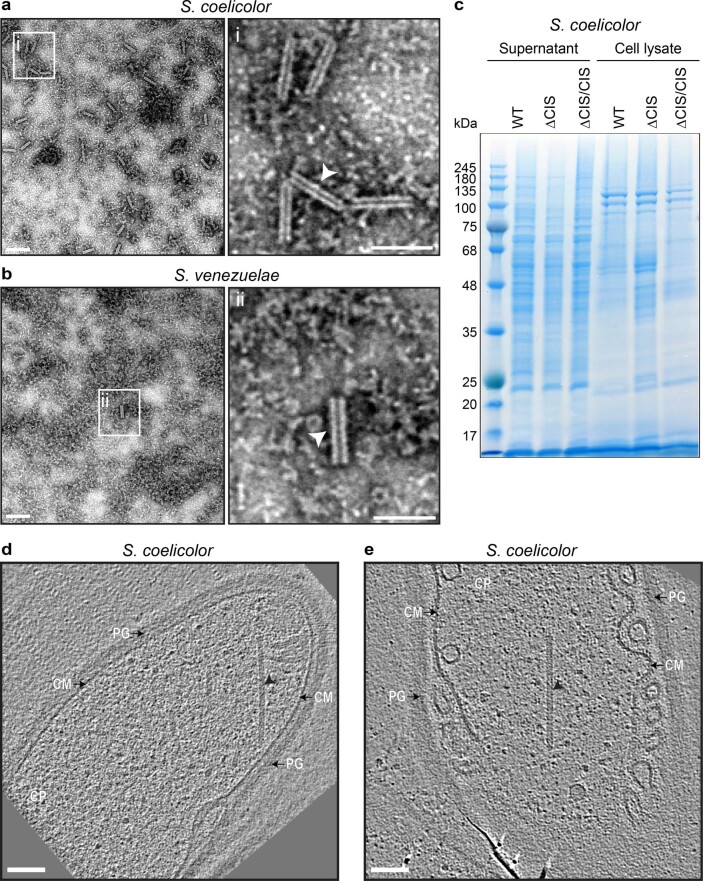
EM and SDS-PAGE analyses of *Streptomyces* supernatant and lysate and additional tomograms that demonstrate localization of CIS in the cytoplasm. **a/b**. Representative negative−stain electron micrographs of crude sheath preparations from WT *S. coelicolor* and *S. venezuelae*. Under the conditions used, the majority of isolated CIS from *Streptomyces* was contracted (insets i/ii). These experiments were performed three independent times. Bars, 80 nm. **c**. Loading control SDS-PAGE stained with Coomassie−blue confirming the presence of protein in the same samples that were used for the detection of Cis1a/2 by automated Western blot analysis in Fig. 1e. Samples were obtained from WT *S. coelicolor*, the ΔCIS mutant and the complemented mutant ΔCIS/CIS^+^. 10 μg of protein was loaded per sample. This experiment was performed in biological triplicates. **d/e**. Shown are cryo-electron tomograms of a WT *S. coelicolor* hyphae, revealing cytoplasmic extended CIS^Sc^ assemblies (arrowhead). 80 tomograms were collected from 10 independent datasets. PG, peptidoglycan; CM, cytoplasmic membrane; CP, cytoplasm. Bars, 85 nm.

To test whether *S. coelicolor* and *S. venezuelae* produced CIS, we purified sheath particles from crude cell lysates, followed by negative-stain electron microscopy (EM) imaging. We observed typical contracted sheath-like particles in crude extracts from wild-type (WT) *S. coelicolor* and *S. venezuelae*; no such assemblies were seen in strains carrying a deletion in the gene that encodes the sheath Cis2 (ΔCIS, Fig. 1d). Mass spectrometry analysis of the purified WT particles detected peptides from Cis1a (inner tube) and Cis2 (sheath) (Supplementary Table 1), confirming that the CIS gene clusters from *Streptomyces* encode CIS-like complexes. We noticed that *S. coelicolor* produced approximately 50 times more sheath particles compared with *S. venezuelae* (Extended Data Fig. 1a,b). Therefore, we focused on the characterization of CIS from *S. coelicolor* (CIS^Sc^) in subsequent experiments.

To test whether CIS^Sc^ displayed a mode of action similar to canonical eCIS, we investigated whether CIS^Sc^ were released from cells into the extracellular space. Using automated western blotting, we analysed the culture supernatant and whole-cell extracts from WT and ΔCIS *S. coelicolor* cells that were grown for 48 h in liquid medium. Interestingly, we detected the two key CIS^Sc^ components Cis1a (inner tube) and Cis2 (sheath) only in whole-cell lysates but not in the supernatant of cultures of the WT or the complemented ΔCIS mutant (Fig. 1e and Extended Data Fig. 1c). These findings suggest that the entire CIS^Sc^ assembly is retained in the cytoplasm, unlike typical T6SS (inner tube protein translocated into the medium) and unlike eCIS (full assemblies released into the medium)^10,15^. Next, to visualize the localization of CIS^Sc^ in situ, we imaged hyphae of *S. coelicolor* and *S. venezuelae* by cryo-electron tomography (cryoET). While intact *S. coelicolor* hyphae could be imaged directly, *S. venezuelae* hyphae were too thick and had to be thinned by cryo-focused ion beam (FIB) milling before imaging. We predominantly found extended CIS that appeared to be free-floating in the cytoplasm, a behaviour that is inconsistent with a T6SS mode of action (Fig. 1f,g and Extended Data Fig. 1d,e). Taken together, these results indicate that CIS from *Streptomyces* may play a role in intracellular processes, which would be distinct from the previously described functions for T6SS and eCIS.

### Structure, engineering and subcellular localization of CIS^Sc^

**Fig. 2:**
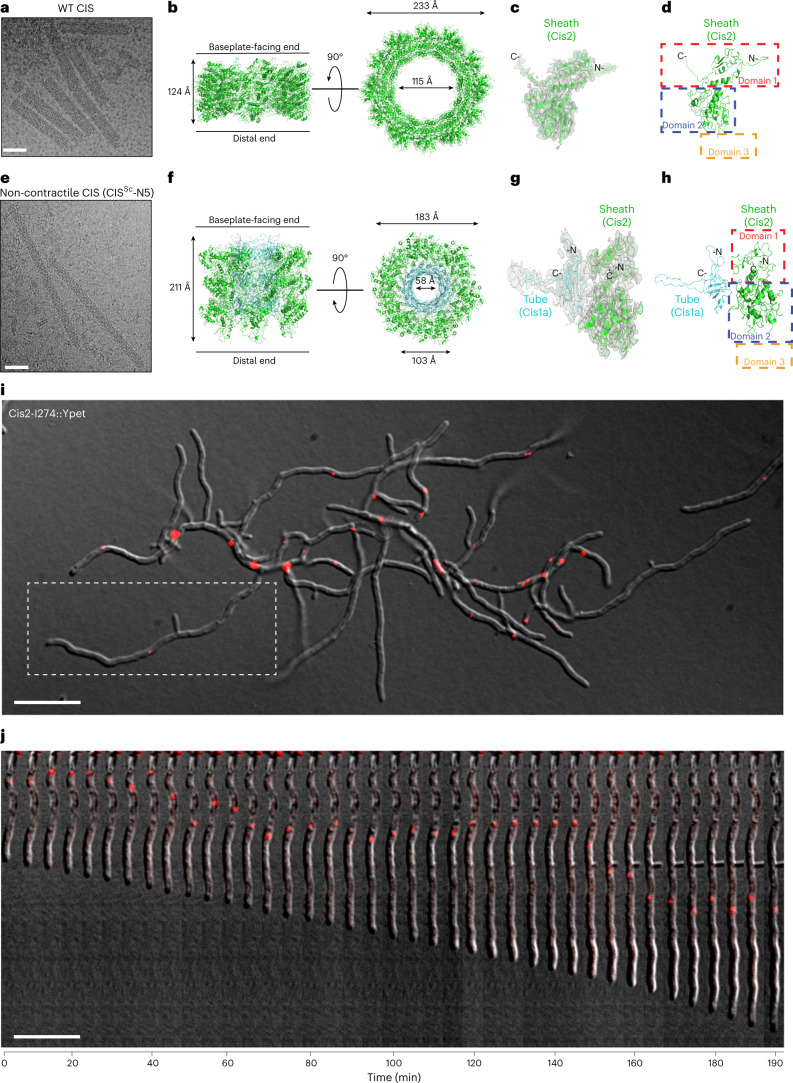
Structure and subcellular localization of CIS^Sc^. **a**, Representative cryo-electron micrograph of a sheath preparation from WT *S. coelicolor* that was recorded for structure determination (505 micrographs were collected). All sheath structures were seen in the contracted state. Scale bar, 40 nm. **b**, Section of the CIS^Sc^ sheath cryoEM structure in the contracted conformation. Left: side view. Right: top view. **c**,**d**, Side view ribbon representation of the Cis2 monomer in its contracted state superposed with the corresponding cryoEM map (**c**) and with dashed rectangles highlighting the positions of domains 1 (red), 2 (blue) and 3 (orange, not resolved because of high flexibility) (**d**). **e**, Representative cryo-electron micrograph of a sheath preparation from *S. coelicolor* expressing a non-contractile mutant of Cis2 (CIS-N5) (525 micrographs were collected). More than 95% of all structures were seen in the extended state. Scale bar, 40 nm. **f**, Ribbon representation of a section of the *S. coelicolor* Cis2 (sheath)-Cis1a (inner tube) cryoEM structure in the extended conformation that was solved using the non-contractile mutant. Left: side view. Right: top view. **g**,**h**, Side view ribbon representation of the Cis1a-Cis2 monomer (non-contractile mutant) in its extended state superposed with the corresponding cryoEM map (**g**) and with dashed rectangles highlighting the positions of domains 1 (red), 2 (blue) and 3 (orange, not resolved because of high flexibility) (**h**). **i**, Insights from the cryoEM structures enabled us to tag Cis2 with a fluorescent tag (YPet) for subsequent time-lapse imaging to determine the localization of assembled CIS^Sc^. Shown is a still image from Supplementary Video 1 showing scattered fluorescent foci inside vegetative hyphae. White rectangle highlights hypha shown in **h**. Scale bar, 10 µm. **j**, Fluorescently tagged CIS^Sc^ remained largely static or showed short-range movements over time. Shown is an image montage of a representative growing *S. coelicolor* hypha from Supplementary Video 1. Note that the depicted hypha has been rotated. Images were acquired every 5 min. Scale bar, 10 µm.

**Extended Data Fig. 2:**
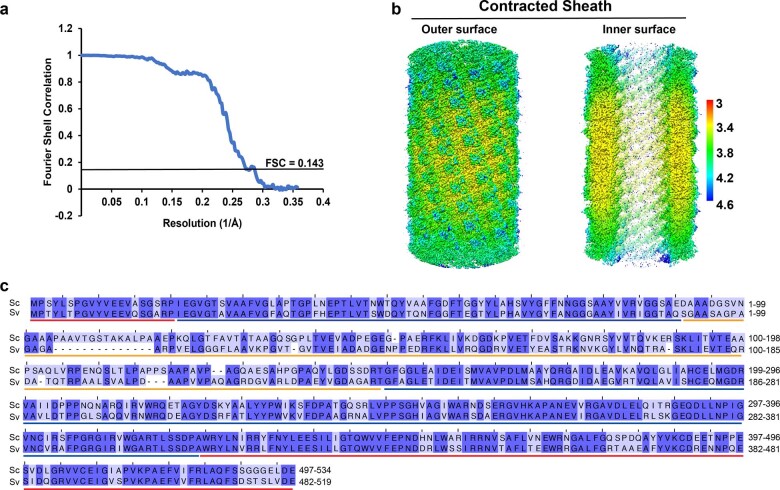
Structure and sequence analysis of *Streptomyces* contracted sheath Cis2. **a/b**. Gold-standard Fourier shell correlation (FSC) curve (a) and local resolution maps (b) of the contracted sheath (Cis2) structures from *S. coelicolor*. Scale in Å resolution. **c**. Protein sequence alignment showing the high sequence conservation between Cis2 proteins from *S. coelicolor* (Sc) and *S. venezuelae* (Sv). Colors indicate level of sequence similarity (light blue, similar; dark blue, identical). Positions of domain 1 (red), domain 2 (blue) and domain 3 (orange) are indicated.

To obtain insights into the structural details of the CIS^Sc^ contractile sheath-tube module, we performed single-particle cryoEM (helical reconstruction) of purified sheath particles from WT *S. coelicolor*, which had a homogeneous length for the contracted sheath of ~140 nm (Fig. 2a,b and Supplementary Table 2). The resulting map reached a resolution of 3.6 Å (Fig. 2c and Extended Data Fig. 2a,b). Contracted sheath proteins adopt a right-handed helical array with an inner diameter of 115 Å and an outer diameter of 233 Å (Fig. 2b). Similar to the recently described sheath structures observed in AlgoCIS^15^ and tCIS^19^, the CIS^Sc^ sheath is composed of a single protein, Cis2. Cis2 monomers consist of three domains and are well conserved in *S. coelicolor* and *S. venezuelae*, sharing ~65% sequence identity (Extended Data Fig. 2c). From the resulting map, it was possible to build de novo domains 1 and 2, which contribute to the sheath wall (Fig. 2d). The additional domain 3, which is located on the sheath surface, seems to be highly flexible and could not be resolved. The overall contracted structure of Cis2 is similar to sheaths of previously characterized systems^18,27,28^.

**Extended Data Fig. 3:**
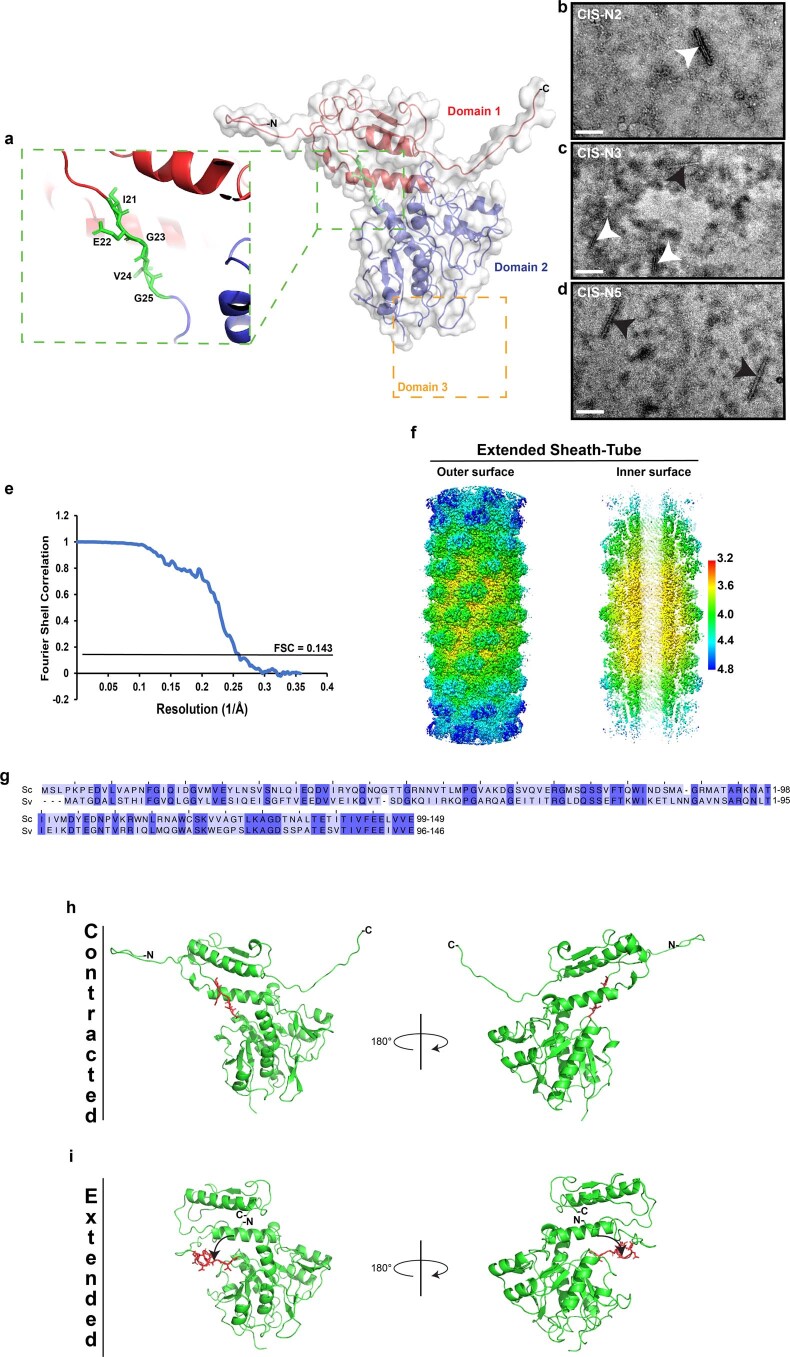
Engineering and structure of a non-contractile CIS^Sc^ mutant. **a**. Surface (grey) and ribbon (colored) representation of the contracted sheath structure from *S. coelicolor*. Shown in the enlarged inset is the WT linker comprising residues I21 to G25 (green). To engineer non-contractile CIS^Sc^ mutants, additional residues were inserted after position G25 (IE for CIS*-*N2, IEG for CIS*-*N3, IEGVG for CIS*-*N5). Dashed orange rectangle highlights the domain 3 (not resolved because of high flexibility). **b-d**. Negative-stain electron micrographs of CIS particles from *S. coelicolor* strains expressing the CIS^Sc^ mutant versions CIS*-*N2, CIS-N3 and CIS-N5. Sheath mutants carrying the CIS-N5 allele showed the highest fraction of extended structures. Arrowheads indicate contracted (white) and extended (black) CIS^Sc^ particles. These experiments were performed three independent times. Bar, 140 nm. **e, f**. Gold-standard Fourier shell correlation (FSC) curve (e) and local resolution maps (f) of the extended *S. coelicolor* CIS^Sc^-N5 sheath-tube module. Scale in Å resolution. **g**. Protein sequence alignment showing the high conservation of Cis1a proteins from *S. coelicolor* (Sc) and *S. venezuelae* (Sv). Colors indicate level of sequence similarity (light blue, similar; dark blue, identical). h/i. Ribbon representation of the contracted (h) and extended (i) sheath structures from *S. coelicolor*. Shown in red is the potential position of the flexible linker between the domain 1 and domain 2 of the sheath protein. Black arrows indicate the movement of this linker.

To be able to purify the extended form of the CIS^Sc^ sheath-tube module from *S. coelicolor* cell lysates, we engineered non-contractile CIS^Sc^, on the basis of the information from the contracted Cis2 structure and similar approaches used previously for *Vibrio cholerae*^29^ and enteroaggregative *Escherichia coli*^30^ (Extended Data Fig. 3a). Different sets of two (IE), three (IEG) and five (IEGVG) amino acid residues were inserted into the N-terminal linker of Cis2 after position G25, resulting in the mutants CIS-N2, CIS-N3 and CIS-N5, respectively. For the CIS-N2 and CIS-N3 mutants, less than 30% and 50% were found in extended form, respectively (Extended Data Fig. 3b,c). For the CIS-N5 non-contractile mutant, more than 95% of the complexes were seen in the extended conformation (Extended Data Fig. 3d). In vitro, the length of the CIS-N5 non-contractile mutant was homogeneous at ~230 nm (Fig. 2e). Moreover, mass spectrometry analyses confirmed the presence of most CIS^Sc^ components, indicating the stability of the complex (Fig. 1b and Supplementary Table 1).

Next, we optimized the purification of CIS-N5 particles and performed cryo-EM. Helical reconstruction was used to generate an EM map, which we then used to build de novo the sheath-tube (Cis2-Cis1a) module in the extended conformation at 3.9 Å resolution (Fig. 2f–h, Extended Data Fig. 3e,f and Supplementary Table 2). Domain 3 of the extended sheath (Cis2) was again too flexible to be resolved. The tube (Cis1a) structure and fold are highly similar to the tube structures already described for other CIS (Fig. 2h and Extended Data Fig. 3g). The comparison of the sheath (domains 1/2) in the extended versus contracted states revealed an increase in diameter and shortening of the length upon contraction, similar to other CIS (Fig. 2b,f)^15,18,19,28,31^. The introduction of five extra amino acids in the CIS-N5 mutant induced conformational changes at the Cis2 N terminus, similar to a previous report (Extended Data Fig. 3h,i)^31^.

**Extended Data Fig. 4:**
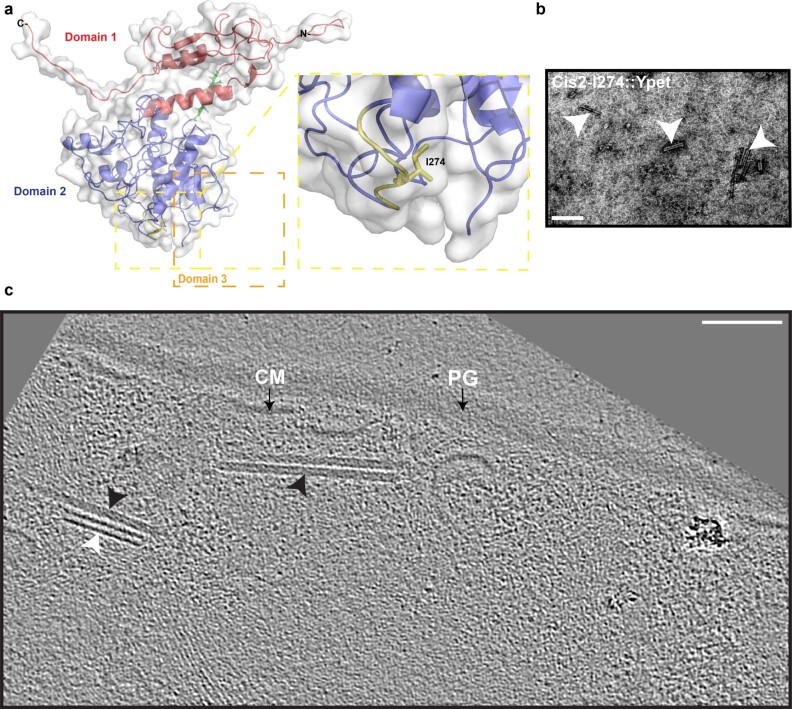
Generation of a functional Cis2-YPet sandwich fusion. **a**. Surface and ribbon diagram of the contracted sheath structure from *S. coelicolor* indicating the insertion site of Ypet at residue I274 to generate a fluorescent sheath-Ypet sandwich fusion (Cis2::I274-Ypet), which was used to complement a *S. coelicolor* ΔCis2 mutant. Dashed yellow rectangles highlight the position used for the insertion of the Ypet sequence (yellow). Note that domain 3 could not be resolved because of its high structural flexibility. **b**. Negative-stain electron micrograph of purified CIS particles from *S. coelicolor ∆cis2/cis2::I274-ypet*^*+*^ showing contracted CIS^Sc^ assemblies (white arrowheads). This experiment was performed in three biological replicates. Bar, 140 nm. **c**. Representative cryoET slice of *∆cis2/cis2::I274-ypet*^*+*^ hyphae containing a contracted (white arrowhead) and extended (black arrowhead) CIS^Sc^ particles in the cytoplasm. 50 tomograms were collected from 2 independent datasets. PG, peptidoglycan; CM, cytoplasmic membrane. Bar, 75 nm.

Guided by the high-resolution structure of the sheath module, we engineered a fluorescently tagged CIS^Sc^ by inserting YPet at position I274 in the Cis2 monomer. Subsequently, we used this Cis2-YPet sandwich fusion to complement the *S. coelicolor* Δ*cis2* mutant *in trans* (Extended Data Fig. 4a). Using negative-stain EM and cryoET, we confirmed that YPet-tagged CIS^Sc^ (CIS^Sc^-YPet) were able to assemble into extended particles and to contract, suggesting that these fluorescently labelled CIS^Sc^ particles were functional (Extended Data Fig. 4b,c). This construct enabled us to visualize the subcellular localization of CIS^Sc^ in vegetative hyphae using time-lapse fluorescence light microscopy (fLM). Multiple CIS^Sc^-YPet foci were found inside the hyphae but not in extracellular space. The foci were largely static or displayed short-range movements within the hyphae (Fig. 2i,g and Supplementary Video 1). CIS^Sc^-YPet foci were stable over the course of the experiment and did not reveal notable changes in the shape or intensity of the fluorescence. While this invariability indicates the absence of firing events during the experiment, the resolution in fLM and the relatively short length of the CIS^Sc^ may hamper the detection of firing events (in contrast to the much longer T6SSs^10,30^).

Taken together, our structural data allowed us to engineer non-contractile and fluorescently tagged CIS^Sc^, which revealed the presence of scattered CIS^Sc^ in *S. coelicolor* hyphae.

### CIS contraction correlates with state of cellular integrity

**Fig. 3:**
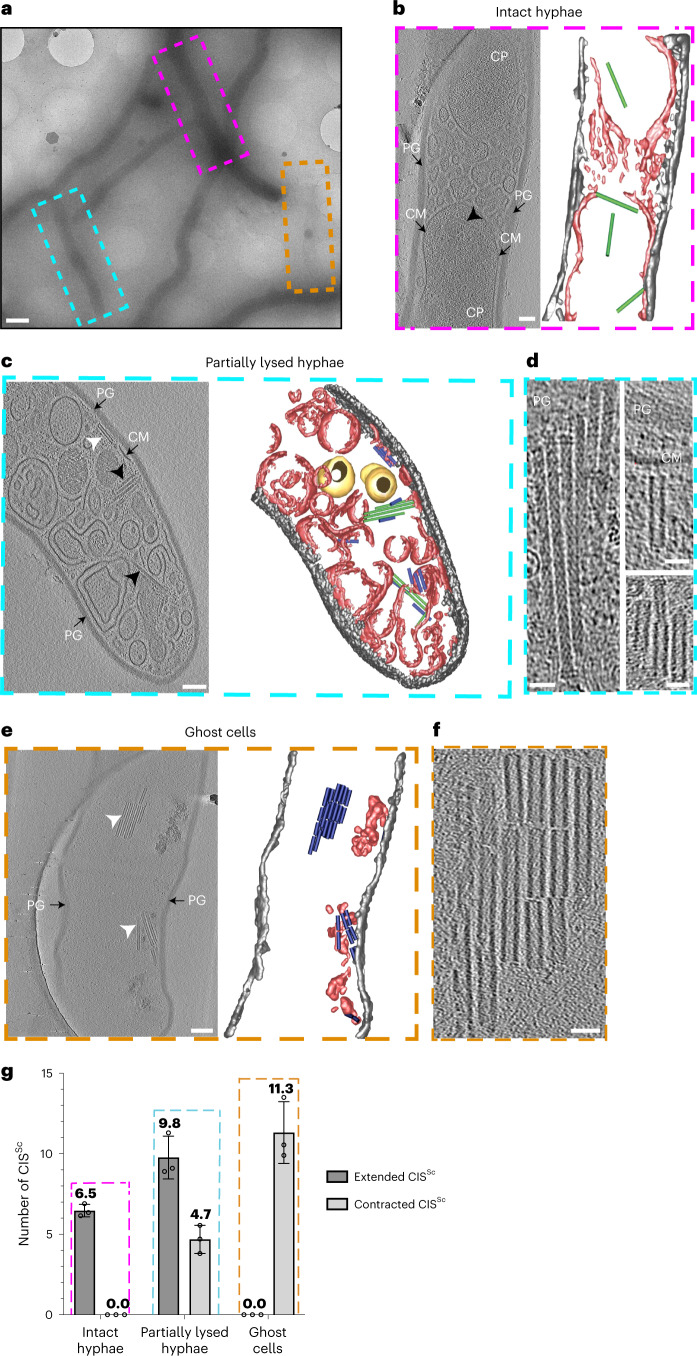
Sheath contraction is linked to reduced cellular integrity. **a**, Representative low-magnification 2D cryoEM image of WT *S. coelicolor* hyphae during vegetative growth. Hyphae were divided into three classes on the basis of their density in such images and their structure in cryo-tomograms: (1) ‘intact hyphae’ (purple box), (2) ‘partially lysed hyphae’ (cyan box) and (3) ‘ghost cells’ (orange box). Scale bar, 1 μm. **b**–**f**, Representative cryo-tomogram slices and 3D renderings of hyphae of the three classes (corresponding to the regions boxed in **a**). ‘Intact hyphae’ (**b**) had mostly intact cytoplasmic membranes and occasional vesicular membranous assemblies that are reminiscent of ‘cross-membranes’^32^. ‘Partially lysed hyphae’ (**c**) showed a mostly disrupted/vesiculated cytoplasmic membrane. ‘Ghost cells’ (**e**) contained only remnants of membranes and a mostly intact peptidoglycan cell wall. Note the frequent occurrence of CIS^Sc^ assemblies in extended (black arrowheads/green) and contracted (white arrowheads/blue) conformations. Magnified views of clusters of CIS^Sc^ seen in cryo-tomograms are shown in **d** and **f**. PG/grey, peptidoglycan; CM/red, cytoplasmic membrane/membranes; CP, cytoplasm; yellow, storage granules. Scale bars, 75 nm in **b**, **c** and **e**; 25 nm in **d** and **f**. **g**, Sheath contraction correlates with cellular integrity, showing the presence of only extended CIS^Sc^ in the class ‘intact hyphae’ and the presence of only contracted CIS^Sc^ in ‘ghost cells’. Shown is a quantification of extended and contracted CIS^Sc^ per tomogram of WT *S. coelicolor* hyphae. Data show mean ± s.d. obtained from biological triplicate experiments, with *n* = 30 tomograms for each class of cells.

Our initial cryoET data of *S. coelicolor* cells indicated that contracted CIS^Sc^ were frequently found in hyphae that displayed a damaged cell membrane. To explore this correlation further, we first acquired low-magnification two-dimensional (2D) cryoEM images. On the basis of the contrast of individual hyphae in these 2D images (Fig. 3a), we classified the hyphae into three distinct groups: (1) ‘intact hyphae’ (dark appearance in 2D) with mostly intact cytoplasmic membrane and occasional vesicular membranous assemblies that are reminiscent of ‘cross-membranes’^32^ (Fig. 3b); (2) ‘partially lysed hyphae’ with a mostly disrupted/vesiculated cytoplasmic membrane (reduced contrast in 2D), indicative of cytoplasmic leakage (Fig. 3c); and (3) membrane-less ‘ghost cells’ (hardly visible in 2D), lysed hyphae that only consisted of the peptidoglycan cell wall (Fig. 3e). Representative hyphae of each group (*n* = 90) were imaged by cryoET (270 tomograms in total, *n* = 3 experiments) and the conformational state and in situ localization of the CIS^Sc^ were determined (Fig. 3b–g). In addition, we performed 3D volume segmentation of selected full tomograms.

As we observed previously for intact hyphae (Fig. 1f), individual CIS^Sc^ particles were always found in the extended conformation and localized in the cytoplasm (Fig. 3b). In contrast, in partially lysed hyphae (Fig. 3c), the ratio of extended to contracted CIS^Sc^ was 2:1. CIS^Sc^ particles often appeared to cluster in the vicinity of membranous structures (Fig. 3d). Notably, we found that in some cases, the extended CIS^Sc^ aligned perpendicular to membrane patches or vesicles with the baseplate complex facing the membrane, suggesting that CIS^Sc^ may interact with the cytoplasmic membrane (Fig. 3c,d). In contrast, ghost cells only displayed CIS^Sc^ particles in the contracted state, which were often clustered together (Fig. 3f).

Collectively, these results indicate that the conformational state of CIS^Sc^ correlates with the integrity of the cell and that CIS^Sc^ may play an intracellular role as a consequence of an unknown cellular signal and lead to cell death, either directly or indirectly. Consequently, we hypothesized that such a signal may be triggered by exogenous stress and could result in the recruitment of CIS^Sc^ to the membrane and trigger firing.

### CIS contraction mediates cell death under stress conditions

**Extended Data Fig. 5:**
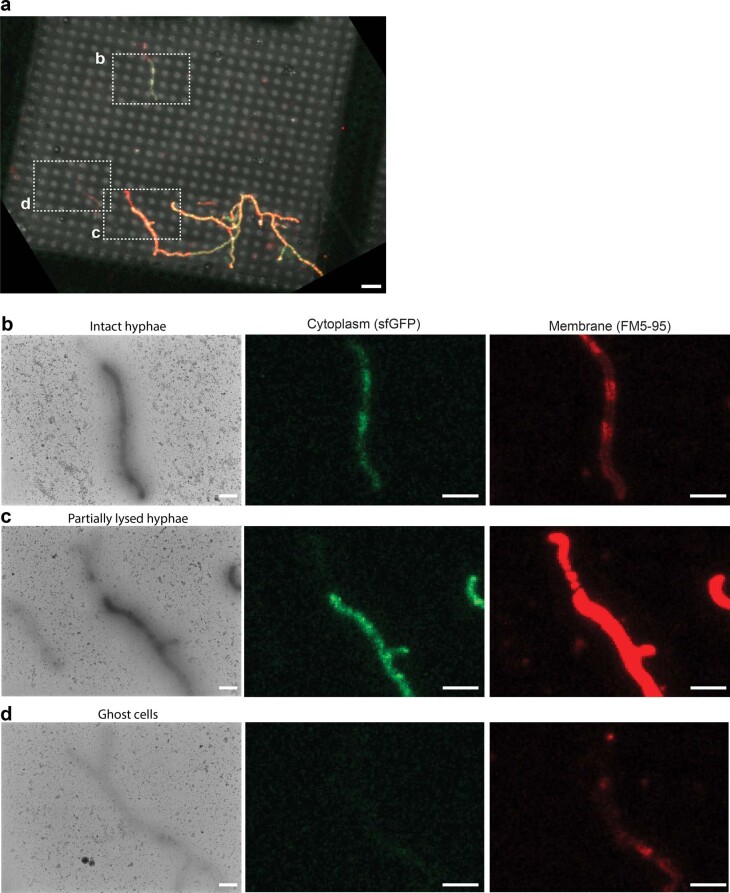
Validation of hyphal membrane integrity using correlative cryo-fLM and cryoEM (Cryo-CLEM). **a**. CryofLM overview image of vegetative hyphae of WT *S. coelicolor* expressing cytoplasmic sfGFP from a constitutive promoter and stained with the membrane dye FM5-95. The membrane staining pattern and sfGFP fluorescence signal were used to identify the classes ‘intact hyphae’, ‘partially lysed hyphae’ and ‘ghost cells’. The boxed areas were further analyzed in (b-d). Bar, 6 µm. **b-d**. Shown are cryoEM 2D projection images (left) and the corresponding cryo-fLM images of examples of ‘intact hyphae’ (b), ‘partially lysed hyphae’ (c) and ‘ghost cells’ (d). Bars, 2 µm.

To test this hypothesis, we explored whether upon encountering stress, the presence of CIS^Sc^ and their contraction could mediate cell death. To generate a marker for cell viability, we inserted *sfgfp* under the control of a constitutive promoter *in trans* in WT *S. coelicolor*, in the null mutant (ΔCIS) and in the non-contractile mutant (CIS-N5). To label intact and partially lysed hyphae, cells were incubated with the fluorescent membrane dye FM5-95. We first used correlated cryo-light and electron microscopy (CLEM) to confirm that the detected cytoplasmic and membrane fluorescence correlated with the physiological state of the hyphae (Extended Data Fig. 5). To assess the level of cell death in the imaged strains, we used fLM and quantified the ratio of the sfGFP signal (indicator of viable hyphae) to the FM5-95 signal (indicator of intact and lysed hyphae) in the different strains. Cells were grown for 48 h in liquid, a timepoint at which CIS^Sc^ can be detected in hyphae (Fig. 1d,e).

**Fig. 4:**
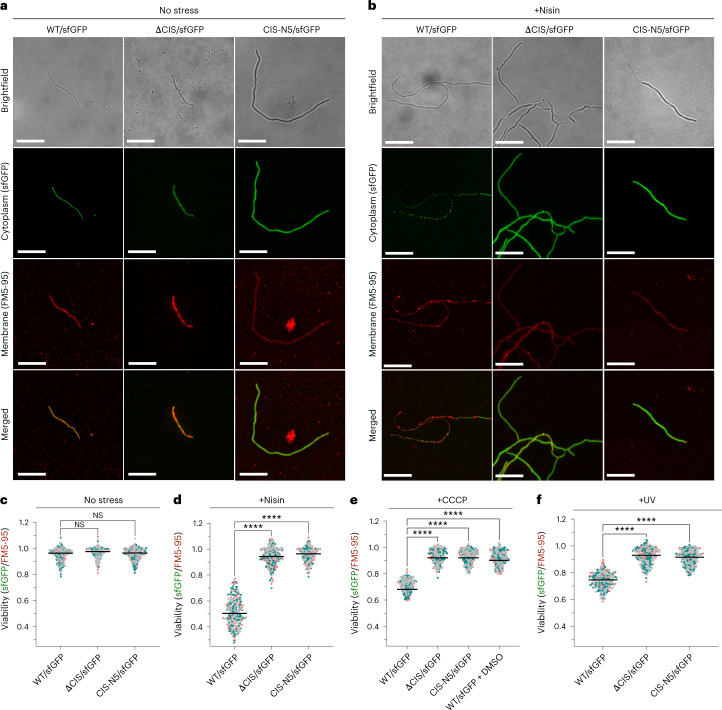
*S. coelicolor* with functional CIS^Sc^ show increased cell death upon stress. **a**,**b**, fLM (shown are representative images) was used to determine the ratio between live cells (cytoplasmic sfGFP) and total cells (membrane dye FM5-95) after growth in the absence of stress (**a**) or in the presence of nisin stress (**b**). *S. coelicolor* WT/sfGFP, ∆CIS/sfGFP and CIS-N5/sfGFP were grown in TSB medium for 48 h and then treated with 1 µg ml^−1^ nisin for 90 min. Scale bars, 10 µm. **c**,**d**, Quantification of the experiments in **a** and **b** showed no notable differences between the WT strain and both CIS^Sc^ mutants under conditions without stress. In contrast, nisin-stressed WT cells showed a significantly higher rate of cell death compared with both nisin-stressed mutants. **e**,**f**, To test the induction of cell death under other stress conditions, the same strains were treated with the protonophore CCCP (10 µM, or 0.002% DMSO as mock control) (**e**) or UV light (**f**) for 10 min. Similar to nisin stress, we detected a significant difference in cell death induction between WT and both CIS^Sc^ mutants. In **c**–**f**, superplots show the ratio of live to total hyphae. The three different colours (green, grey and pink) indicate datasets obtained from three biological replicate experiments. Black line indicates the mean ratio derived from biological triplicate experiments (*n* = 100 images for each experiment). NS, not significant, ****P < 0.0001, determined using a one-way analysis of variance (ANOVA) and Tukey’s post-test. See Extended Data Fig. 6a,b for representative fLM images.

During non-stress conditions, the WT, ΔCIS and CIS-N5 mutant strains displayed a similar sfGFP/FM5-95 ratio, indicating no important difference in viability between strains (Fig. 4a,c). In parallel, we challenged the same *S. coelicolor* strains with a sub-lethal concentration of the bacteriocin nisin (1 µg ml^−1^) for 90 min, which causes the formation of membrane pores and eventually disrupts the integrity of the cell envelope^33^. In the WT, we found that ~50% of the analysed hyphae displayed signs of cell death (Fig. 4b,d). Strikingly, in the CIS-deficient strain and the non-contractile CIS^Sc^ mutant, nisin-stressed cells showed higher viability than WT cells (Fig. 4b,d).

**Extended Data Fig. 6:**
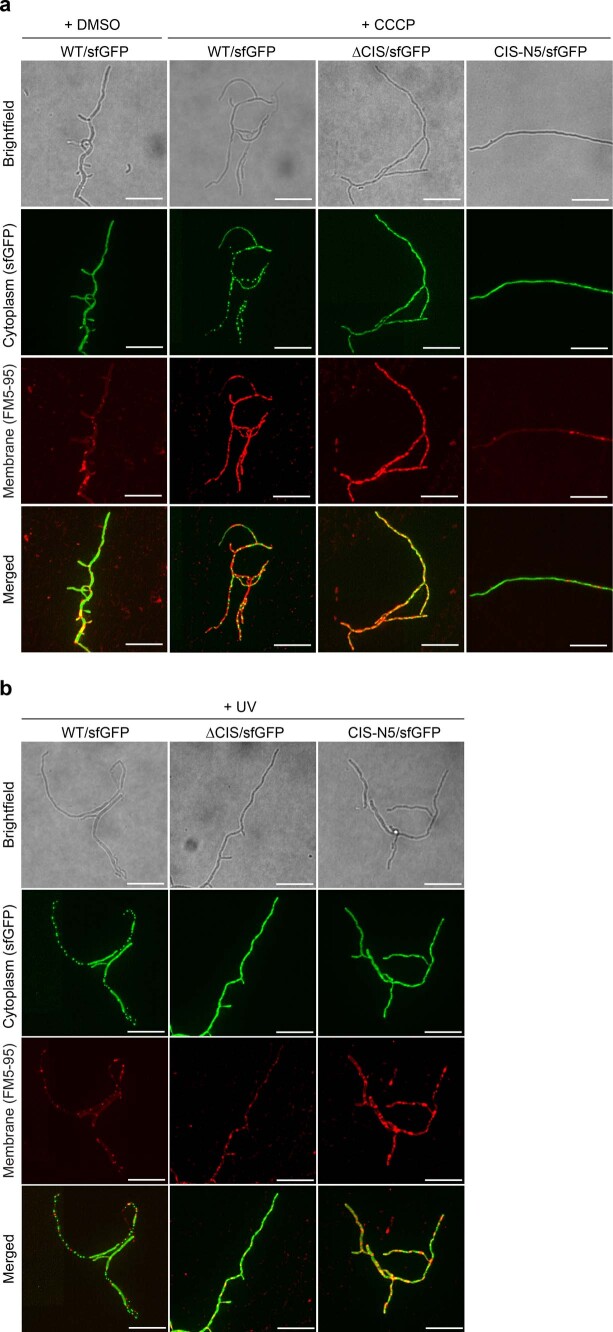
Functional CIS^Sc^ production promotes stress-induced cell death. **a/b**. fLM (shown are representative images) was used to determine the ratio between live cells (cytoplasmic sfGFP) and total cells (membrane dye FM5-95) after growth in the presence of 10 µM CCCP (or 0.002 % DMSO as mock control) (a), or after exposure to UV light (b). *S. coelicolor* WT/sfGFP, ∆CIS/sfGFP and CIS-N5/sfGFP were grown in TSB for 48 h and treated with CCCP or DMSO for 90 min or were exposed to UV light for 10 min. Bars, 10 µm. The quantification for both experiments is shown in Fig. 4e/f.

**Extended Data Fig. 7:**
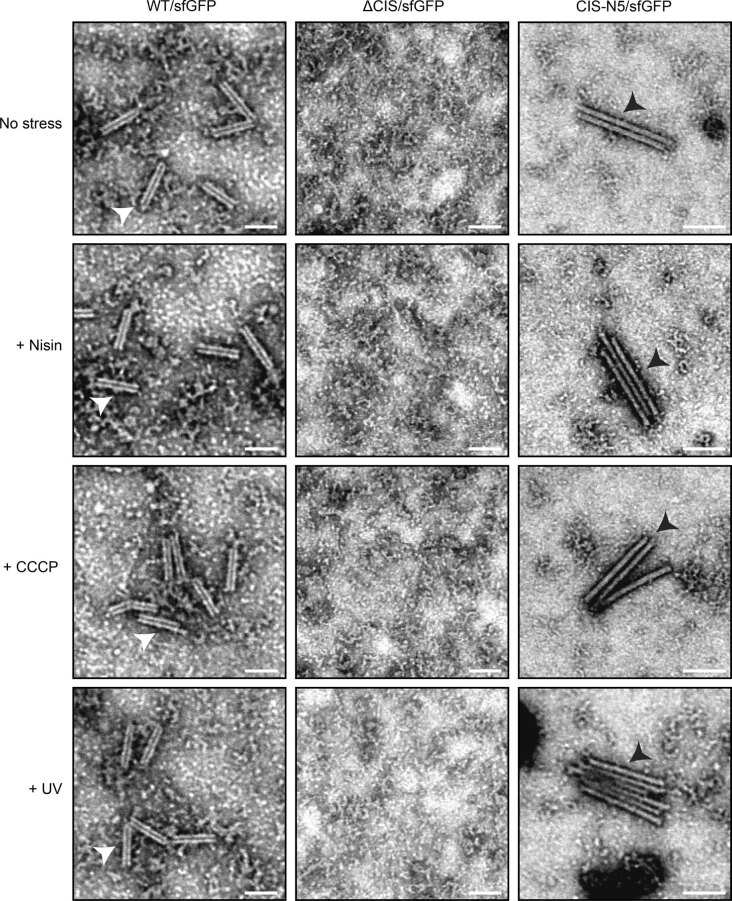
Cell envelope and UV stress do not affect the overall appearance of purified CIS^Sc^ particles. Negative-stain electron micrographs of CIS particles purified from *S. coelicolor* WT/sfGFP, ∆CIS/sfGFP and CIS-N5/sfGFP exposed to no stress, 1 µg/ml nisin, 10 µM CCCP and UV treatment. Arrowheads indicate contracted (white) and extended (black) CIS^Sc^. These experiments were performed three independent times. Bars, 120 nm.

**Extended Data Fig. 8:**
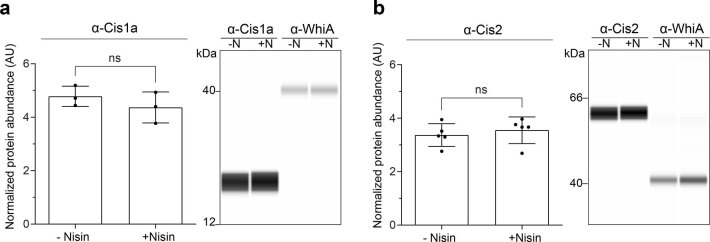
Nisin-stress does not lead to increased CIS^Sc^ production. **a/b**. Quantification (left) and automated Western blot (right) analysis, showing the abundance of Cis1a (a) and Cis2 (b) in WT *S. coelicolor* cell lysates in the presence of nisin (+N) and absence of nisin (-N). Cells were grown in TSB for 48 h, followed by treatment with 1 µg/ml nisin for 90 min. Equal amounts of total protein were subjected to automated Western blot analysis and probed with α-Cis1a, α-Cis2 and α-WhiA polyclonal antibodies. Analysis was performed in biological replicate experiments (n = 3 for Cis1a and n = 5 for Cis2). Cis1a/2 protein levels were normalized to WhiA levels. Shown are the mean values and standard error. ns (not significant) and *p*-value was determined using a two-tailed *t*-test.

To investigate whether other stress factors could induce cell death, we also challenged *S. coelicolor* with the membrane depolarizing agent carbonyl cyanide 3-chlorophenylhydrazone (CCCP) and with UV radiation to induce DNA damage (Extended Data Fig. 6a,b). In line with our previous results, the treatment of vegetative hyphae with both CCCP and UV radiation led to an increase in cell death by 25% in the WT. Treatment of the WT, the ΔCIS or the CIS-N5 mutant strain with 0.002% DMSO, which was used as a mock control in the CCCP stress experiments, did not impact hyphal viability (Fig. 4e,f). In parallel, we also purified CIS^Sc^ from crude cell extracts obtained from non-stressed and stressed samples that were used for fLM imaging. By negative-stain EM imaging, we confirmed the presence of CIS^Sc^ particles in hyphae of the WT and in the CIS-N5 mutant strain, and the absence of sheath particles in the ΔCIS mutant (Extended Data Fig. 7). The abundance of CIS^Sc^ in non-stressed and stressed samples was comparable, which was also confirmed by the level of Cis1a/2 proteins in untreated and nisin-treated hyphae (Extended Data Fig. 8a,b). Taken together, these results indicate that CIS^Sc^ contraction mediates cell death under exogenous stress conditions.

### CIS contribute to multicellular development

**Fig. 5:**
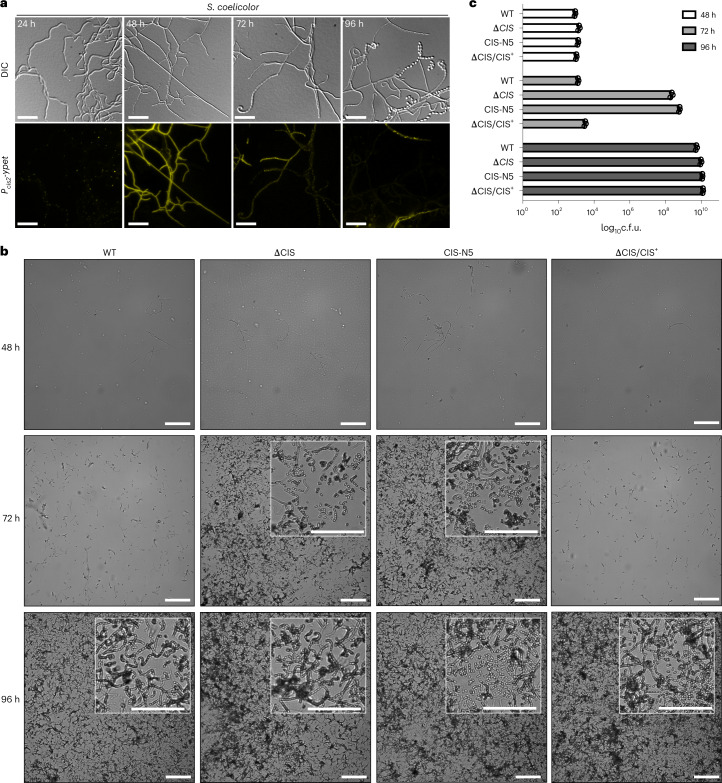
Functional CIS^Sc^ are involved in *S. coelicolor* multicellular development. **a**, Microscopic analysis of WT *S. coelicolor* cells expressing a fluorescent promoter fusion to the sheath promoter *p*_*cis2*_-*ypet in trans*, showing that the sheath operon of the CIS^Sc^ cluster is predominantly expressed during vegetative growth (48 h). Shown are representative micrographs of surface-grown *S. coelicolor* hyphae that were attached to a microscopic cover glass inserted into the inoculated agar surface at a 45° angle. Plates were incubated over 96 h at 30 °C and imaged at the indicated timepoints. Experiments were performed in biological triplicates. Scale bars, 10 µm. **b**, Representative brightfield images of surface imprints of plate-grown colonies of WT *S. coelicolor*, the CIS^Sc^ mutant strains ∆CIS and CIS-N5, and the complemented mutant ∆CIS/CIS^+^. Images were taken at the indicated timepoints. Only hyphae undergoing sporulation or spores attached to the hydrophobic cover glass surface. Insets show magnified regions of the colony surface containing spores and spore chains. Note that strains with functional CIS sporulate later. Scale bars, 50 µm. **c**, Quantification of spore production (c.f.u.) in the same strains as above, revealing much higher c.f.u.s (spores) at 72 h in both CIS mutants. Strains were grown on R2YE agar and spores were collected after 48 h, 72 h and 96 h of incubation. Data show mean ± s.d. obtained from biological triplicate experiments.

**Extended Data Fig. 9:**
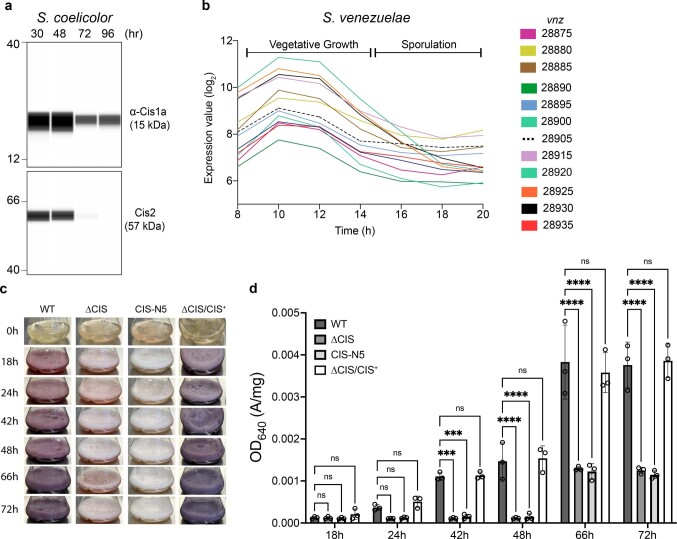
*Streptomyces* CIS proteins are expressed during vegetative growth and impact secondary metabolite production. **a**. Automated Western blot showing the expression of Cis1a (inner tube) and Cis2 (sheath) in hyphae of WT *S. coelicolor* over a time-course of 96 h. *S. coelicolor* was grown on cellophane discs on top of R2YE agar. Automated Western blot analysis was performed in biological duplicate experiments. Equal amounts of protein lysate were loaded and Cis1a and Cis2 were detected using polyclonal α-Cis1a and α-Cis2 antibodies. **b**. Transcription profile of the *S. venezuelae* CIS gene cluster over the entire life cycle^34^. **c**. Comparison of the coloration pattern of *S. coelicolor* WT, the CIS^Sc^ mutant strains ∆CIS, CIS-N5 and the complemented mutant ∆CIS/CIS^+^ in R2YE liquid media. Coloration is indicative of actinorhodin (purple) and undecylprodigiosin (red) production^36^. Note the difference in coloration between WT/complementation mutant as compared to both CIS^Sc^ mutants. Images of each culture flask were taken at the indicated time points. **d**. Quantification of total actinorhodin production of the samples shown in (c). The optical density OD_640_ is an indicator for actinorhodin production^67^. OD_640_ of the culture supernatants was measured and normalized to pellet weight. Analysis was performed in biological triplicate experiments. Shown are the mean values and standard error. ns (not significant), *** (p < 0.001) and **** (p < 0.0001) were calculated using one-way ANOVA and Tukey’s post-test.

Earlier studies indicated that the expression of the *S. coelicolor* CIS gene cluster is coordinated with the *Streptomyces* life cycle^34^. To follow the expression of CIS^Sc^ during the developmental life cycle, we constructed a fluorescent reporter strain in which expression of *ypet* was driven by the *cis2* promoter (*P*_*cis2*_*-ypet*). Since *S. coelicolor* only completes its spore-to-spore life cycle when grown on solid medium, glass coverslips were inserted at a 45° angle into agar plates inoculated with spores. Coverslips with attached *S. coelicolor* hyphae were removed and imaged every 24 h for 4 d by fLM. Fluorescent signal indicated that the *cis2* promoter was primarily active in vegetative hyphae at the 48-h timepoint (Fig. 5a). In parallel, we determined CIS^Sc^ protein levels in surface-grown WT *S. coelicolor* over the life cycle via western blot analysis. Consistent with our fluorescence reporter experiment, Cis1a/2 levels were highest in vegetative mycelium that was collected after 30 h and 48 h of incubation (Extended Data Fig. 9a). These results are also in agreement with published transcriptomics data from *S. venezuelae* showing the specific induction of the CIS^Sv^ gene cluster during vegetative growth (Extended Data Fig. 9b)^35^.

Since a previous study on *S. lividans* reported a putative role of CIS in interspecies interactions^20^, we performed a series of growth competition assays but did not observe differences in fitness between the WT and CIS^Sc^ mutants (Supplementary Table 3). We then tested whether the expression of a functional CIS^Sc^ influenced the timely progression of the *S. coelicolor* life cycle, using WT, ΔCIS, CIS-N5 and a complemented strain (∆CIS/CIS^+^). First, we detected sporulating hyphae and spores by imaging surface imprints of plate-grown (R2YE agar) colonies at different timepoints. All strains consistently completed their life cycle and synthesized spores (Fig. 5b). Importantly, in contrast to the WT and the complemented strain, both ΔCIS and CIS-N5 mutants sporulated markedly earlier (72 h vs 96 h for the WT and the complemented mutant). These results were further corroborated by quantifying the number of spores produced by the individual strains under the same experimental conditions (Fig. 5c), indicating that CIS^Sc^ play a role in regulating the *S. coelicolor* vegetative growth cycle.

In addition to the accelerated cellular development in the CIS^Sc^ mutants, we also observed that the production of the two characteristic pigmented secondary metabolites in *S. coelicolor*, actinorhodin (blue)^36^ and undecylprodigiosin (red)^37^, was notably reduced compared to the WT and the complemented ∆CIS/CIS^+^ mutant (Extended Data Fig. 9c). This discrepancy was further confirmed by quantifying the total amount of actinorhodin (intracellular and secreted) produced by the different strains over a period of 72 h. Both ∆CIS and CIS-N5 mutants produced approximately 70% less actinorhodin compared to the WT and the ΔCIS complementation strain (Extended Data Fig. 9d). Moreover, in contrast to the observed delay in sporulation, actinorhodin production in the CIS^Sc^ mutants was not only delayed but also never reached WT levels in the time frame of this experiment.

Altogether, we have shown that deleting or expressing non-functional CIS^Sc^ results in important changes in the *S. coelicolor* life-cycle progression, which also affects secondary metabolite production.

### Sco4256, Sco4257 and Sco4258 are CIS-associated effectors

**Fig. 6:**
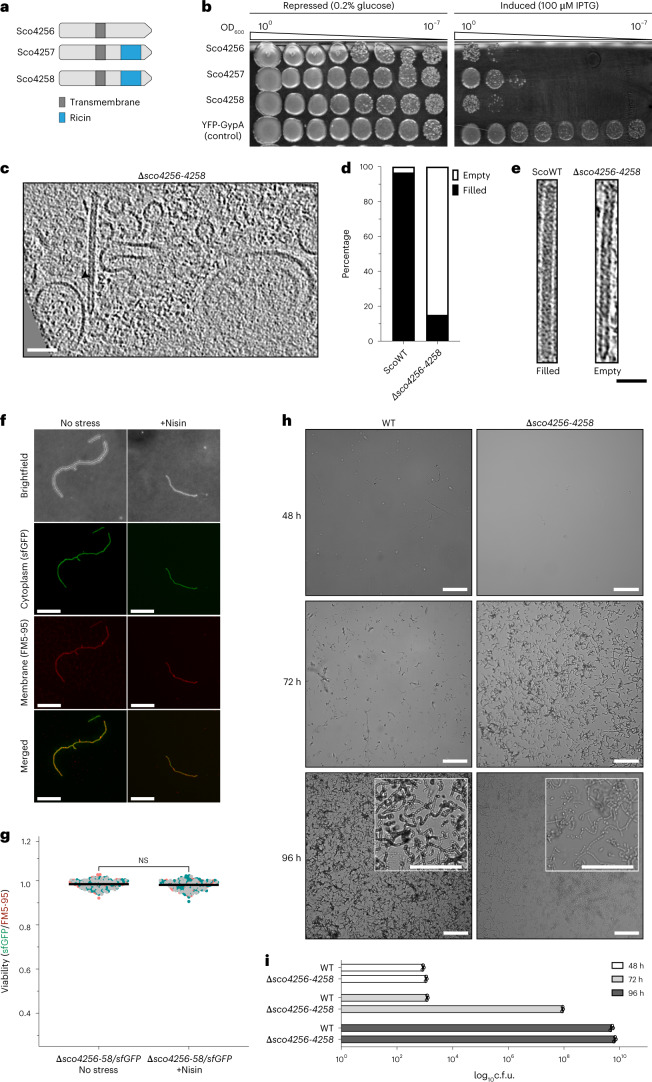
Identification and characterization of CIS^Sc^ effectors (Sco4256–4258). **a**, Schematic showing the domain organization of Sco4256, Sco4257 and Sco4258. All three proteins include one predicted transmembrane domain. Sco4257/4258 contain a ricin-like domain on the C terminus. **b**, Survival of *E. coli* expressing Sco4256, Sco4257, Sco4258 or YFP-GypA (used as a negative control of toxicity). Images are representatives of three independent experiments. **c**, CryoET slice of a hypha from the *S. coelicolor* effector-deficient mutant (∆*sco4256–4258*), showing empty CIS^Sc^ but otherwise WT-like particles (arrowhead). Tomograms (90) were collected from 3 independent datasets. Scale bars, 75 nm. **d**, Fractions of empty CIS^Sc^ particles are increased in the effector mutant compared with the WT (ScoWT, *n*_total_ = 91; *∆sco4256–4258*, *n*_total_ = 73). **e**, Representative images of filled and empty CIS^Sc^ particles. Tomograms (60) were collected from 2 independent datasets. Scale bars, 50 nm. **f**, fLM (shown are representative images) was used to determine the ratio between live cells (cytoplasmic sfGFP) and total cells (membrane dye FM5-95) after growth in the absence of stress or in the presence of nisin stress for *S. coelicolor ∆sco4256–58*/sfGFP. Scale bars, 10 µm. **g**, The quantification of the experiments in **f** showed no notable difference between the ∆*sco4256–58*/sfGFP strain grown without stress and with nisin. Superplots show the area ratio of live to total hyphae. The three different colours (green, grey and pink) indicate the three biological repeats. Black line indicates the mean ratio derived from biological triplicate experiments (*n* = 100 images for each experiment). Significance was calculated using one-way ANOVA and Tukey’s post-test. **h**, Representative brightfield images of surface imprints of plate-grown colonies of WT *S. coelicolor* (from Fig. 5b) and the CIS^Sc^ effector mutant strain ∆*sco4256–4258*. Images were taken at the indicated timepoints. Insets show magnified regions of the colony surface containing spores and spore chains. Note that strains with functional CIS sporulate later. Scale bars, 50 µm. **i**, Quantification of spore production (c.f.u.) in the same strains as above, revealing much higher c.f.u.s (spores) at 72 h in the effector-deficient CIS mutant. Data show mean ± s.d. obtained from biological triplicate experiments.

Most T6SS and eCIS gene clusters encode effector proteins, which are delivered to kill or manipulate the cellular activity of the corresponding target^38^. We noticed that the CIS^Sc^ gene cluster encodes three uncharacterized proteins Sco4256–4258. All three proteins consist of a single transmembrane domain, a highly similar N terminus and a C-terminal domain, which in the case of Sco4257/4258, consists of a predicted carbohydrate-binding moiety (RICIN-type beta trefoil) (Fig. 6a). Interestingly, we detected at least one homologue of such potential effectors in 47 out of 94 sequenced *Streptomyces* strains that also encode a CIS gene cluster^8,9^, while CIS-negative strains typically do not encode these potential effectors (Supplementary Table 4). To characterize these proteins, we expressed the individual genes in *E. coli* from an inducible promoter and assessed cell viability. Upon induction, the Sco4256–4258-producing strains showed notably reduced viability, indicating toxic activity of the proteins (Fig. 6b).

**Extended Data Fig. 10:**
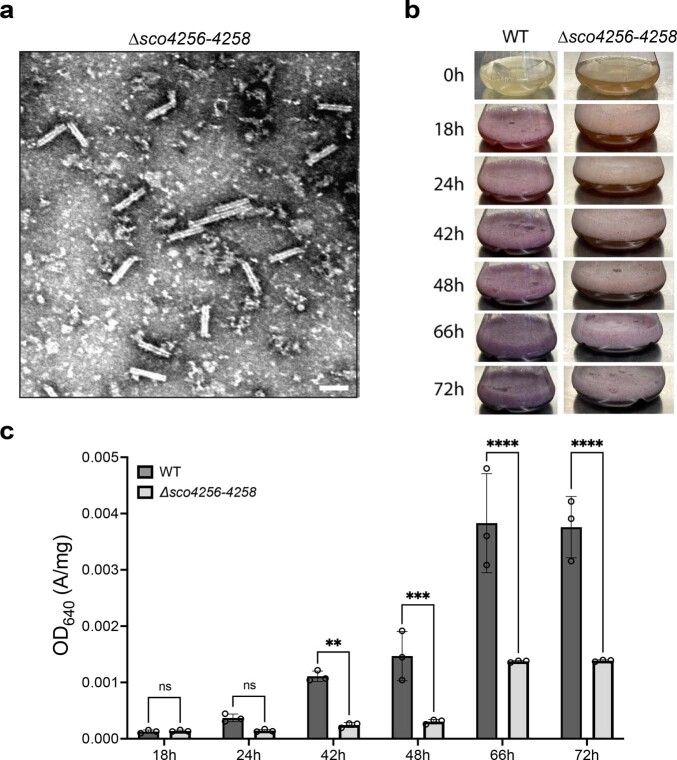
*Streptomyces* CIS effectors impact secondary metabolite production. **a**. Negative-stain electron micrographs of CIS particles purified from *S. coelicolor* ∆*sco4256-4258* (SS540). This experiment was performed in three biological replicates. Bars, 120 nm. **b**. Comparison of the coloration pattern of *S. coelicolor* WT (Extended Data Fig. 9c) and the CIS^Sc^ effector mutant strains ∆*sco4256-4258* in R2YE liquid media. Coloration is indicative of actinorhodin (purple) and undecylprodigiosin (red) production^36^. Note the difference in coloration between the WT and the effector mutant (∆*sco4256-4258)*. Images of each culture flask were taken at the indicated time points. **c**. Quantification of total actinorhodin production of the samples shown in (b). The optical density OD_640_ is an indicator for actinorhodin production^67^. OD_640_ of the culture supernatants was measured and normalized to pellet weight. Analysis was performed in biological triplicate experiments. Shown are the mean values and standard error. ns (not significant), ** (p < 0.01), *** (p < 0.001) and **** (p < 0.0001) were calculated using one-way ANOVA and Tukey’s post-test.

To explore whether Sco4256–4258 also played a role in CIS^Sc^-mediated cell death, we generated an *S. coelicolor* triple mutant (Δ*sco4256–4258*). We first confirmed that the mutation did not affect the assembly of CIS^Sc^ particles by negative-stain EM (Extended Data Fig. 10a) and cryoET (Fig. 6c). The general in situ structure of CIS^Sc^ particles in the Δ*sco4256–4258* mutant was similar to WT, with a notable exception regarding their luminal density. While the tube lumen was mostly filled in WT CIS^Sc^ (~97%, *n*_total_ = 91), the tube lumen appeared mostly empty in mutant CIS^Sc^ (~85%, *n*_total_ = 73) (Fig. 6d,e).

Using this triple mutant, we repeated the fLM-based viability assay upon nisin stress (Fig. 6f,g). Unlike the drop in viability of the WT, we found no notable difference in the viability of the Δ*sco4256–4258* mutant upon nisin stress, which was consistent with the pattern observed in ΔCIS and CIS-N5 mutants (Fig. 4b,d). Finally, Δ*sco4256–4258* cells also sporulated markedly earlier (Fig. 6h,i) and produced about 79% less actinorhodin (Extended Data Fig. 10c), which is again similar to the previously analysed CIS^Sc^ mutants (Fig. 5b,c and Extended Data Fig. 9c,d). Taken together, these results show that Δ*sco4256–4258* cells phenocopy the ΔCIS and CIS-N5 mutants, supporting the idea of Sco4256–4258 being CIS^Sc^-associated effectors.

## Discussion

Here we show that CIS particles from *Streptomyces* are functionally distinct from related eCIS and T6SS. Our data from fLM imaging, cryoET and western blotting all consistently indicate that CIS^Sc^ were assembled free floating in the cytoplasm; however, under our experimental conditions, they were neither found to be released into the medium, nor were they observed attached to the cytoplasmic membrane. This argues against a typical eCIS mode of action, and it is also inconsistent with a typical T6SS mode of action; in particular, it is difficult to conceptualize how a T6SS would fire through the thick peptidoglycan cell wall in a Gram-positive host organism. Therefore, our data points to an intracellular function, which is supported by further observations that are discussed below.

CryoET imaging revealed a notable fraction of partially or fully lysed cells in a vegetative culture. Interestingly, the degree of cell lysis strongly correlated with the presence of contracted CIS^Sc^ assemblies. These results were further supported by fLM imaging, demonstrating that under different types of exogenous stress conditions, cell death was induced in the WT at a much higher percentage than in mutants that expressed non-functional CIS^Sc^. Thus, CIS^Sc^ contraction is required for inducing cell death once a culture encounters stress.

We propose that cell lysis is mediated by cell envelope-targeting effectors that are released from the CIS^Sc^ upon contraction; however, how are these effectors delivered? This could happen by the firing of a free-floating CIS^Sc^, releasing loaded effectors into the host cytoplasm. An alternative mechanism could be (transient) CIS^Sc^ binding to the host cytoplasmic membrane, followed by contraction. Membrane binding in eCIS is typically mediated by tail fibres that are attached to the baseplate complex. Oftentimes, tail fibre genes are found just downstream of baseplate components^15,18,24^. Interestingly, we detected the conserved protein Sco4242 just downstream of the putative baseplate genes *cis11* and *sco4243* (Fig. 1b). Bioinformatic analyses revealed the presence of Sco4242 homologues in 79% of CIS-positive *Streptomyces* strains and the absence of such homologues in CIS-negative strains (Supplementary Table 4). Sco4242 consists of a predicted transmembrane segment and a conserved sugar-binding domain. Together with the absence of typical tail fibre-like proteins and structures, we speculate that Sco4242 may play a role as an adaptor that mediates binding of CIS^Sc^ to the cytoplasmic membrane upon a stress signal, followed by firing.

In addition to mediating death of the host cell in response to stress, we showed that CIS^Sc^ contraction also plays a role in the timely progression of the *Streptomyces* life cycle, as evidenced by the earlier onset of sporulation in the CIS^Sc^ and effector mutants. Cell death has been proposed as a distinct process in the developmental programme of *Streptomyces*^39^. However, the underlying molecular mechanism remains unclear. We speculate that contracting CIS^Sc^ could induce hyphal cell death, which may lead to the release of nutrients that result in the postponement of subsequent life-cycle stages, thereby impacting multicellular development. Notably, increased cell death has been reported to occur at the centre of colonies^40,41^; these regions are thought to be limited in nutrient and/or oxygen supply, which in turn may be perceived as stress and trigger CIS^Sc^-mediated cell death.

Notably, the morphological differentiation of *Streptomyces* colonies is tightly coordinated with the production of secondary metabolites, which are often secreted into the environment where they can provide a competitive advantage^21^. We showed that CIS^Sc^ mutants were not only affected in the timing of the onset of sporulation, but also in the production of the secondary metabolite actinorhodin. We speculate that the delay of sporulation in the WT (and the complemented strain) may be advantageous to allow the coordinated production and release of key secondary metabolites such as toxins, proteases or signalling molecules. The lack of functional CIS^Sc^ in both mutant strains could lead to improper timing of cell cycle progression, resulting in early sporulation, which may in turn lead to lower amounts of actinorhodin production.

In conclusion, our data provide new functional insights into CIS in a Gram-positive model organism and a framework for studying new intracellular roles of CIS, including regulated cell death and life-cycle progression.

## Methods

### Bacterial strains, plasmids and oligonucleotides

Bacterial strains, plasmids and oligonucleotides can be found in Supplementary Tables 5 and 6. *E. coli* strains were cultured in LB, SOB or DNA medium. *E. coli* cloning strains TOP10 and DH5α were used to propagate plasmids and cosmids. *E. coli* strain BW25113/pIJ790 was used for recombineering cosmids^42^. For interspecies conjugation, plasmids were transformed into *E. coli* ET12567/pUZ8002. Where necessary, media were supplemented with antibiotics at the following concentrations: 100 µg ml^−1^ carbenicillin, 50 µg ml^−1^ apramycin, 50 µg ml^−1^ kanamycin and 50 µg ml^−1^ hygromycin.

*S. coelicolor* and *S. venezuelae* strains were cultivated in LB, MYM, TSB, TSB-YEME or R2YE liquid medium at 30 °C in baffled flasks or flasks with springs, at 250 r.p.m. or grown on LB, MYM, SFM, R2YE medium solidified with 1.5% (w/v) Difco agar^43^. Where necessary, media were supplemented with antibiotics at the following concentrations: 25 µg ml^−1^ apramycin, 5 µg ml^−1^ kanamycin, 25 µg ml^−1^ hygromycin and 12.5–25 µg ml^−1^ nalidix acid.

### Generation of *Streptomyce*s mutant strains

The *λ* RED homologous recombination system was used to isolate gene replacement mutations using PCR-directed mutagenesis (ReDirect) of the *S. coelicolor* cosmid StD-49, StD8A and the *S. venezuelae* cosmid Pl1-F14 containing the CIS gene cluster^42,44^. Genes encoding the sheath (*sco4253*, *vnz_28920*), the whole CIS-sheath operon (*sco4253-SCO4251*, *vnz_28920-28910*) or the putative effectors (*sco4256–sco4258*) were replaced with the *aac3(IV)-oriT* resistance cassette from pIJ773. Mutagenized cosmids (pSS480, pSS481, pSS489, pSS490, pSS703) were transformed and subsequently conjugated from *E. coli* ET12567/pUZ8002 to wild-type *S. coelicolor* or *S. venezuelae*. Exconjugants that had successfully undergone double-homologous recombination were identified by screening for apramycin-resistance and kanamycin sensitivity. Deletion of the respective CIS mutant genotypes was subsequently verified by PCR.

### Phylogenetic analysis

Phylogenetic analysis of the different contractile injection systems (from eCIS, T6SS, phage and CIS from *Streptomyces*) was conducted using the putative sheath proteins. Alignment and generation of the phylogenetic tree was performed as previously reported^7,15^. First, the amino acid sequences from 16 sheath proteins were aligned using the MUSCLE online tool^45,46^. Standard parameters were applied for multiple sequence alignment. Then, the MEGAX programme^47^ was used to reconstruct phylogenetic trees using the maximum likelihood (ML) method and bootstrap values (1,000 resamples) were applied to assess the robustness of the tree.

### Sheath preparation of CIS from *Streptomyces*

*S. venezuelae* was cultivated either in 30 ml LB or MYM liquid medium for 14 h. *S. coelicolor* strains were grown in 30 ml TSB, TSB-YEME or R2YE liquid medium for 48 h. *Streptomyces* cultures were pelleted by centrifugation (7,000 × *g*, 10 min, 4 °C), resuspended in 5 ml lysis buffer (150 mM NaCl, 50 mM Tris-HCl, 0.5× CellLytic B (Sigma-Aldrich), 1% Triton X-100, 200 µg ml^−1^ lysozyme, 50 μg ml^−1^ DNAse I, pH 7.4) and incubated for 1 h at 37 °C. Cell debris was removed by centrifugation (15,000 × *g*, 15 min, 4 °C) and cleared lysates were subjected to ultra-centrifugation (150,000×g, 1 h, 4 °C). Pellets were resuspended in 150 µl resuspension buffer (150 mM NaCl, 50 mM Tris-HCl, supplemented with protease inhibitor cocktail (Roche), pH 7.4). Proteins in the CIS preparation were subjected to negative-stain EM imaging^48^ and mass spectrometry at the Functional Genomics Center Zürich.

### Negative-stain electron microscopy

Purified sheath particles (4 µl) were adsorbed to glow-discharged, carbon-coated copper grids (Electron Microscopy Sciences) for 60 s, washed twice with milli-Q water and stained with 2% phosphotungstic acid for 45 s. The grids were imaged at room temperature using a Thermo Fisher Morgagni transmission electron microscope (TEM) operated at 80 kV.

### Mass spectrometry analysis

To confirm the presence of predicted CIS components from *Streptomyces*, isolated sheath particles were subjected to liquid chromatography–mass spectrometry analysis (LC–MS/MS). First, the samples were digested with 5 µl of trypsin (100 ng µl^−1^ in 10 mM HCl) and microwaved for 30 min at 60 °C. The samples were then dried, dissolved in 20 µl double-distilled water (ddH_2_0) with 0.1% formic acid, diluted at 1:10 and transferred to autosampler vials for liquid chromatography with tandem mass spectrometry analysis. A total of 1 µl was injected on a nanoAcquity UPLC coupled to a Q-Exactive mass spectrometer (Thermo Fisher). Database searches were performed using the Mascot swissprot and tremble_streptomycetes search programme. For search results, stringent settings were applied in Scaffold (1% protein false discovery rate, a minimum of two peptides per protein, 0.1% peptide false discovery rate). The results were visualized using the Scaffold software (Proteome Software, v4.11.1).

### Automated western blot analysis

Automated western blot analysis (WES) of liquid-grown *Streptomyces* strains was essentially performed as described previously^49^ using the anti-rabbit secondary antibody detection module (Protein Simple DM-001). Cell pellets were resuspended in 0.4 ml sonication buffer (20 mM Tris, pH 8.0, 5 mM EDTA, 1× EDTA-free protease inhibitors (Sigma-Aldrich)) and subjected to sonication at 4.5 µm amplitude for 7 cycles of 15 s on/15 s off. Samples were centrifuged at 17,000 × *g* for 15 min at 4 °C. The supernatants were removed and subjected to a Bradford Assay (Biorad). Equivalent total protein concentrations (0.2 mg ml^−1^) were assayed using the automated western blotting machine WES (Protein Simple) according to the manufacturer’s guidelines. For the detection of Cis1a and Cis2 proteins, antibodies for α-Cis1a (GenScript) and α-Cis2 (GenScript) were used at a concentration of 1:200. For detection of WhiA, 0.5 μg of total protein and anti-WhiA (Polyclonal) at 1:100 dilution was used^50^.

For the detection of Cis1a and Cis2 in culture supernatants, WT *S. coelicolor*, SS387 and SS395 were grown in duplicate in TSB medium for 48 h. Cultures were pelleted and 20 ml supernatants obtained from each culture were concentrated to approximately 1 ml using Amicon Ultra-15 10K spin column (Millipore). Total protein samples were further processed as described above. In parallel, an aliquot of each sample was loaded onto a 12% Teo-Tricine/SDS precast protein gel (Expedian) to demonstrate the presence of proteins in the culture supernatants. SDS gels were stained with InstantBlue (Sigma-Aldrich) and scanned.

For the automated western blot analysis of surface-grown *S. coelicolor* samples from R2YE plates, mycelium was scraped off sterile cellophane discs that had been placed on top of solid R2YE medium. Mycelia were removed at the described timepoints and washed with 1X PBS. The supernatant was discarded and the pellet frozen. Pellets were treated and WES run as above. All virtual western blots were generated using the Compass software for simple western (v6.0.0). Data on protein abundance were plotted using GraphPad Prism (v9.3.1).

For WES analyses of Cis1a and Cis2 abundance following nisin stress, WT *S. coelicolor* cultures were grown in TSB medium at 30 °C for 48 h, after which they were split and normalized to the same optical density. To one culture replicate, nisin was added to a final concentration of 1 µg ml^−1^ and to the other, the diluent (0.05% acetic acid) was added in equal volume. Next, 2 ml aliquots were removed from each sample and pelleted at 17,000 × *g*. Pellets were treated as above but were additionally probed with an α-WhiA antibody at 1:100 concentration. The band intensities for Cis1a and Cis2 were normalized against the band intensity of WhiA and plotted in GraphPad Prism (v9.3.1) with the mean ± s.d.

### Fluorescence light microscopy and image analysis

For imaging protein localization and fluorescent promoter reporter fusion in *S. coelicolor*, a Zeiss Axio Observer Z.1 inverted epifluorescence microscope fitted with an sCMOS camera (Hamamatsu Orca FLASH 4), a Zeiss Colibri 7LED light source, a Hamamatsu Orca Flash 4.0v3 sCMOS camera and a temperature-controlled incubation chamber was used. Images were acquired using a Zeiss Alpha Plan-Apo ×100/1.46 Oil DIC M27 objective with YFP excitation/emission bandwidths of 489–512 nm/520–550 nm. Still images and time-lapse image series were collected using Zen Blue (Zeiss) and analysed using Fiji^51^.

To monitor the activity of the fluorescent sheath promoter fusion in *S. coelicolor*, strain SS484 spores were spotted onto solid R2YE medium and grown alongside a microscope coverslip that had been inserted into the agar at approximately a 45° angle. Plates were incubated at 30 °C for up to 4 d. At the indicated timepoints, glass coverslips with attached hyphae were removed, mounted onto slides affixed with 1% agar pads and imaged.

For time-lapse imaging of *S. coelicolor* expressing a fluorescently labelled sheath protein (SS389), cells were first grown in TSB-YEME for 40 h and a 2 μl sample of the culture was immobilized on a 1% agarose pad prepared with filtered culture medium and using a gene frame (Thermo Fisher). Experiments were performed at 30 °C and growing hyphae were imaged every 5 min. Image collection and analysis were performed using Zen Blue (Zeiss) and Fiji, respectively^51^.

### Plunge freezing of *Streptomyces* hyphae

For cryoET, *Streptomyces* cells were mixed with 10 nm Protein A conjugated colloidal gold particles (1:10 v/v, Cytodiagnostics) and 4 µl of the mixture was applied to a glow-discharged holey-carbon copper EM grid (R2/1 or R2/2, Quantifoil). The grid was automatically blotted from the backside for 4–6 s in a Mark IV Vitrobot using a Teflon sheet on the front pad, and plunge-frozen in a liquid ethane-propane mixture (37%/63%) cooled by a liquid nitrogen bath.

For single-particle cryoEM (SPA), the *S. coelicolor* CIS particles (from WT CIS and non-contractile CIS) collected after sheath preparation were vitrified using a Vitrobot Mark IV (Thermo Fisher). Samples (4 µl) were applied on glow-discharged 200 mesh Quantifoil gold grids (R2/2). Grids were blotted for 5 s and plunged into liquid ethane-propane mix (37%/63%). Frozen grids were stored in liquid nitrogen until loading into the microscope.

### Cryo-focused ion beam milling

A standard protocol was used to perform cryo-focused ion beam milling (CryoFIB milling) of *S. venezuelae*^52^. Plunge-frozen grids were clipped into cryoFIB-autoloader grids (Thermo Fisher), then transferred into a liquid nitrogen bath of a loading station (Leica Microsystems) and mounted into a 40° pre-tilted scanning electron microscope (SEM) grid holder (Leica Microsystems). The holder was transferred with a VCT100 cryo-transfer system (Leica Microsystems) into a Helios NanoLab600i dual beam FIB/SEM (Thermo Fisher). Grids were coated with platinum precursor gas for 6 s and checked with the SEM at 3–5 kV (80 pA) to evaluate grid quality and identify targets. Lamellae were milled in multiple steps using the focused gallium ion beam (43 nA to 24 pA) until a thickness of ~250 nm was achieved. The holder was returned to the loading station using the VCT100 transfer system. Unloaded grids were stored in liquid nitrogen before cryoET imaging.

### CryoET

Intact or cryoFIB-milled *Streptomyces* cells were imaged by cryoET^53^. Images were recorded on Titan Krios 300 kV microscopes (Thermo Fisher) equipped with a Quantum LS imaging filter operated at a 20 eV slit width and with K2 or K3 Summit direct electron detectors (Gatan). Tilt series were collected using a bidirectional tilt scheme from −60 to +60° in 2° increments. Total dose was 130–150 e^−^ Å^−2^ and defocus was kept at −8 µm. Tilt series were acquired using SerialEM^54^, drift-corrected using alignframes, reconstructed and segmented using the IMOD programme suite^55^. To enhance contrast, tomograms were deconvolved with a Wiener-like filter ‘tom_deconv’^56^.

### SPA data collection and image processing

CryoEM datasets of *S. coelicolor* contracted sheath and extended sheath-tube module were collected as movie stacks using the SerialEM programme on the Titan Krios EM operating at 300 kV and equipped with an energy filter and a K2 Summit camera. The movie frames of each collected stack were aligned and summed up into one single micrograph with dose weighting at the binning factor of 2 using MotionCor2 (ref. ^57^). The contrast transfer function (CTF) parameters of the micrographs were estimated using Gctf. Pixel size at specimen level was 1.4 Å and target defocus ranged from −1.5 µm to −3.5 µm. Each stack contains 50 frames, and the accumulated electron dose rate was ~60 e^−^ Å^−2^.

The image processing of contracted sheath and extended sheath-tube from *S. coelicolor* was performed as previously reported^15^. The particles were picked manually using Relion 3.0^58^. The particle extraction was performed in ‘Extract helical segments’ mode to extract helical segments. The structural determination of the contracted sheath and the extended sheath-tube module was performed using helical reconstruction in Relion 3.0^59^.

For the contracted sheath, the final 3.6 Å resolution structure of contracted sheath was obtained from 4,838 particles applied with 6-fold symmetry and helical parameters (rise = 17.22 Å, twist = 26.58°) (Extended Data Fig. 2a).

For the extended sheath-tube module, the final 3.9 Å resolution structure of the extended sheath-tube module was determined from 18,822 particles calculated with 6-fold symmetry and helical parameters (rise = 38.50 Å, twist = 23.10°) (Extended Data Fig. 3e).

The resolutions of relative reconstruction maps were estimated on the basis of the gold-standard Fourier shell correlation (FSC) = 0.143 criterion^60^. The local resolution estimations of individual maps were performed using the local resolution module in Relion 3.0 and examined using UCSF Chimera^61^ (Extended Data Figs. 2b and 3f).

### Structure modelling

Proteins were built de novo using COOT^62^. Models were iteratively refined using RosettaCM^63^ and real-space refinement implemented in PHENIX^64^. Sheath protein could only be partially modelled and in some cases side chains were not assigned. Final model validation was done using MolProbity^64^ and correlation between models and the corresponding maps were estimated using mtriage^64^. All visualizations were done using PyMOL, UCSF Chimera^61^ or ChimeraX^65^.

### Correlative cryo-light and electron microscopy

For correlative cryo-light and electron microscopy, frozen grids containing WT *S. coelicolor* were transferred to a CMS196V3 Linkam cryo-stage and imaged using a ×100 numerical aperture 0.74 objective on an LSM900 Airyscan 2 Zeiss microscope driven by ZEN Blue software (v3.5). Fluorescence images of areas of interest were manually correlated with the corresponding TEM square montage using SerialEM^54,66^.

### Fluorescence-based cell viability assay

To express sfGFP constitutively in *Streptomyces* strains, the coding sequence for sfGFP was introduced downstream of the constitutive promoter ermE* on an integrating plasmid vector (pIJ10257). The plasmid was introduced by conjugation to *S. coelicolor* strains (WT, ∆CIS and CIS-N5, ∆Sco4256–4258). These strains were inoculated into 30 ml of TSB liquid culture and incubated at 30 °C with shaking at 250 r.p.m. in baffled flasks for 48 h. Where appropriate, nisin and CCCP (or 0.002% DMSO) were added to a final concentration of 1 µg ml^−1^ and 10 µM, respectively. Cultures were incubated for a further 90 min. For UV exposure, 10 ml of the *S. coelicolor* cultures was transferred into a Petri dish and treated with Sankyo Denki Germicidal 68 T5 UV-C lamps for 10 min in a Herolab UV DNA crosslinker CL-1. Then, 1 ml aliquots were centrifuged for 5 min at 15,000 × *g*, washed twice with PBS and resuspended in 1 ml PBS with 5 µg ml^−1^ FM5-95 membrane stain. The cell suspension and membrane stain were mixed by vortexing and kept in the dark at room temperature for 10 min. The suspension was then centrifuged for 5 min at 15,000 × *g*, washed twice with PBS and resuspended in 50 µl PBS. Samples (10 µl) were immobilized on 1% agar pads and imaged on the Thunder Imager 3D cell culture microscope (Leica) at room temperature. First, tile scan images were acquired on the LasX Navigator plug-in of Leica Application Suite X (LasX) software (v3.7.4.23463), and 100 targets were picked manually. Then *z*-stack images with the HC PL APO ×100 objective were acquired at an excitation of 475 nm and 555 nm under GFP (green) and TRX (red) filters, respectively. Images were processed using LasX software to apply thunder processing and maximum projection, FIJI to create segmentation and quantify the live (sfGFP)/total cells (FM5-95) area ratio^51^, and statistical analysis was performed on GraphPad Prism 9 (v9.3.1).

### Cover glass impression of *Streptomyces* spore chains

Spore titres of relevant strains were determined by standard techniques. *S. coelicolor* (10^7^ colony forming units (c.f.u.s)) of strains WT, SS387, SS393, SS395 and SS540 were spread onto R2YE agar plates and grown at 30 °C. Sterile glass coverslips were gently applied to the top surface of each bacterial lawn after 48 h, 72 h and 96 h post inoculation. Cover slips were then mounted onto glass microscope slides and imaged using a ×40 objective on a Leica Thunder Imager 3D cell culture microscope. Images were processed using FIJI^51^.

### Actinorhodin production assay

*S. coelicolor* strains (WT, SS387, SS393, SS395 and SS540) were inoculated into 30 ml R2YE liquid media at a final concentration of 1.5 × 10^6^ c.f.u. ml^−1^. Cultures were grown in baffled flasks at 30 °C overnight. Cultures were standardized to an optical density (OD)_450_ of 0.5 and inoculated in 30 ml of fresh R2YE liquid medium. For visual comparison of pigment production, images of the growing culture were taken between *t* = 0 and *t* = 72 h (as indicated in Extended Data Fig. 9c). For quantification of total actinorhodin production, 480 µl of samples were collected at the same timepoints when images were taken. KOH (120 µl of 5 M) was added, samples were vortexed and centrifuged at 5,000 × *g* for 5 min. The weight of each tube was recorded. A Synergy 2 plate reader (Biotek) was then used to measure the absorbance of the supernatant at 640 nm. The absorbance was normalized by the weight of the wet pellet.

### *E. coli* killing assay

*E. coli* Rosetta (DE3) cells carrying the plasmids for the expression of the putative effector genes (*sco4256*, *sco4257* or *sco4258*) or the control construct (*yfp* with a synthetic membrane anchor) were grown in liquid LB medium supplemented with 150 μg ml^−1^ carbenicillin and 0.2% glucose overnight. Optical density of the overnight cultures was adjusted to 1 and serial 10-fold dilutions were spotted on solid LB containing carbenicillin in addition to either 0.2% glucose (repressive conditions) or 100 μM isopropyl β- d-1-thiogalactopyranoside (IPTG) (induction conditions). Plates were scanned after an overnight incubation at 37 °C.

### Reporting summary

Further information on research design is available in the Nature Portfolio Reporting Summary linked to this article.

## Supplementary information

Supplementary Information Supplementary Tables 1–6.

Reporting Summary

Peer Review File

Supplementary Video 1 Time-lapse movie related to Fig. 2g,h, showing the spatiotemporal localization of fluorescently tagged CIS particles in growing *S. coelicolor* hyphae. Note that the hypha displayed in this movie was rotated. Images were acquired every 5 min. Scale bar, 10 μm.

Supplementary Data 1 PDB validation report for 8BKY.

Supplementary Data 2 PDB validation report for 8BL4.

## Data Availability

Representative reconstructed tomograms (EMD-16200, EMD-16201, EMD-16202, EMD-16203, EMD-16204, EMD-16205, EMD-16206, EMD-16208 and EMD-16210) and SPA cryoEM maps (EMD-16098 and EMD-16101) have been deposited in the Electron Microscopy Data Bank. Atomic models (PDB: 8BKY and PDB: 8BL4) have been deposited in the Protein Data Bank.
