# Supplementary material for: Cytoplasmic contractile injection systems mediate cell death in Streptomyces
Source: Nat Microbiol. 2023 Mar 9;8(4):711–26. doi: 10.1038/s41564-023-01341-x (PMC10066040; doi:10.1038/s41564-023-01341-x)
Supplement: Supplementary file 1 — Supplementary Tables 1–6. [file 41564_2023_1341_MOESM1_ESM.pdf]

nature microbiology

Article https://doi.org/10.1038/s41564-023-01341-x

Cytoplasmic contractile injection systems mediate cell death in *Streptomyces*

In the format provided by the authors and unedited

Supplementary Table 1. Proteins detected by mass spectrometry in samples of purified CIS from WT *S. coelicolor* (ScoWT) and *S. venezuelae* (SvenWT), the corresponding Δ*cis2* mutants and the *S. coelicolor* non-contractile CIS^{Sc} mutant CIS−N5. Experiments were performed in biological replicates.

Protein ID	CIS ID	*S. coelicolor* WT	*S. coelicolor* Δ*cis2*	*S. coelicolor* CIS-N5	*S. venezuelae* WT	*S. venezuelae* Δ*cis2*
Sco4244/Vnz_28875	Cis11	-	-	29% coverage / 10 total unique peptide	-	-
Sco4245/Vnz_28880	Cis9	-	-	21% coverage / 2 total unique peptide	-	-
Vnz_28885	Cis10	-	-	-	-	-
Sco4246/Vnz_28890	Cis8	-	-	46% coverage / 18 total unique peptide	-	-
Sco4247/Vnz_28895	Cis7	-	-	51% coverage / 8 total unique peptide	-	-
Sco4248/Vnz_28900	Cis5	-	-	39% coverage / 4 total unique peptide	-	-
Sco4251/Vnz_28915	-	-	-	-	-	-
Sco4252/Vnz_28920	Cis1	27% coverage / 4 total unique peptide	-	32% coverage / 5 total unique peptide	17% coverage / 3 total unique peptide	-
Sco4253/Vnz_28925	Cis2	48% coverage / 26 total unique peptide	-	51% coverage / 28 total unique peptide	38% coverage / 17 total unique peptide	-
Sco4254	-	-	-	10% coverage / 6 total unique peptide	-	-
Sco4259/Vnz_28930	Cis15	-	-	-	-	-
Sco4260/Vnz_28935	Cis16	-	-	24% coverage / 4 total unique peptide	-	-

Supplementary Table 2. Cryo-EM data statistical analysis

	Contracted sheath shell	Extended sheath-tube module
**Data collection and processing**		
Nominal magnification	81,000	
Voltage (kV)	300	
Electron exposure (e^-/Å)	60 e^-/dose weighting (K2 Summit camera)	
Defocus range (µm)	1.5-3.5	
Pixel size (Å/pixel)	1.4	
Symmetry imposed	C6 + helical (twist = 26.58˚, rise = 17.22 Å)	C6 + helical (twist = 38.50˚, rise = 23.10 Å)
Final particles (No.)	4838	18822
Map resolution	3.6	3.9
FSC threshold	0.143	0.143
**Refinement**		
Map sharpening B factor (Å^2)	-118	-130

Model composition	(Four layers)	(Four layers)
Non-hydrogen atoms	68280	96384
Protein residues	8808	12480
Chains	24	48
R.M.S deviations		
Bond length (Å)	0.004	0.018
Bond angles (˚)	0.983	1.818
Validation		
MolProbity score	1.88	1.85
Clashscore	9.74	8.19
Rotamer outlier (%)	0.00	0.50
Ramachandran plot		
Favored (%)	94.61	93.89
Allowed (%)	5.39	5.62
Outlier (%)	0.00	0.50
Masked CC	0.85	0.68

Supplementary Table 3. Experimental approaches to study effects of CIS^{Sc} on interspecies competition.

**Target organisms**	**Functional assay**	**Procedures**
Saccharomyces cerevisiae<br>*Escherichia coli*<br>*Bacillus subtilis*<br>*Micrococcus luteus*<br>*S. venezuelae*	Killing assay on plate or in liquid	Co-incubation with *S. coelicolor* wild-type, ΔCIS^{Sc} and CIS-N5 mutant strains
		Co-incubation with purified CIS^{Sc} particles
Lactococcus lactis (Nisin producer)	Killing assay on plate	Co-incubation with nisin-treated *S. coelicolor* wild-type, ΔCIS^{Sc} and CIS-N5 mutant strains
Wax moth larvae	Injection into larvae hemocoel	Injection of purified CIS^{Sc} particles from *S. coelicolor* wild-type, ΔCIS^{Sc} and CIS-N5 mutant strains

Supplementary Table 4. Shown is a list of CIS-positive (presence of a CIS gene cluster in the genome indicated by "+") and CIS-negative (absence of a CIS gene cluster in the genome indicated by "-") *Streptomyces* strains based on Chen et al.[8]. All genomes were searched by BLAST for homologues of the *S. coelicolor* effectors (Sco4256-58; "+" indicates at least one homologue was present, "-" indicates no homologue was present) and of the putative cell-envelope adaptor (Sco4242; "+", present; "-", absent).

Organism Name – CIS-positive strains	Effectors (Sco4256-58)	Adaptor (Sco4242)
Streptomyces albireticuli MDJK11	-	+
Streptomyces alboflavus MDJK44	+	+
Streptomyces albulus NK660	-	-
Streptomyces albulus ZPM	-	-
Streptomyces albus DSM 41398	+	+
Streptomyces albus SM254	+	+
Streptomyces albus BK£-25	+	+
Streptomyces albus J1074	+	+
Streptomyces alfalfae ACCC40021	-	+
Streptomyces ambofaciens DSM 40697	+	+
Streptomyces ambofaciens ATCC 23877	+	+
Streptomyces anulatus ATCC 11523	+	+
Streptomyces autolyticus CGMCC0516	-	+

Streptomyces avermitilis MA-4680 = NBRC 14893	-	+
Streptomyces bingchenggensis BCW-1	-	+
Streptomyces cattleya NRRL 8057 = DSM 46488	-	+
Streptomyces cattleya NRRL 8057 = DSM 46488	-	+
Streptomyces chartreusis NRRL 3882	-	-
Streptomyces clavuligerus F613-1	+	+
Streptomyces clavuligerus ATCC 27064	+	+
Streptomyces coelicolor A3(2)	+	+
Streptomyces collinus Tu 365	-	+
Streptomyces cyaneogriseus subsp. Noncyanogenus NMWT 1	-	+
Streptomyces davaonensis JCM 4913	-	-
Streptomyces formicae KY5	+	-
Streptomyces fulvissimus DSM 40593	+	+
Streptomyces glaucescens GLA.O	-	+
Streptomyces globisporus TFH56	+	+
Streptomyces globisporus C-1027	+	+
Streptomyces griseochromogenes ATCC 14511	-	-
Streptomyces griseus subsp. griseus NBRC 13350	+	+
Streptomyces hygroscopicus XM201	-	+

Streptomyces hygroscopicus subsp. jinggangensis 5008	-	+
Streptomyces hygroscopicus subsp. jinggangensis TL01	-	+
Streptomyces hygroscopicus subsp. Limoneus KCTC 1717	-	+
Streptomyces incarnatus NRRL 8089	-	-
Streptomyces laurentii ATCC 31255	-	+
Streptomyces lavendulae subsp. Lavendulae CCM 3239	-	+
Streptomyces leeuwenhoekii C34	-	+
Streptomyces lividans 1326	+	+
Streptomyces lividans TK24	+	+
Streptomyces lunaelactis MM109	-	-
Streptomyces lydicus A02	-	+
Streptomyces lydicus 103	-	+
Streptomyces malaysiensis DSM 4137	-	+
Streptomyces niveus SCSIO 3406	-	+
Streptomyces niveus NCIMB 11891	-	+
Streptomyces nodosus ATCC 14899	-	+
Streptomyces noursei ATCC 11455	-	-
Streptomyces pactum KLBMP 5084	+	+
Streptomyces pactum ACT12	+	+

Streptomyces parvulus 2297	+	+
Streptomyces peucetius subsp. caesius ATCC 27952	-	+
Streptomyces pratensis ATCC 33331	+	-
Streptomyces pristinaespiralis HCCB 10218	-	-
Streptomyces pristinaespiralis ATCC 25486	-	-
Streptomyces rapamycinicus NRRL 5491	-	+
Streptomyces reticuli TUE45	-	-
Streptomyces roseochromogenus subsp. oscitans DS 12.976	-	-
Streptomyces rubrolavendulae MJM4426	-	-
Streptomyces scabiei 87.22	+	-
Streptomyces sp. 2114.2	+	-
Streptomyces sp. 3214.6	+	+
Streptomyces sp. 4F	+	+
Streptomyces sp. 769	-	+
Streptomyces sp. CCM_MD2014	+	+
Streptomyces sp. CFMR 7	+	+
Streptomyces sp. CLI2509	+	+
Streptomyces sp. fd1-xmd	+	-
Streptomyces sp. FR-008	+	+

Streptomyces sp. GBA 94-10	+	+
Streptomyces sp. M56	-	+
Streptomyces sp. NEAU-S7GS2	+	+
Streptomyces sp. PAMC 26508	+	+
Streptomyces sp. PBH53	-	+
Streptomyces sp. PVA 94-07	+	+
Streptomyces sp. S8	+	+
Streptomyces sp. SAT1	+	+
Streptomyces sp. Sge12	-	+
Streptomyces sp. SirexAA-E	-	-
Streptomyces sp. SM17	+	+
Streptomyces sp. SM18	-	+
Streptomyces sp. TLI_053	-	+
Streptomyces sp. TN58	-	+
Streptomyces sp. Tu6071	+	+
Streptomyces sp. Tue 6075	+	+
Streptomyces sp. WAC00288	+	-
Streptomyces venezuelae NRRL B-65442	+	+
Streptomyces venezuelae ATCC 15439	+	+

Streptomyces venezuelae ATCC 15439 chr I	+	+
Streptomyces venezuelae ATCC 10712	+	+
Streptomyces vietnamensis GIM4.0001	-	+
Streptomyces violaceoruber S21	+	+
Streptomyces violaceusniger Tu 4113	-	+
Streptomyces xiamenensis 318	+	-

Organism Name – CIS-negative strains	Effectors (Sco4256-58)	Adaptor (Sco4242)
Streptomyces actuosus ATCC 25421	+[*1]	+[*2]
Streptomyces antibioticus DSM 41481	-	+[*2]
Streptomyces gilvosporeus F607	-	-
Streptomyces lincolnensis NRRL 2936	-	-
Streptomyces pluripotens MUSC 135	-	+[*2]
Streptomyces pluripotens MUSC 137	-	-
Streptomyces puniciscabiei TW1S1	+[*1]	-
Streptomyces sp. 2323.1	-	-
Streptomyces sp. 452	-	-
Streptomyces sp. CdTB01	-	-

Streptomyces sp. CMB-StM0423	-	-
Streptomyces sp. CNQ-509	-	-
Streptomyces sp. HNM0039	-	-
Streptomyces sp. Mg1	-	-
Streptomyces sp. MOE7	-	-
Streptomyces sp. P3	-	-
Streptomyces sp. S10(2016)	-	-
Streptomyces sp. SCSIO 03032	-	-
Streptomyces sp. XZHG99	-	-
Streptomyces spongiicola HNM0071	-	-
Streptomyces sviceus ATCC 29083	-	-

[*1]: only N-terminal part detected (up to 270 amino acids)

[*2]: only C-terminal part detected (from 200 amino acids)

Supplementary Table 5: Bacterial strains, plasmids and cosmids used in this study.

Strain	Description	Construction	Source
Escherichia coli strains			
TOP10	*F⁻ mcrA Δ(mrr-hsdRMS-mcrBC) Φ80lacZΔM15 ΔlacX74 recA1 araD139 Δ(ara leu) 7697 galU galK rpsL (StrR) endA1 nupG*	Cloning	Invitrogen
ET12567/pUZ8002	*F⁻ dam13::Tn9 dcm6 hsdM hsdR recF143:: Tn10 galK2 galT22 ara-14 lacY1 xyl-5 leuB6 thi-1 tonA31 rpsL hisG4 tsx-78 mtl-1 glnV44*	ET12567 with helper plasmid pUZ8002	[1]
BW25113/pIJ790	*Δ(araD-araB)567 ΔlacZ4787(::rrnB-4) lacIp-4000(lacIQ), λ−rpoS369(Am) rph-1 Δ(rhaD-rhaB)568 hsdR514*	BW25113 containing λ RED recombination plasmid pIJ790	[2]
Rosetta (DE3)	*F^- ompT $hsdS_B(r_B^-\ m_B^-)$ gal dcm (DE3) pRARE (Cam^R)*	Host strain for protein overexpression	Merck
Streptomyces strains			
S. venezuelae NRRL B-65442	Wild Type (Sv-WT)		[3]
S. coelicolor M145	Wild Type (Sc-WT)		[4]

	SCP1$^-$ SCP2$^-$ derivative from *S. coelicolor* A3(2)		
SS381	*Sv-WT* Δ*vnz_28920::apr*	chromosomal *vnz_28920 (cis2)* locus deleted using pSS489	This study
SS383	*Sc-WT* Δ*sco4253::apr*	chromosomal *sco4253 (cis2)* locus deleted using pSS480	This study
SS387	*Sc-WT* Δ*sco4253-4251::apr*	chromosomal *sco4253-4251* locus deleted using pSS480	This study
SS389	*Sc-WT Δsco4253::apr attB Φ BT1 Sco4253-I274-ypet_Sco4252-51, hyg*^{R}	pSS501 integrated at ϕBT1 attachment site of SS383	This study
SS392	*Sc-WT* Δ*sco4253-51::apr attB ΦBT1 sco4253-N3-sco4252-51, hyg*^{R}	pSS503 integrated at ϕBT1 attachment site of SS387	This study
SS393	*Sc-WT* Δ*sco4253-51::apr attB ΦBT1 sco4253-N5-sco4252-51, hyg*^{R}	pSS504 integrated at ϕBT1 attachment site of SS387	This study
SS394	*Sc-WT* Δ*sco4253-51::apr attB ΦBT1 sco4253-N2-sco4252-51, hyg*^{R}	pSS505 integrated at ϕBT1 attachment site of SS387	This study
SS395	*Sc-WT* Δ*sco4253-51::apr attB ΦBT1 sco4253-51, hyg*^{R}	pSS500 integrated at ϕBT1 attachment site of SS387	This study

SS430	*Sc-WT ΦBT1 P_{ermE*}-sfgfp, hyg^R*	pSS150 integrated at ϕBT1 attachment site of Sc-WT	This study
SS431	*Sc-WT Δsco4253::apr attB Φ BT1 P_{ermE*}-sfgfp, hyg^R*	pSS150 integrated at ϕBT1 attachment site of SS383	This study
SS459	*Sc-WT Δsco4253-51::apr attB ΦBT1 sco4253-N5-sco4252-51, P_{ermE*}-sfgfp, hyg^R*	pSS610 integrated at ϕBT1 attachment site of SS387	This study
SS484	*Sc-WT ΦBT1 P_{cis2}-ypet, hyg^R*	pSS619 integrated at ϕBT1 attachment site of Sc-WT	This study
SS540	*Sc-WT Δsco4256-58::apr*	chromosomal *sco4256-58* locus deleted using pSS703	This study
SS550	*Sc-WT Δsco4256-58::apr attB Φ BT1 P_{ermE*}-sfgfp, hyg^R*	pSS150 integretated at the ϕBT1 attachment site of SS540	This study
Plasmids			
pIJ773	pBluescript KS (+) containing the apramycin resistance gene *apr* and *oriT* of plasmid RP4, flanked by FRT sites (Apr^R). Used as template for the amplification of the *apr-oriT* cassette for		[5]

	'REDIRECT' PCR targeting, Apr^R		
pIJ10257	Cloning vector for the conjugal transfer of DNA (under control of the *ermE** constitutive promoter). Integrates at the *ΦBT1* attachment site, Hyg^R		[6]
pIJ10770	Cloning vector for the conjugal transfer of DNA from *E. coli* to *Streptomyces* spp. Integrates at the *ΦBT1* attachment site, Hyg^R		[4]
pIJ10772	Modified pIJ10770, carries *mcherry* for construction of C-terminal fluorescent gene fusion. Integrates at the *ΦBT1* attachment site, Hyg^R		[4]
pUC19	*E. coli* multicopy cloning vector, $Carb^R$		[7]
pET21b	*E. coli* expression vector with C-terminal 6xHis tag, $Carb^R$		EMD Biosciences
pIJ12738	Derivative of pGM1190, an intermediate copy number, conjugative plasmid containing the temperature-	Used as intermediated cloning vector	[8]

	sensitive replication origin of pSG5, AprR		
pIJ10773	Modified pIJ10770, carries *ypet* for construction of C-terminal fluorescent gene fusion. Integrates at the *ΦBT1* attachment site, Hyg^R	Codon-optimised *ypet* was PCR *a*mplified with primer 34/4b followed by restriction digestion with XhoI/KpnI and ligation into pIJ10770 cut with XhoI/KpnI	This study
pSS150	pIJ10257 carrying P_{ermE*}-*sfgfp*, Hyg^R	Codon-optimised *sfgfp* was PCR *a*mplified with primer 268/269 followed by restriction digestion with NdeI/XhoI and ligation into pIJ10257 cut with NdeI/XhoI	This study
pSS480	Mutated cosmid StD-49 for REDIRECT containing Δ*sco4253::apr,* Km^R, $Carb^R$, Apr^R	The *sco4253* coding sequence on the cosmid vector StD-49 was replaced by an oriT-containing apramycin resistance cassette, which was amplified from pIJ773 using primer 1037/1038.	This study
pSS481	Mutated cosmid StD-49 for REDIRECT containing Δ*sco4253-4251::apr,* Km^R, $Carb^R$, Apr^R	The *sco4253-51* coding sequence on the cosmid vector StD-49 was replaced by an oriT-containing apramycin	This study

		resistance cassette, which was amplified from pIJ773 using primer 1037/1039.	
pSS489	Mutated cosmid Pl1-F14 for REDIRECT containing Δ*vnz28920::apr,* Km^R, $Carb^R$, Apr^R	The *vnz28920* coding sequence on the cosmid vector Pl1-F14 was replaced by an oriT-containing apramycin resistance cassette, which was amplified from pIJ773 using primer 1048/1049	This study
pSS494	pIJ12738 carrying *sco4253::21IE* (CIS^{Sc}-N2), Apr^R	Insertion of "IE" at amino acid position 21 in Sco4253. Plasmid was generated via Gibson Assembly from PCR fragments generated using genomic DNA and primer 1057/1058 and 1059/1060 and pIJ12738 cut with HindIII	This study
pSS495	pIJ12738 carrying *sco4253::21IEG* (CIS^{Sc}-N3), Apr^R	Insertion of "IEG" at amino acid position 21 in Sco4253 Plasmid was generated via Gibson Assembly from PCR fragments generated using genomic DNA primer 1061/1057 and	This study

		1059/1060 and pIJ12738 cut with HindIII	
pSS496	pIJ12738 carrying *sco4253::21IEGVG* (CIS^{Sc}-N5), Carb^{R}	Insertion of " IEGVG " at amino acid position 21 in Sco4253. Plasmid was generated via Gibson Assembly from PCR fragments generated using genomic DNA primer 1057/1062 and 1063/1060 and pIJ12738 cut with HindIII	This study
pSS497	pUC19 carrying *sco4253-51*, Carb^{R}	Amplification of *sco4253-4251* from genomic DNA with primer 1091/1092 followed by Gibson Assembly into pUC19 cut with HindIII/EcoRI	This study
pSS498	pSS497 carrying *sco4253::ypet(I274)-Sco4252-51,* Carb^{R}	Insertion of *ypet* with linker after AA I274 in *sco4253*. pSS497 was amplified with primer 1075/1078, *ypet with 7AA linker* was amplified with primer 1076/1077, both fragments were combined using Gibson Assembly	This study
pSS500	pIJ10770 carrying *sco4253-51*, Hyg^{R}	Sco4253-4251 was PCR amplified with primer	This study

		1042/1101, digested with HindIII/NdeI and ligated into pIJ10770 cut with HindIII/NdeI	
pSS501	*pIJ10770* carrying *sco4253::ypet(I274)-sco4252-51,* Hyg^R	*sco4253::ypet(I274)-Sco4252-51* was PCR amplified with primers 1042/1101 from pSS498, digested with HindIII/NdeI and ligated into pIJ10770 cut with HindIII/NdeI	This study
pSS503	*pIJ10770* carrying CIS^{Sc}*-N3 (sco4253::21IEG-sco4252-51),* Hyg^R	Fragment 1: *sco4253-N3-4251* from pSS495 was PCR amplified with primer 1042/1043 and digested with HindIII/NruI; Fragment 2: *sco4251-53* was PCR amplified from pSS494 with primer 1042/1102 and digested with NruI/AvrII; Triple ligation of both fragments with pIJ10770 cut with HindIII/AvrII	This study
pSS504	*pIJ10770* carrying CIS^{Sc}*-N5 (sco4253::21IEGVG-sco4252-51),* Hyg^R	fragment 1: *sco4253-N5-4251* from pSS496 was PCR amplified with primer 1042/1043 and digested with	This study

		HindIII/NruI; Fragment 2: *sco4251-53* was PCR amplified from pSS494 with primer 1042/1102 and digested with NruI/AvrII; Triple ligation of both fragments with pIJ10770 cut with HindIII/AvrII	
pSS505	*pIJ10770* carrying *CIS*^{Sc}*-N2 (sco4253::21IE-sco4252-51),* Hyg^R	Fragment 1: *sco4253-N2-4251* from pSS494 was PCR amplified with primer 1042/1043 and cut with HindIII/NruI; Fragment 2: *sco4251-53* was PCR amplified from pSS494 with primer 1042/1102 and cut with NruI/AvrII; Triple ligation of both fragments into pIJ10770 cut with HindIII/AvrII	This study
pSS610	pSS504 carrying P_{ermE*}*-sfgfp, Hyg*^{R}	P_{ermE*}*-sfgfp* fragment was isolated from pSS150 by restriction digestion with Bsu361/AvrII and ligated between the Bsu361/AvrII site of pSS504	This study

pSS619	pIJ10773 carrying *P_{str2}-ypet,* Hyg^R	*sco4253* promoter region (*P_{str2}*) was PCR amplified from genomic DNA with primer 1403/1404 and cloned into pIJ10773 cut with NdeI/XhoI using Gibson Assembly	This study
pSS703	Mutated cosmid StD8A for REDIRECT containing Δ*sco4256-4258::apr,* Km^R, Carb^R, Apr^R	The *sco4256-58* coding sequence on the cosmid vector StD8A was replaced by an oriT-containing apramycin resistance cassette, which was amplified from pIJ773 using primer 1713/1717.	This study
pSS704	pET21b carrying *FLAG-yfp-gypA,* Carb^R	*FLAG-yfp* with synthetic membrane anchor *gypA* was amplified with primer 1736/1737 and inserted into pET21b by restriction ligation using NdeI and HindIII.	This study
pSS709	pET21b carrying *sco4256,* Carb^R	*sco4256* was PCR amplified with primer 1738/42, pET21b was amplified with primer 1743/1744. Fragments were assembled using Gibson Assembly.	This study

pSS712	pET21b carrying *sco4257,* Carb^{R}	*sco4257* was PCR amplified with primer 1746/50, pET21b was amplified with primer 1743/1744. Fragments were assembled using Gibson Assembly.	This study
pSS716	pET21b carrying *sco4258,* Carb^{R}	*sco4258* was PCR amplified with primer 1751/55, pET21b was amplified with primer 1743/1744. Fragments were assembled using Gibson Assembly.	This study
Cosmids			
StD-49 and StD8A	Cosmid vector containing coding sequence for *S. coelicolor CIS gene cluster*, Km^{R}, Carb^{R}		http://strepdb. streptomyces.org.uk
Pl1-F14	Cosmid vector containing coding sequence for *S. venezuelae CIS gene cluster*, Km^{R}, Carb^{R}		http://strepdb. streptomyces.org.uk

Supplementary Table 5 References

1. Paget, M. S. B., Chamberlin, L., Atrih, A., Foster, S. J. & Buttner, M. J. Evidence that the Extracytoplasmic Function Sigma Factor ςE Is Required for Normal Cell Wall Structure in Streptomyces coelicolor A3(2). *J. Bacteriol.* **181**, 204–211 (1999).
2. Datsenko, K. A. & Wanner, B. L. One-step inactivation of chromosomal genes in Escherichia coli K-12 using PCR products. *Proc. Natl. Acad. Sci.* **97**, 6640–6645 (2000).
3. Gomez-Escribano, J. P. *et al.* Streptomyces venezuelae NRRL B-65442: genome sequence of a model strain used to study morphological differentiation in filamentous actinobacteria. *J. Ind. Microbiol. Biotechnol.* **48**, kuab035 (2021).
4. T. Kieser M. J. Bibb M. J. Buttner K. F. Chater and D. A. Hopwood. *Practical Streptomyces Genetics*. (John Innes Foundation, 2000).
5. Gust, B., Challis, G. L., Fowler, K., Kieser, T. & Chater, K. F. PCR-targeted Streptomyces gene replacement identifies a protein domain needed for biosynthesis of the sesquiterpene soil odor geosmin. *Proc. Natl. Acad. Sci. U. S. A.* **100**, 1541–1546 (2003).
6. Hong, H.-J., Hutchings, M. I., Hill, L. M. & Buttner, M. J. The role of the novel Fem protein VanK in vancomycin resistance in Streptomyces coelicolor. *J. Biol. Chem.* **280**, 13055–13061 (2005).
7. Yanisch-Perron, C., Vieira, J. & Messing, J. Improved M13 phage cloning vectors and host strains: nucleotide sequences of the M13mp18 and pUC19 vectors. *Gene* **33**, 103–119 (1985).
8. Fernández-Martínez, L. T. & Bibb, M. J. Use of the meganuclease I-SceI of Saccharomyces cerevisiae to select for gene deletions in actinomycetes. *Sci. Rep.* **4**, 7100 (2014).

Supplementary Table 6: Oligonucleotides used in this study.

Name	Sequence (5' –> 3')
4b	GGGGTACCTCACTTGTACAGCTCGTTCATG
34	ATACTCGAGATGGTCTCCAAGGGCGAGG
268	TTAATTAACATATGTCCCGCCTGAAGCCGCGCTGCACCTCCCTGGAGATGTCCAAGGGCGAGGAGCTGTTC
269	ATATACTCGAGCTCCGGGCCCGGCAGCTCCGGGCCCGGCAGCTTGTACAGCTCGTCCATGCCGTG
1037	GAGCAGAGCATGCCGTCCTACCTGTCGCCCGGCGTCTACATTCCGGGGATCCGTCGACC
1038	GCGATCCGCCTACTCGTCCAGTTCGCCGCCGCCGCTGGATGTAGGCTGGAGCTGCTTC
1039	TCGCGTACGTCACGATTCCCCCAGGCGGCTCCCGCCGAGTGTAGGCTGGAGCTGCTTC
1042	ATTAAAGCTTCCCTCCTGACACGCCGTCACC
1043	AATTAATTCATATGCTCGTCCAGTTCGCCGCCGC
1048	GGAGCGAGCATGCCGACGTACCTCACCCCGGGCGTGTACATTCCGGGGATCCGTCGACC
1049	ACGTACGCAGTTCACGCCGATCGGGTTGAGCAGGTCCTGTGTAGGCTGGAGCTGCTTC
1050	CGGCGTCCGTCAGCCGCGCTGGAAGAACTCCACCTCCGCTGTAGGCTGGAGCTGCTTC
1057	CACGACGTTGTAAAACGACGGCCAGTGCCAAGTGTCGTGCGCCGTCCCGTGGTC
1058	GGCCGCCACCGACGTGCCCACTCCCTCGATCTCGATCGGGCGCGAGCCGCTGGCCA
1059	ATCGAGGGAGTGGGCACGTC
1060	GCGGATCCTCTAGAGTCGACCTGCAGCCCAAGTTCTTCGAACACGATGGTGATGG
1061	GGCCGCCACCGACGTGCCCACTCCCTCGATGCCCTCGATCGGGCGCGAGCCGCTGGCCA
1062	GCCCACTCCCTCGATGCCGACGCCCTCGATCGGGCGCGAGCCGCTGGCCA
1063	ATCGAGGGCGTCGGCATCGAGGGAGTGGGCACGTC

1075	CCGAGCCTTCGAGGATCGCGCCGCGCTGGTAGG
1076	CGCGGCGCGATCCTCGAAGGCTCGGGGCAGGGG
1077	GGCCTCCAGGTCGCTCCCCTGGCCGGAGCCCG
1078	CGGCCAGGGGAGCGACCTGGAGGCCGTCAAAGC
1091	TGACCATGATTACGCCAAGCTTCCCTCCTGACACGCCGTCAC
1092	AAACGACGGCCAGTGAATTCCGCGATCTCCTCGTGCAGCC
1101	AATTAATTCATATGCGCGATCTCCTCGTGCAGCC
1403	TGATAAGTTTATCAAGCTTAGATTCTCTCATATGGTTCAAGCGGTCCGACACG
1404	GTGAACAGCTCCTCGCCCTTGGAGACCATCTCGAGCTCTCCTCGGGGTACGAGACAG
1713	TCTGCCCCGCCAGGTCCTGACGAAGTGCCGGACCGGCCGATTCCGGGGATCCGTCGACC
1717	GGCTGGTGACGGCCGCCGGGCCCCCTCCGGCCGTCAGCCTGTAGGCTGGAGCTGCTTC
1736	ATTAATTCATATGGACTACAAGGACGACGACGAC
1737	ATTAAAGCTTGCAGATCAGGCGGCGGATG
1743	AAGCTTGCGGCCGCACTCG
1744	ATGTATATCTCCTTCTTAAAGTTAAACAAAATTATTTCTAGAGGGGA
1746	TTTGTTTAACTTTAAGAAGGAGATATACATATGAGCCTTTGGACCTCCC
1750	GTGGTGGTGCTCGAGTGCGGCCGCAAGCTTGTCCTTCGACGGGTG
1751	TTTGTTTAACTTTAAGAAGGAGATATACATATGAGCCTGTGGACTTCCC
1755	GTGGTGGTGCTCGAGTGCGGCCGCAAGCTTGCCCTTCTTCGGGTG

56
