# Supplementary material for: Cytoplasmic contractile injection systems mediate cell death in Streptomyces
Source: Nat Microbiol. 2023 Mar 9;8(4):711–26. doi: 10.1038/s41564-023-01341-x (PMC10066040; doi:10.1038/s41564-023-01341-x)
Supplement: Supplementary file 2 — Reporting Summary [file 41564_2023_1341_MOESM2_ESM.pdf]

nature portfolio

Corresponding author(s): Pilhofer/Schlimpert

Last updated by author(s): Jan 26, 2023

Reporting Summary

Nature Portfolio wishes to improve the reproducibility of the work that we publish. This form provides structure for consistency and transparency in reporting. For further information on Nature Portfolio policies, see our Editorial Policies and the Editorial Policy Checklist.

Statistics

For all statistical analyses, confirm that the following items are present in the figure legend, table legend, main text, or Methods section.

n/a	Confirmed	
☐	☒	The exact sample size (*n*) for each experimental group/condition, given as a discrete number and unit of measurement
☐	☒	A statement on whether measurements were taken from distinct samples or whether the same sample was measured repeatedly
☐	☒	The statistical test(s) used AND whether they are one- or two-sided<br>*Only common tests should be described solely by name; describe more complex techniques in the Methods section.*
☒	☐	A description of all covariates tested
☒	☐	A description of any assumptions or corrections, such as tests of normality and adjustment for multiple comparisons
☐	☒	A full description of the statistical parameters including central tendency (e.g. means) or other basic estimates (e.g. regression coefficient) AND variation (e.g. standard deviation) or associated estimates of uncertainty (e.g. confidence intervals)
☐	☒	For null hypothesis testing, the test statistic (e.g. *F*, *t*, *r*) with confidence intervals, effect sizes, degrees of freedom and *P* value noted<br>*Give P values as exact values whenever suitable.*
☒	☐	For Bayesian analysis, information on the choice of priors and Markov chain Monte Carlo settings
☒	☐	For hierarchical and complex designs, identification of the appropriate level for tests and full reporting of outcomes
☒	☐	Estimates of effect sizes (e.g. Cohen's *d*, Pearson's *r*), indicating how they were calculated

Our web collection on statistics for biologists contains articles on many of the points above.

Software and code

Policy information about availability of computer code

Data collection	SPA cryoEM and cryoET data collection: SerialEM-3.8<br>cryoFIB milling: Thermo Fisher Scientific XT software<br>Light microscopy: Zen-3.5 and , LasX-3.7.4.23463<br>Automated Western Blot analysis: Compass for Simple Western, Version 6.0.0
Data analysis	MotionCor2_1.1.0, Gctf-v1.06_sm_30, Relion-3.0, UCSF Chimera-1.13, UCSF ChimeraX-1.1, PyMOL-2.3.2, COOT-0.8.9.1, PHENIX-1.13-2998 (including phenix.real_space_refine, phenix.molprobity), ImageJ-1.53f51(Fiji), IMOD-4.11.0, Rosetta-2018.09.60072, MEGAX-10.1.1, GraphPad Prism-9.3.1, tom_deconv, MUSCLE, Scaffold-4.11.1, Compass-6.0.0

For manuscripts utilizing custom algorithms or software that are central to the research but not yet described in published literature, software must be made available to editors and reviewers. We strongly encourage code deposition in a community repository (e.g. GitHub). See the Nature Portfolio guidelines for submitting code & software for further information.

Data

Policy information about availability of data

All manuscripts must include a data availability statement. This statement should provide the following information, where applicable:

- Accession codes, unique identifiers, or web links for publicly available datasets
- A description of any restrictions on data availability
- For clinical datasets or third party data, please ensure that the statement adheres to our policy

Representative reconstructed tomograms (EMD-16200, EMD-16201, EMD-16202, EMD-16203, EMD-16204, EMD-16205, EMD-16206, EMD-16208 and EMD-16210) and SPA cryoEM maps (EMD-16098 and EMD-16101) have been deposited in the Electron Microscopy Data Bank. Atomic models (PDB: 8BKY and PDB: 8BL4) have been deposited in the Protein Data Bank.

Human research participants

Policy information about studies involving human research participants and Sex and Gender in Research.

Reporting on sex and gender	NA
Population characteristics	NA
Recruitment	NA
Ethics oversight	NA

Note that full information on the approval of the study protocol must also be provided in the manuscript.

Field-specific reporting

Please select the one below that is the best fit for your research. If you are not sure, read the appropriate sections before making your selection.

☒ Life sciences ☐ Behavioural & social sciences ☐ Ecological, evolutionary & environmental sciences

For a reference copy of the document with all sections, see nature.com/documents/nr-reporting-summary-flat.pdf

Life sciences study design

All studies must disclose on these points even when the disclosure is negative.

Sample size	Sample size was stated in the text for each of our functional assays. Imaging experiments were performed on samples derived from at least 2 independent cultures (as stated in the figure legends).
Data exclusions	No data was excluded from the analyses preformed.
Replication	All data (WB, TEM, cryoET, FLM, CLEM) was confirmed with proper biological replicates (from two to five independent times, as stated in the figure legends) to ensure reproducibility of the assays shown in the study, with all attempts at replication being successful.
Randomization	Extracted particles (for SPA) were randomly assigned to two separate groups to calculate half-maps and gold standard FSC.<br>For other experiments, no randomization was performed as it does not involve participant groups.
Blinding	Blinding was not attempted to our study, as it is laboratory cultivation based and the researchers involved need to verify samples and controls for each experiment.

Reporting for specific materials, systems and methods

We require information from authors about some types of materials, experimental systems and methods used in many studies. Here, indicate whether each material, system or method listed is relevant to your study. If you are not sure if a list item applies to your research, read the appropriate section before selecting a response.

Materials & experimental systems

n/a	Involved in the study
	☒ Antibodies
☒	Eukaryotic cell lines
☒	Palaeontology and archaeology
	☒ Animals and other organisms
☒	Clinical data
☒	Dual use research of concern

Methods

n/a	Involved in the study
☒	ChIP-seq
☒	Flow cytometry
☒	MRI-based neuroimaging

Antibodies

Antibodies used

Rabbit polyclonal anti-Cis1a (inner tube protein, generated from GenScript);
Rabbit polyclonal anti-Cis2 (sheath protein, generated from GenScript);
Rabbit polyclonal anti-WhiA (Cambridge Bioscience);
Anti-rabbit secondary antibody detection module, ProteinSimple DM-001

Validation

Validation of commercially available antibody was based on the technical data sheet provided by the manufacturer.
Custom-made antibodies anti-WhiA was validated before in Bush et al, mBio 4, e00684-13 (2013), respectively.
The qualities of rabbit polyclonal anti-Cis1a and anti-Cis2 were tested by ELISA and WB with the purified antigen proteins in quality control part from GenScript company.

Animals and other research organisms

Policy information about studies involving animals; ARRIVE guidelines recommended for reporting animal research, and Sex and Gender in Research

Laboratory animals	Wax moth larvae (1-3 weeks old)
Wild animals	No wild animals haven been used in this study.
Reporting on sex	This information has not been collected because it is not relevant for this study.
Field-collected samples	The study did not involve samples collected from the field.
Ethics oversight	No ethics approval was required.

Note that full information on the approval of the study protocol must also be provided in the manuscript.
