# Supplementary material for: Cytoplasmic contractile injection systems mediate cell death in Streptomyces
Source: Nat Microbiol. 2023 Mar 9;8(4):711–26. doi: 10.1038/s41564-023-01341-x (PMC10066040; doi:10.1038/s41564-023-01341-x)
Supplement: Supplementary file 6 — PDB validation report for 8BL4. [file 41564_2023_1341_MOESM6_ESM.zip › D_1292126605_val-report-full_P1(final).pdf]

Preliminary Full wwPDB EM Validation Report (i)

Nov 24, 2022 – 05:46 pm GMT

Deposition ID : D_1292126605

This wwPDB validation report is NOT for manuscript review

This is a Preliminary Full wwPDB EM Validation Report.

This report is produced by the wwPDB Deposition System during initial deposition but before annotation of the structure.

We welcome your comments at *validation@mail.wwpdb.org*
A user guide is available at
https://www.wwpdb.org/validation/2017/EMValidationReportHelp
with specific help available everywhere you see the (i) symbol.

The types of validation reports are described at
http://www.wwpdb.org/validation/2017/FAQs#types.

Not For Manuscript Review

The following versions of software and data (see references (i)) were used in the production of this report:

EMDB validation analysis	:	0.0.1.dev43
MolProbity	:	4.02b-467
Percentile statistics	:	20191225.v01 (using entries in the PDB archive December 25th 2019)
MapQ	:	1.9.9
Ideal geometry (proteins)	:	Engh & Huber (2001)
Ideal geometry (DNA, RNA)	:	Parkinson et al. (1996)
Validation Pipeline (wwPDB-VP)	:	2.31.3

1 Overall quality at a glance (i)

The following experimental techniques were used to determine the structure:
ELECTRON MICROSCOPY

The reported resolution of this entry is 3.90 Å.

Percentile scores (ranging between 0-100) for global validation metrics of the entry are shown in the following graphic. The table shows the number of entries on which the scores are based.

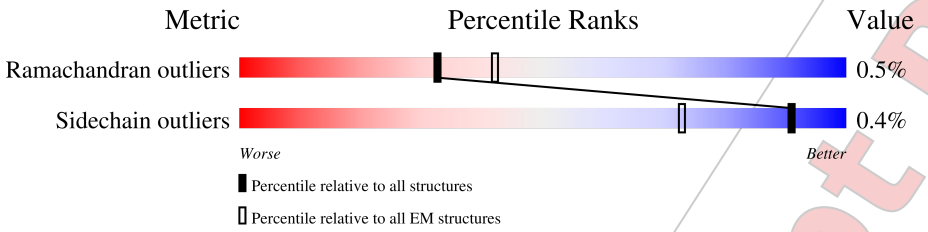

Metric	Whole archive (#Entries)	EM structures (#Entries)
Ramachandran outliers	154571	4023
Sidechain outliers	154315	3826

The table below summarises the geometric issues observed across the polymeric chains and their fit to the map. The red, orange, yellow and green segments of the bar indicate the fraction of residues that contain outliers for >=3, 2, 1 and 0 types of geometric quality criteria respectively. A grey segment represents the fraction of residues that are not modelled. The numeric value for each fraction is indicated below the corresponding segment, with a dot representing fractions <=5% The upper red bar (where present) indicates the fraction of residues that have poor fit to the EM map (all-atom inclusion < 40%). The numeric value is given above the bar.

Mol	Chain	Length	Quality of chain
1	A	539	5% 67% • 31%
1	B	539	5% 67% • 31%
1	C	539	• 67% • 31%
1	D	539	5% 67% • 31%
1	E	539	5% 67% • 31%
1	F	539	• 67% • 31%
1	G	539	5% 67% • 31%
1	H	539	6% 67% • 31%
1	I	539	5% 67% • 31%

Continued on next page...

Not For Manuscript Review

Continued from previous page...

Mol	Chain	Length	Quality of chain
1	J	539	5% / 67% / • / 31%
1	K	539	5% / 67% / • / 31%
1	L	539	5% / 67% / • / 31%
1	M	539	6% / 66% / • / 31%
1	N	539	7% / 66% / • / 31%
1	O	539	6% / 66% / • / 31%
1	P	539	6% / 66% / • / 31%
1	Q	539	7% / 66% / • / 31%
1	R	539	6% / 66% / • / 31%
1	S	539	9% / 67% / • / 31%
1	T	539	8% / 67% / • / 31%
1	U	539	8% / 66% / • / 31%
1	V	539	8% / 67% / • / 31%
1	W	539	8% / 67% / • / 31%
1	X	539	8% / 67% / • / 31%
2	a	149	7% / 97% / •
2	b	149	6% / 96% / •
2	c	149	7% / 97% / •
2	d	149	6% / 96% / •
2	e	149	7% / 97% / •
2	f	149	5% / 97% / •
2	g	149	5% / 99% / •
2	h	149	6% / 99% / •
2	i	149	5% / 99% / •
2	j	149	• / 99% / •

Continued on next page...

Not For Manuscript Review

Continued from previous page...

Mol	Chain	Length	Quality of chain
2	k	149	5% 99% .
2	l	149	5% 100%
2	m	149	7% 97% .
2	n	149	7% 97% .
2	o	149	7% 97% .
2	p	149	7% 97% .
2	q	149	7% 97% .
2	r	149	7% 97% .
2	s	149	8% 99% .
2	t	149	7% 99% .
2	u	149	8% 99% .
2	v	149	8% 99% .
2	w	149	7% 99% .
2	x	149	8% 99% .

Not For Manuscript Review

2 Entry composition (i)

There are 2 unique types of molecules in this entry. The entry contains 96384 atoms, of which 0 are hydrogens and 0 are deuteriums.

In the tables below, the AltConf column contains the number of residues with at least one atom in alternate conformation and the Trace column contains the number of residues modelled with at most 2 atoms.

- Molecule 1 is a protein called Putative phage tail sheath protein.

Mol	Chain	Residues	Atoms					AltConf	Trace
1	V	371	Total 2866	C 1818	N 503	O 537	S 8	0	0
1	P	371	Total 2866	C 1818	N 503	O 537	S 8	0	0
1	J	371	Total 2866	C 1818	N 503	O 537	S 8	0	0
1	D	371	Total 2866	C 1818	N 503	O 537	S 8	0	0
1	W	371	Total 2866	C 1818	N 503	O 537	S 8	0	0
1	Q	371	Total 2866	C 1818	N 503	O 537	S 8	0	0
1	K	371	Total 2866	C 1818	N 503	O 537	S 8	0	0
1	E	371	Total 2866	C 1818	N 503	O 537	S 8	0	0
1	X	371	Total 2866	C 1818	N 503	O 537	S 8	0	0
1	R	371	Total 2866	C 1818	N 503	O 537	S 8	0	0
1	L	371	Total 2866	C 1818	N 503	O 537	S 8	0	0
1	F	371	Total 2866	C 1818	N 503	O 537	S 8	0	0
1	S	371	Total 2866	C 1818	N 503	O 537	S 8	0	0
1	M	371	Total 2866	C 1818	N 503	O 537	S 8	0	0
1	G	371	Total 2866	C 1818	N 503	O 537	S 8	0	0
1	A	371	Total 2866	C 1818	N 503	O 537	S 8	0	0
1	T	371	Total 2866	C 1818	N 503	O 537	S 8	0	0

Continued on next page...

Not For Manuscript Review

Not For Manuscript Review

Continued from previous page...

Mol	Chain	Residues	Atoms					AltConf	Trace
1	N	371	Total 2866	C 1818	N 503	O 537	S 8	0	0
1	H	371	Total 2866	C 1818	N 503	O 537	S 8	0	0
1	B	371	Total 2866	C 1818	N 503	O 537	S 8	0	0
1	U	371	Total 2866	C 1818	N 503	O 537	S 8	0	0
1	O	371	Total 2866	C 1818	N 503	O 537	S 8	0	0
1	I	371	Total 2866	C 1818	N 503	O 537	S 8	0	0
1	C	371	Total 2866	C 1818	N 503	O 537	S 8	0	0

- Molecule 2 is a protein called Phage tail protein.

Mol	Chain	Residues	Atoms					AltConf	Trace
2	v	149	Total 1150	C 719	N 198	O 225	S 8	0	0
2	p	149	Total 1150	C 719	N 198	O 225	S 8	0	0
2	j	149	Total 1150	C 719	N 198	O 225	S 8	0	0
2	d	149	Total 1150	C 719	N 198	O 225	S 8	0	0
2	w	149	Total 1150	C 719	N 198	O 225	S 8	0	0
2	q	149	Total 1150	C 719	N 198	O 225	S 8	0	0
2	k	149	Total 1150	C 719	N 198	O 225	S 8	0	0
2	e	149	Total 1150	C 719	N 198	O 225	S 8	0	0
2	x	149	Total 1150	C 719	N 198	O 225	S 8	0	0
2	r	149	Total 1150	C 719	N 198	O 225	S 8	0	0
2	l	149	Total 1150	C 719	N 198	O 225	S 8	0	0
2	f	149	Total 1150	C 719	N 198	O 225	S 8	0	0

Continued on next page...

Continued from previous page...

Mol	Chain	Residues	Atoms					AltConf	Trace
2	s	149	Total 1150	C 719	N 198	O 225	S 8	0	0
2	m	149	Total 1150	C 719	N 198	O 225	S 8	0	0
2	g	149	Total 1150	C 719	N 198	O 225	S 8	0	0
2	a	149	Total 1150	C 719	N 198	O 225	S 8	0	0
2	t	149	Total 1150	C 719	N 198	O 225	S 8	0	0
2	n	149	Total 1150	C 719	N 198	O 225	S 8	0	0
2	h	149	Total 1150	C 719	N 198	O 225	S 8	0	0
2	b	149	Total 1150	C 719	N 198	O 225	S 8	0	0
2	u	149	Total 1150	C 719	N 198	O 225	S 8	0	0
2	o	149	Total 1150	C 719	N 198	O 225	S 8	0	0
2	i	149	Total 1150	C 719	N 198	O 225	S 8	0	0
2	c	149	Total 1150	C 719	N 198	O 225	S 8	0	0

Not For Manuscript Review

3 Residue-property plots ⓘ

These plots are drawn for all protein, RNA, DNA and oligosaccharide chains in the entry. The first graphic for a chain summarises the proportions of the various outlier classes displayed in the second graphic. The second graphic shows the sequence view annotated by issues in geometry and atom inclusion in map density. Residues are color-coded according to the number of geometric quality criteria for which they contain at least one outlier: green = 0, yellow = 1, orange = 2 and red = 3 or more. A red diamond above a residue indicates a poor fit to the EM map for this residue (all-atom inclusion < 40%). Stretches of 2 or more consecutive residues without any outlier are shown as a green connector. Residues present in the sample, but not in the model, are shown in grey.

• Molecule 1: Putative phage tail sheath protein

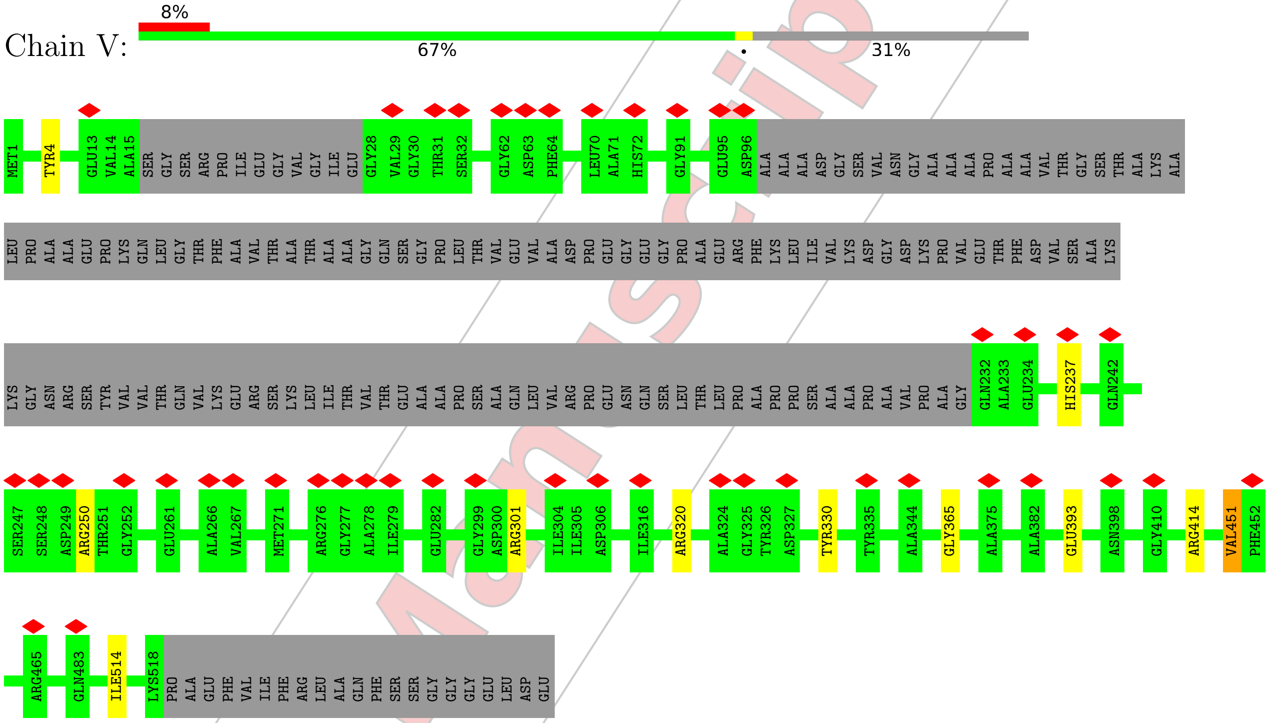

• Molecule 1: Putative phage tail sheath protein

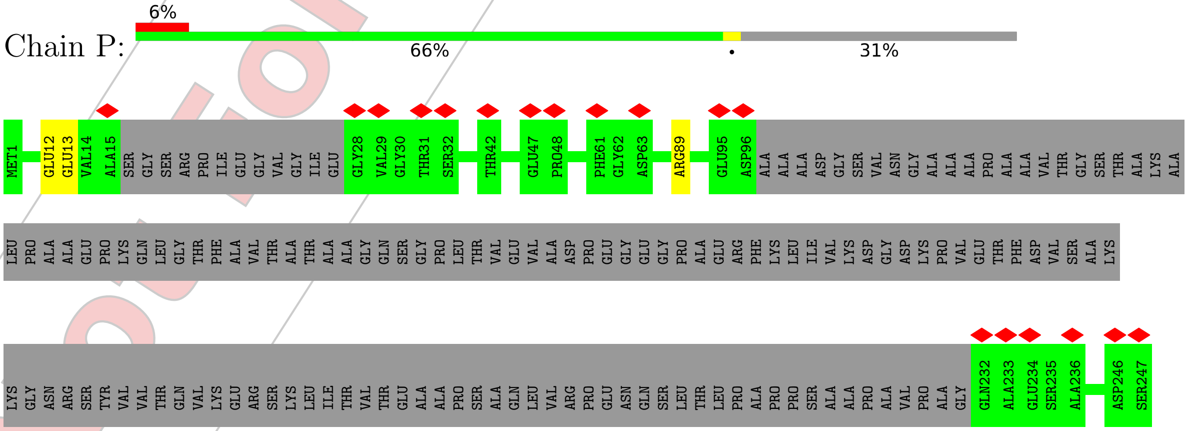

Not For Manuscript Review

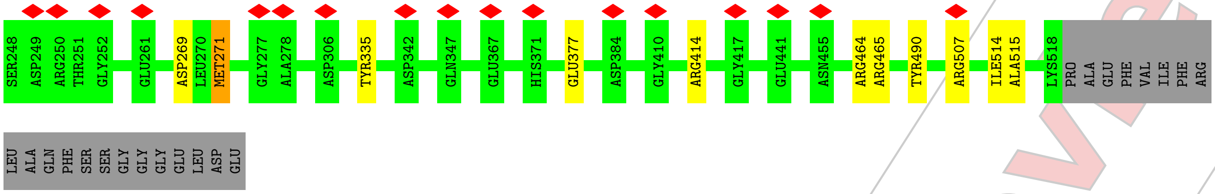

• Molecule 1: Putative phage tail sheath protein

Chain J:

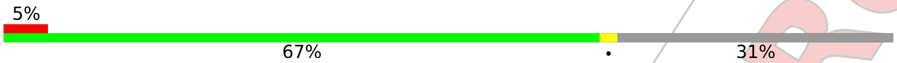

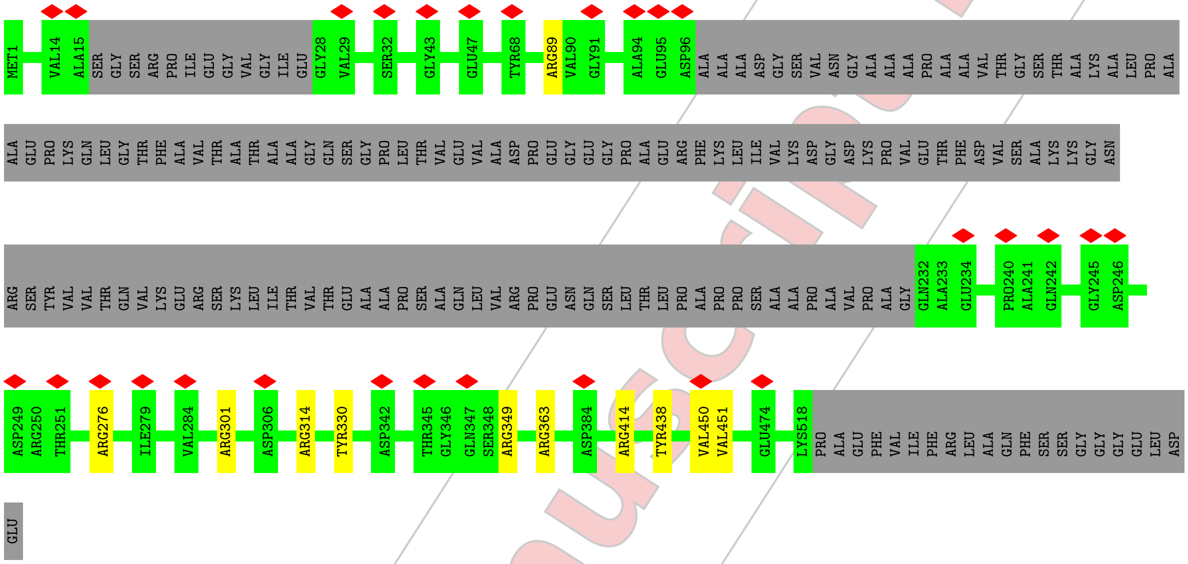

• Molecule 1: Putative phage tail sheath protein

Chain D:

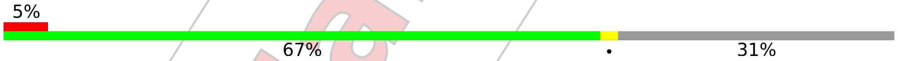

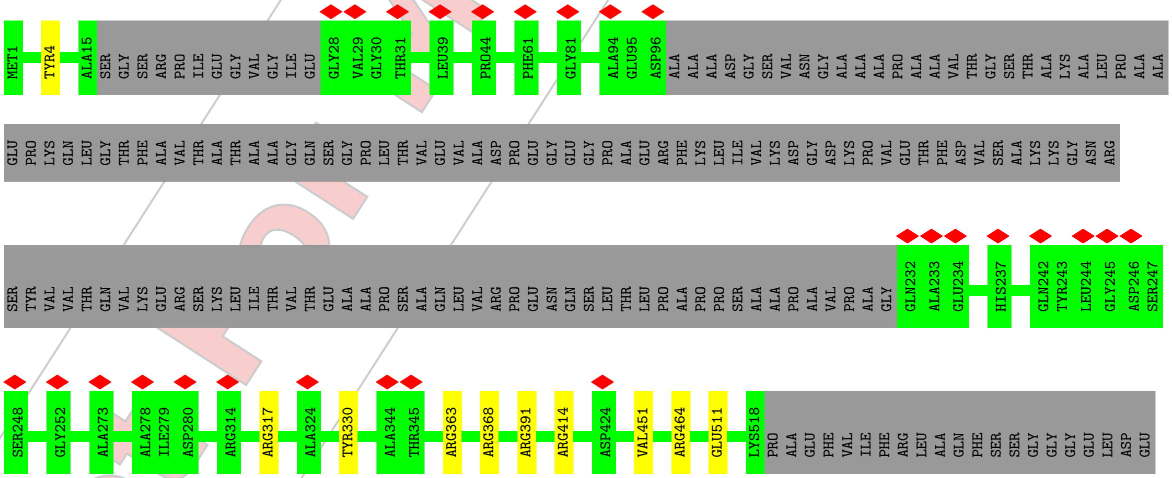

• Molecule 1: Putative phage tail sheath protein

Chain W:

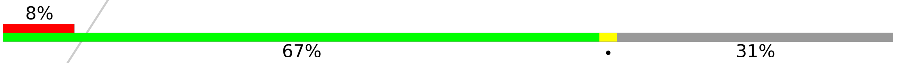

Not For Manuscript Review

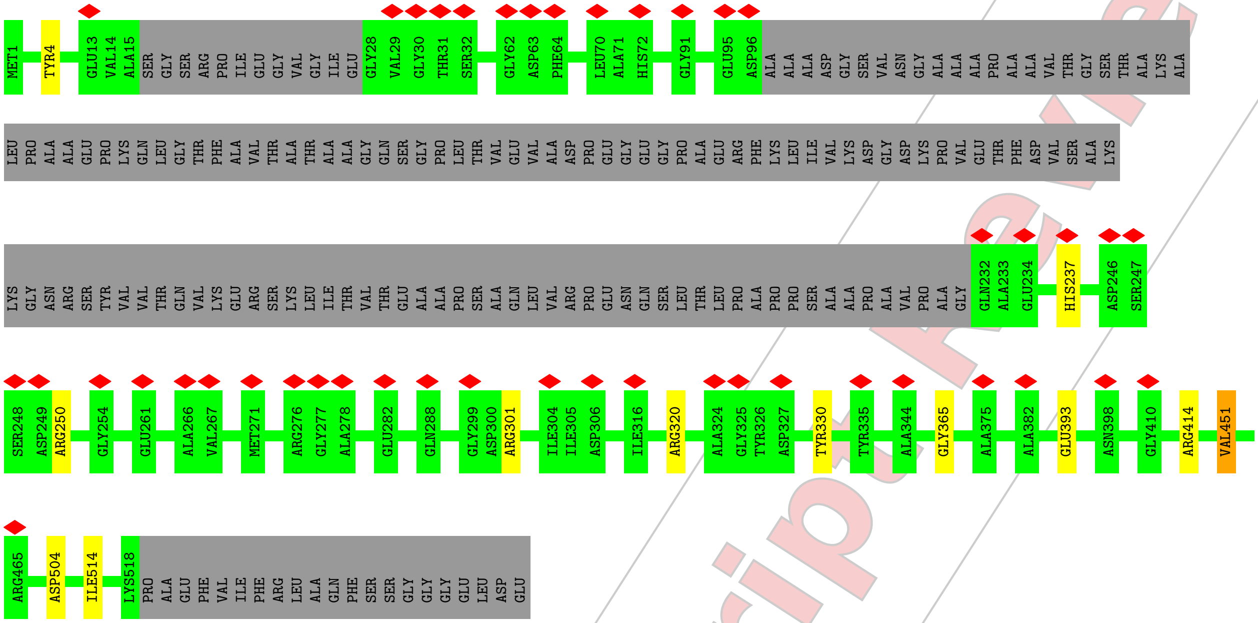

- Molecule 1: Putative phage tail sheath protein

Chain Q:

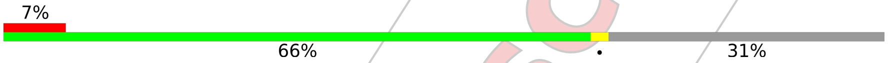

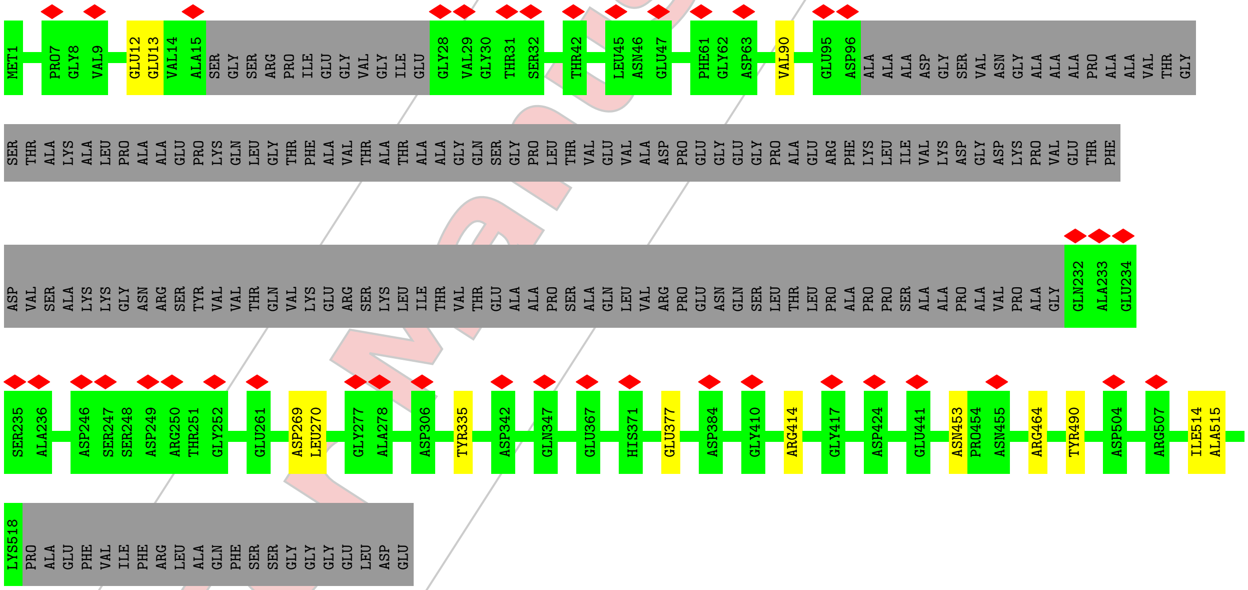

- Molecule 1: Putative phage tail sheath protein

Chain K:

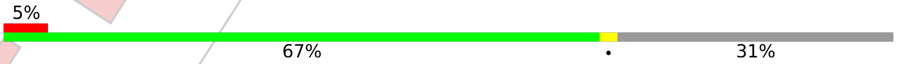

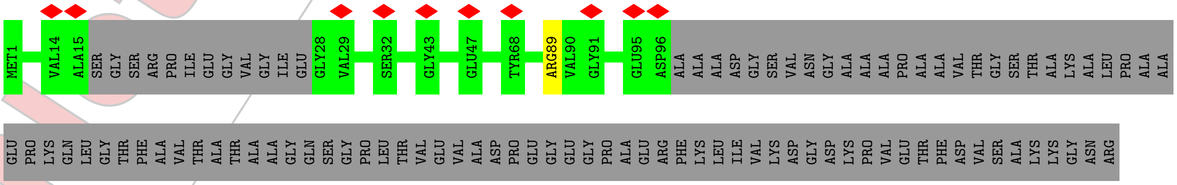

Not For Manuscript Review

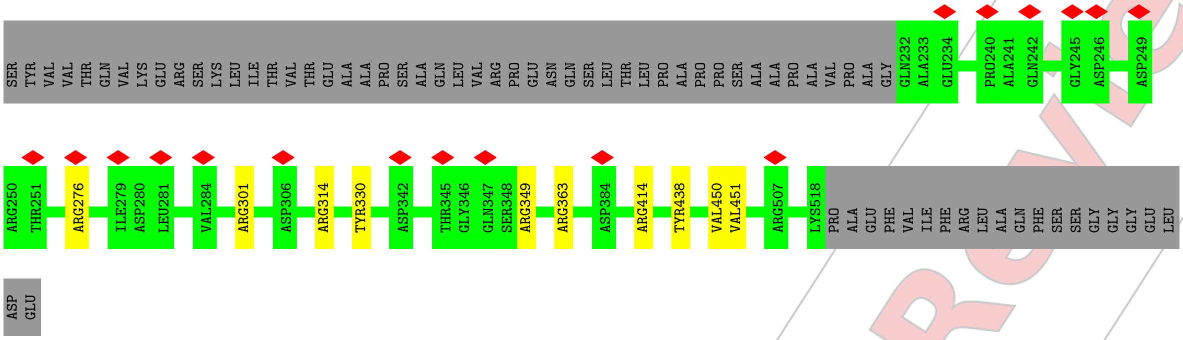

• Molecule 1: Putative phage tail sheath protein

Chain E:

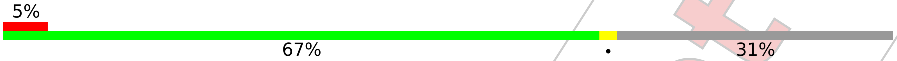

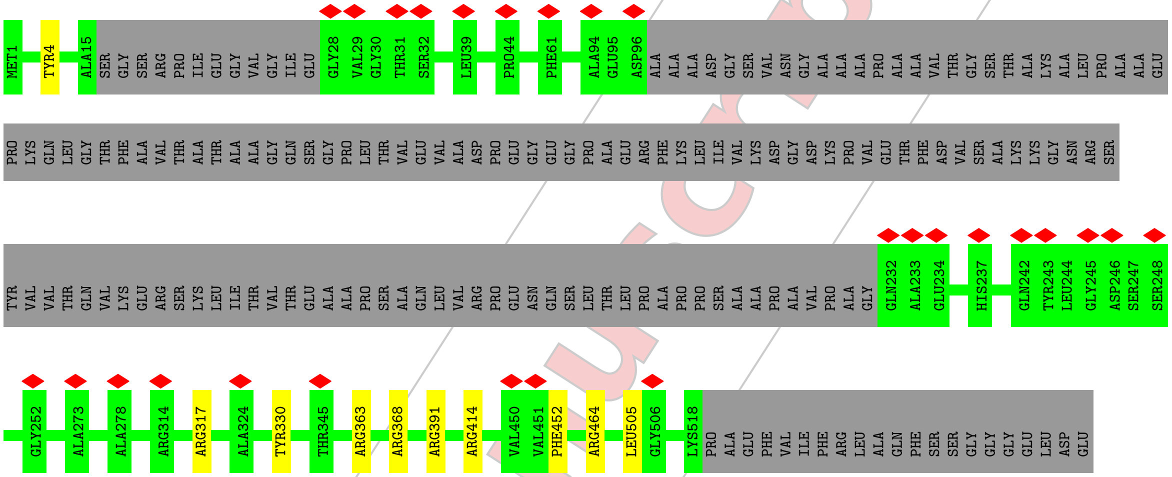

• Molecule 1: Putative phage tail sheath protein

Chain X:

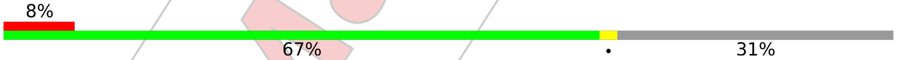

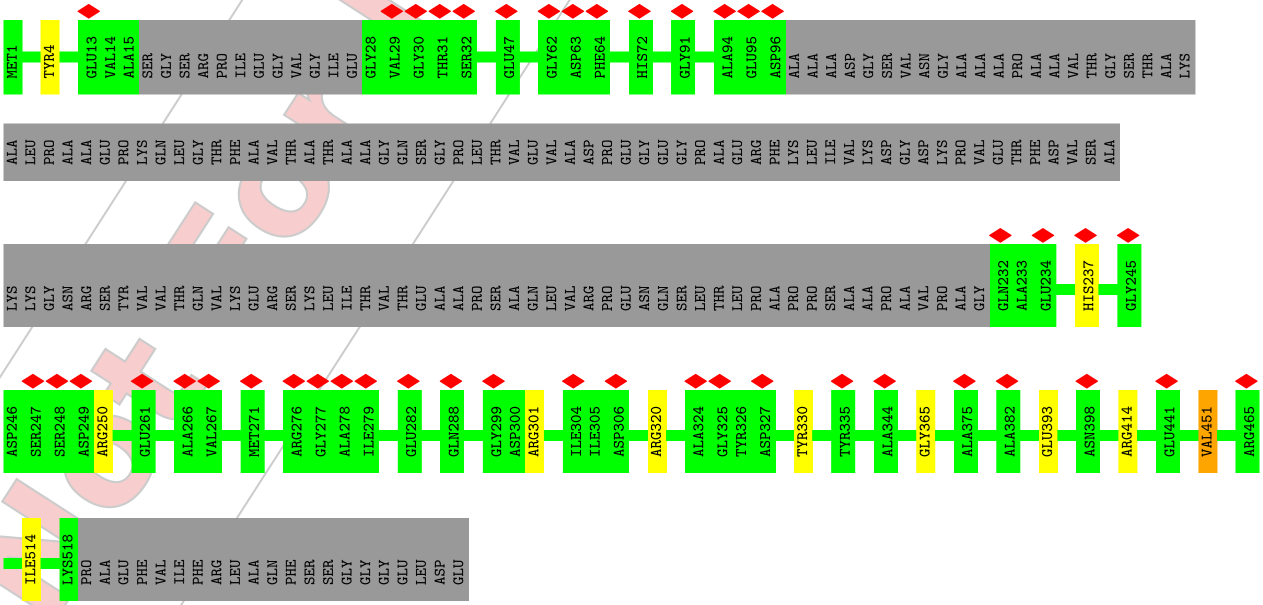

Not For Manuscript Review

• Molecule 1: Putative phage tail sheath protein

Chain R: 6% 66% • 31%

MET1 VAL9 GLU12 GLU13 VAL14 ALA15 SER GLY SER ARG PRO ILE GLU GLY VAL GLY ILE GLU GLY28 VAL29 GLY30 THR31 SER32 THR42 GLU47 PRO48 PHE61 GLY62 ASP63 ARG89 VAL90 GLU95 ASP96 ALA ALA ALA ASP GLY SER VAL ASN GLY ALA ALA ALA PRO ALA ALA VAL THR GLY SER THR

ALA LYS ALA LEU PRO ALA ALA GLU PRO LYS GLN LEU GLY THR PHE ALA VAL THR ALA THR ALA ALA GLY GLN SER GLY PRO LEU THR VAL GLU VAL ALA ASP PRO GLU GLY GLU GLY PRO ALA GLU ARG PHE LYS LEU ILE VAL LYS ASP GLY ASP LYS PRO VAL GLU THR PHE ASP VAL

SER ALA LYS LYS GLY ASN ARG SER TYR VAL VAL THR GLN VAL LYS GLU ARG SER LYS LEU ILE THR VAL THR GLU ALA ALA PRO SER ALA GLN LEU VAL ARG PRO GLU ASN GLN SER LEU THR LEU PRO ALA PRO PRO SER ALA ALA PRO ALA VAL PRO ALA GLY GLN232 ALA233 GLU234 SER235 ALA236

ASP246 SER247 SER248 ASP249 ARG250 THR251 GLY252 GLU261 ASP269 LEU270 TYR274 GLY277 ALA278 ASP306 TYR335 ASP342 GLU367 GLU377 ASP384 GLY410 ARG414 GLY417 GLU441 ASN453 PRO454 ASN455 ARG464 TYR490 ARG507 ILE514 ALA515 LYS518 PRO ALA GLU PHE VAL ILE PHE

ARG LEU ALA GLN PHE SER SER GLY GLY GLY GLU LEU ASP GLU

• Molecule 1: Putative phage tail sheath protein

Chain L: 5% 67% • 31%

MET1 VAL14 ALA15 SER GLY SER ARG PRO ILE GLU GLY VAL GLY ILE GLU GLY28 VAL29 SER32 GLU47 PHE61 TYR68 ARG89 VAL90 GLY91 GLU95 ASP96 ALA ALA ALA ASP GLY SER VAL ASN GLY ALA ALA ALA PRO ALA ALA VAL THR GLY SER THR ALA LYS ALA LEU PRO ALA ALA

GLU PRO LYS GLN LEU GLY THR PHE ALA VAL THR ALA THR ALA ALA GLY GLN SER GLY PRO LEU THR VAL GLU VAL ALA ASP PRO GLU GLY GLU GLY PRO ALA GLU ARG PHE LYS LEU ILE VAL LYS ASP GLY ASP LYS PRO VAL GLU THR PHE ASP VAL SER ALA LYS LYS GLY ASN ARG

SER TYR VAL VAL THR GLN VAL LYS GLU ARG SER LYS LEU ILE THR VAL THR GLU ALA ALA PRO SER ALA GLN LEU VAL ARG PRO GLU ASN GLN SER LEU THR LEU PRO ALA PRO PRO SER ALA ALA PRO ALA VAL PRO ALA GLY GLN232 ALA233 GLU234 PRO240 ALA241 GLN242 GLY245 ASP246 ASP249

ARG250 THR251 ARG276 ILE279 VAL284 ARG301 ASP306 ARG314 TYR330 ASP342 THR345 GLY346 GLN347 SER348 ARG349 ARG363 VAL370 ASP384 ARG414 TYR438 VAL450 VAL451 ARG507 LYS518 PRO ALA GLU PHE VAL ILE PHE ARG LEU ALA GLN PHE SER SER GLY GLY GLY GLU LEU

ASP GLU

• Molecule 1: Putative phage tail sheath protein

Chain F: • 67% • 31%

MET1 TYR4 ALA15 SER GLY SER ARG PRO ILE GLU GLY VAL GLY ILE GLU GLY28 VAL29 GLY30 THR31 LEU39 PRO44 PHE61 ALA94 GLU95 ASP96 ALA ALA ALA ASP GLY SER VAL ASN GLY ALA ALA ALA PRO ALA ALA VAL THR GLY SER THR ALA LYS ALA LEU PRO ALA ALA GLU PRO

Not For Manuscript Review

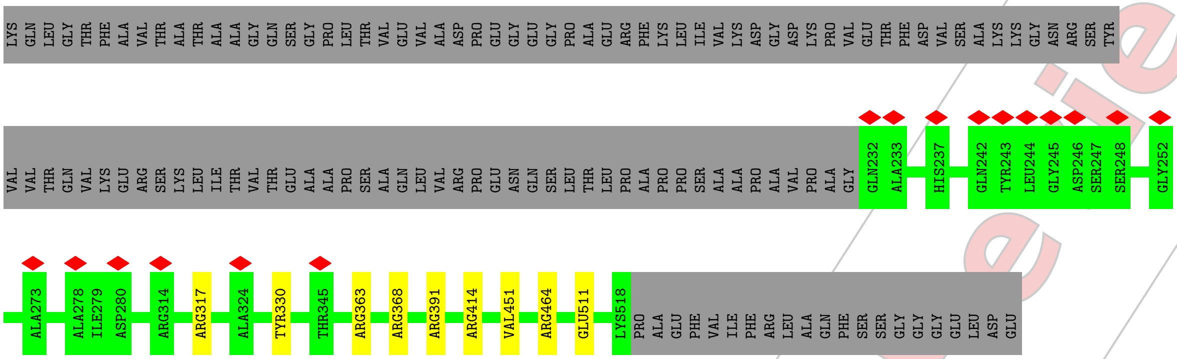

• Molecule 1: Putative phage tail sheath protein

Chain S:

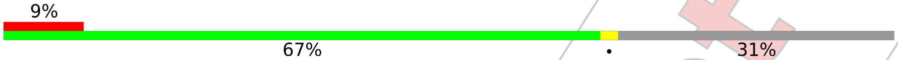

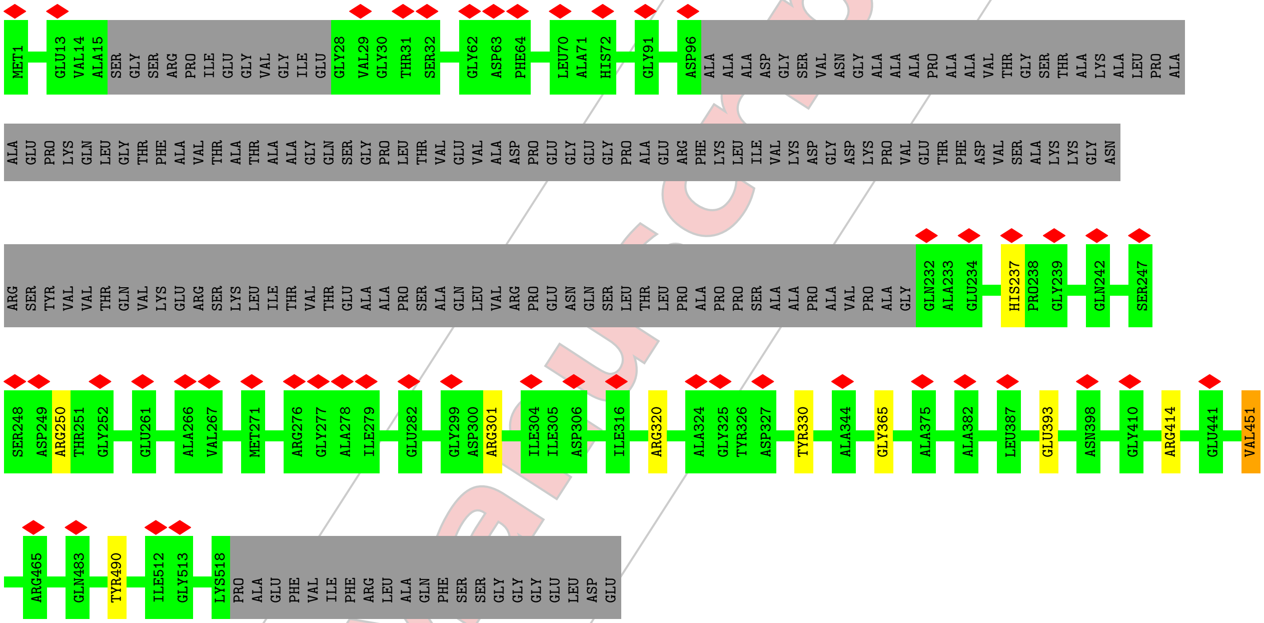

• Molecule 1: Putative phage tail sheath protein

Chain M:

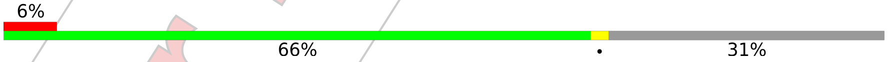

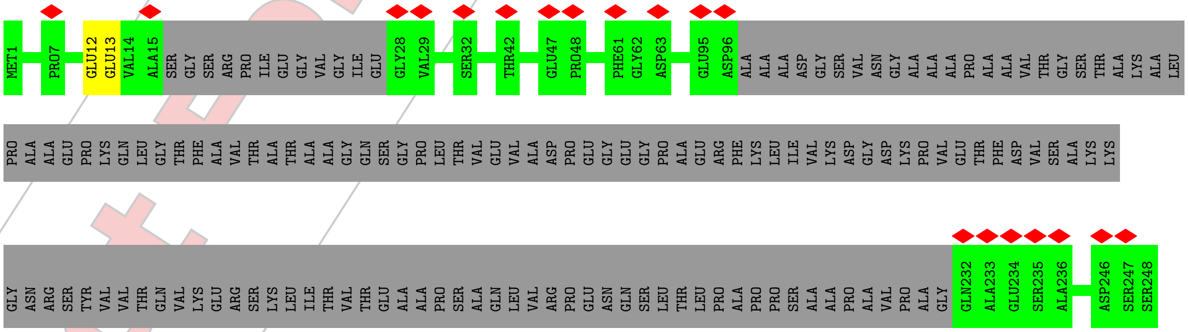

Not For Manuscript Review

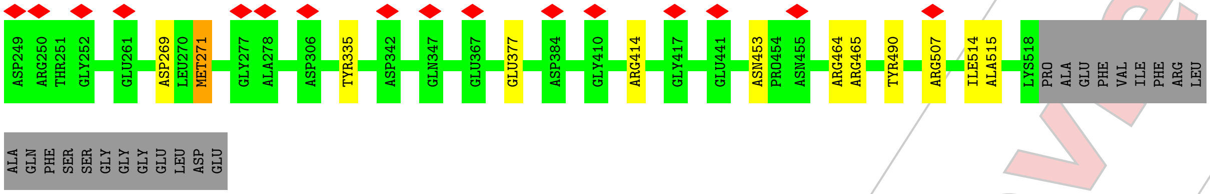

• Molecule 1: Putative phage tail sheath protein

Chain G:

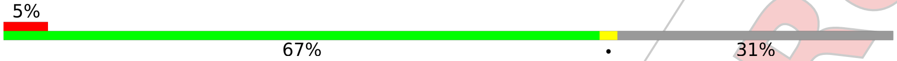

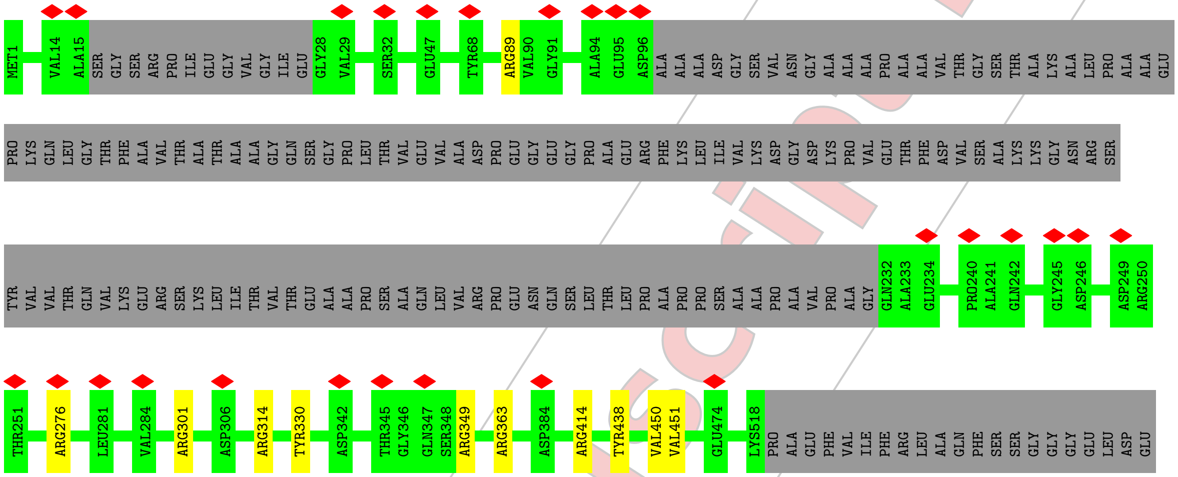

• Molecule 1: Putative phage tail sheath protein

Chain A:

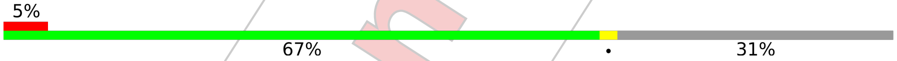

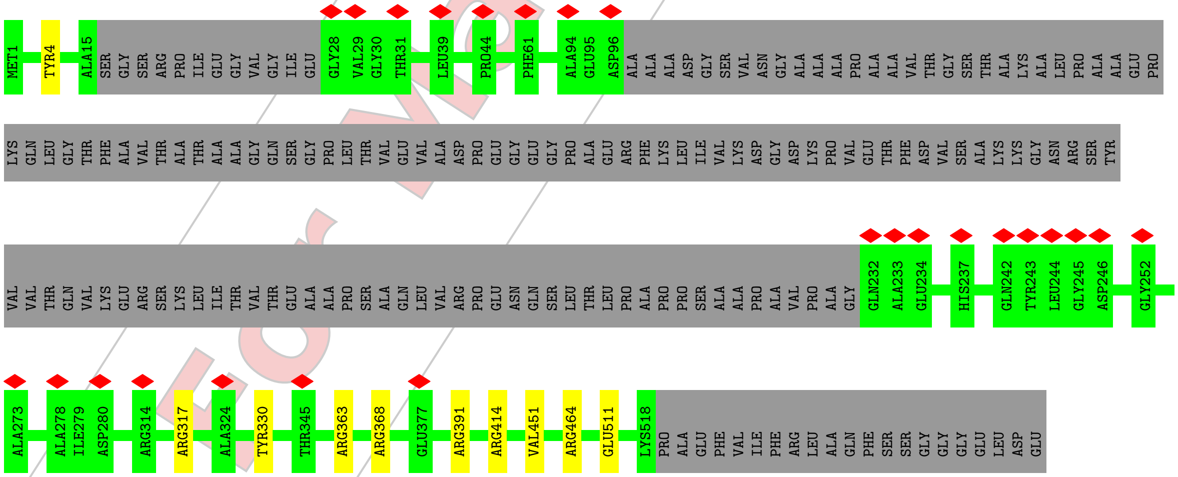

• Molecule 1: Putative phage tail sheath protein

Chain T:

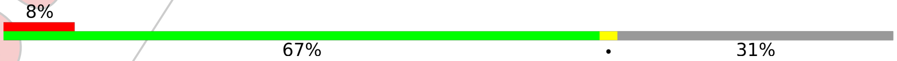

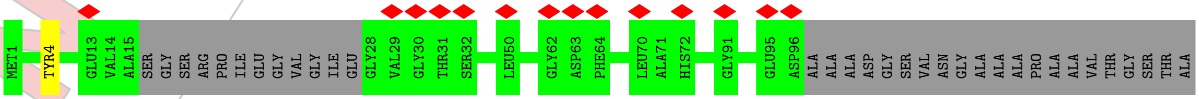

Not For Manuscript Review

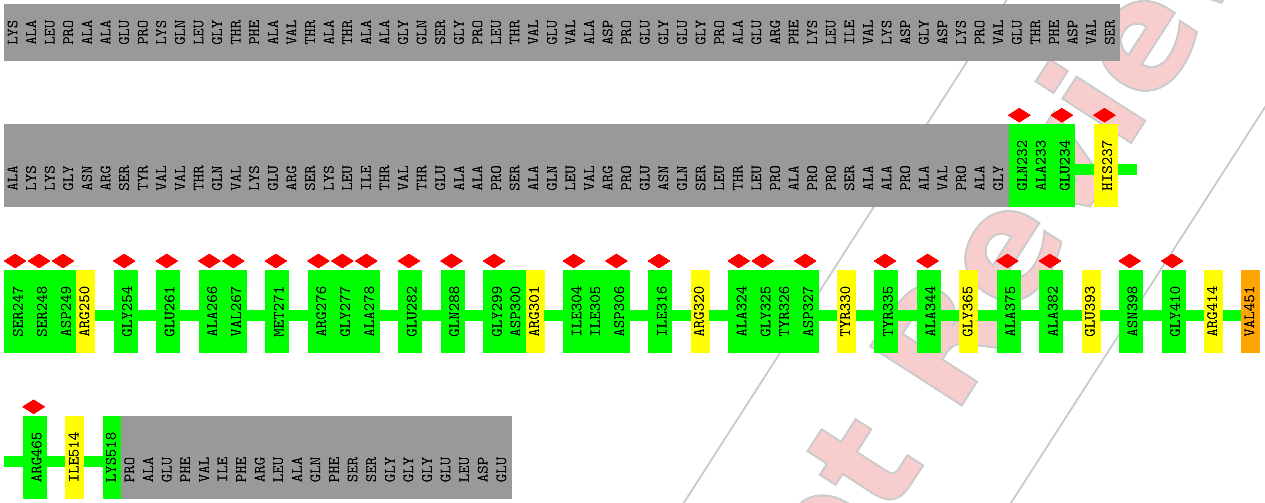

- Molecule 1: Putative phage tail sheath protein

Chain N:

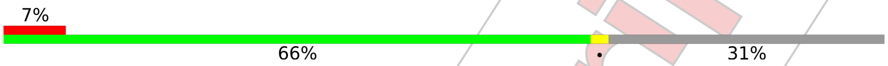

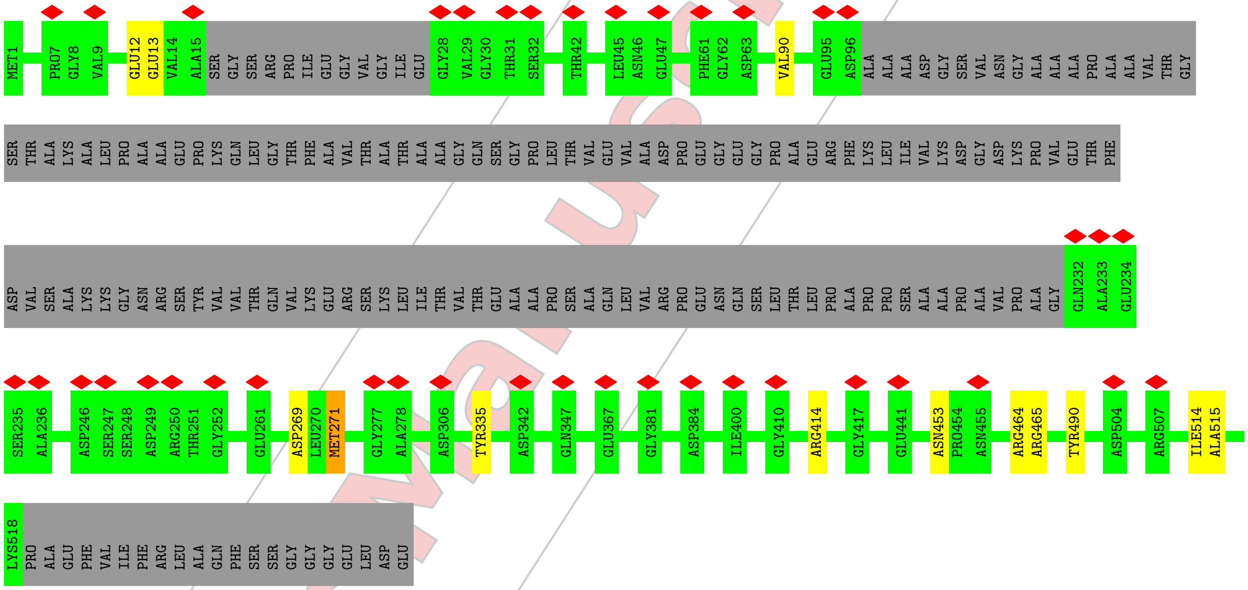

- Molecule 1: Putative phage tail sheath protein

Chain H:

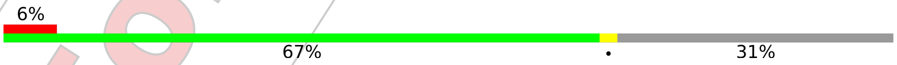

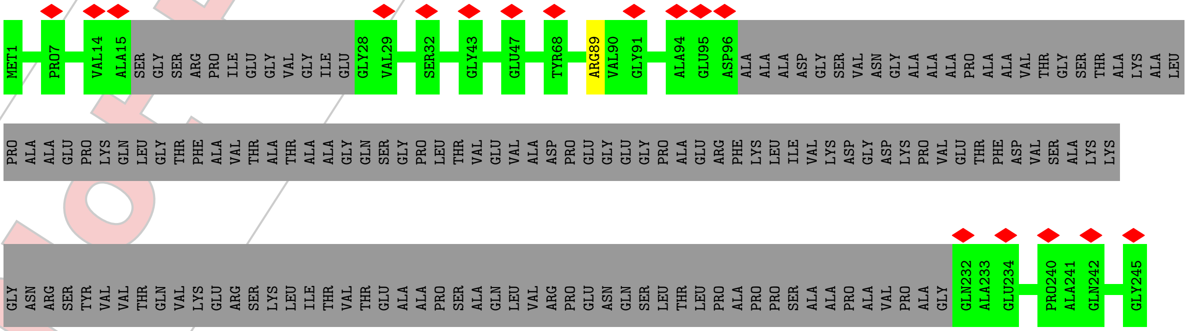

Not For Manuscript Review

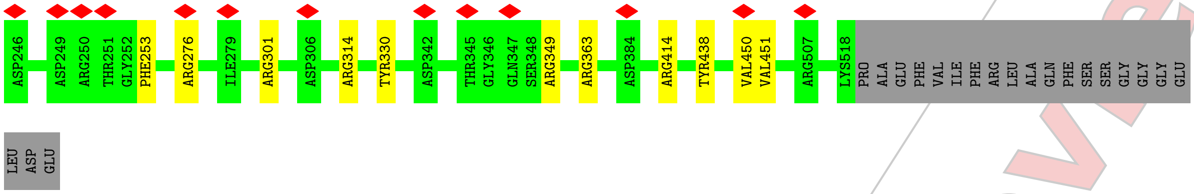

• Molecule 1: Putative phage tail sheath protein

Chain B:

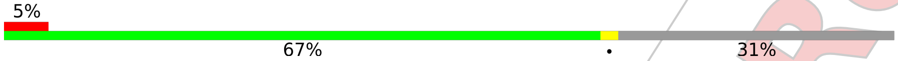

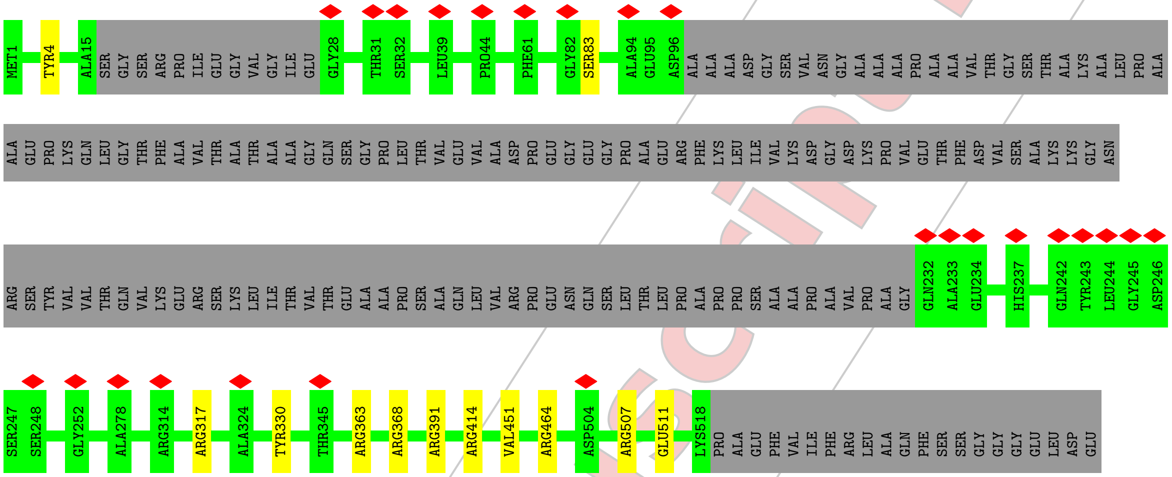

• Molecule 1: Putative phage tail sheath protein

Chain U:

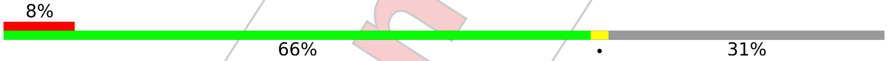

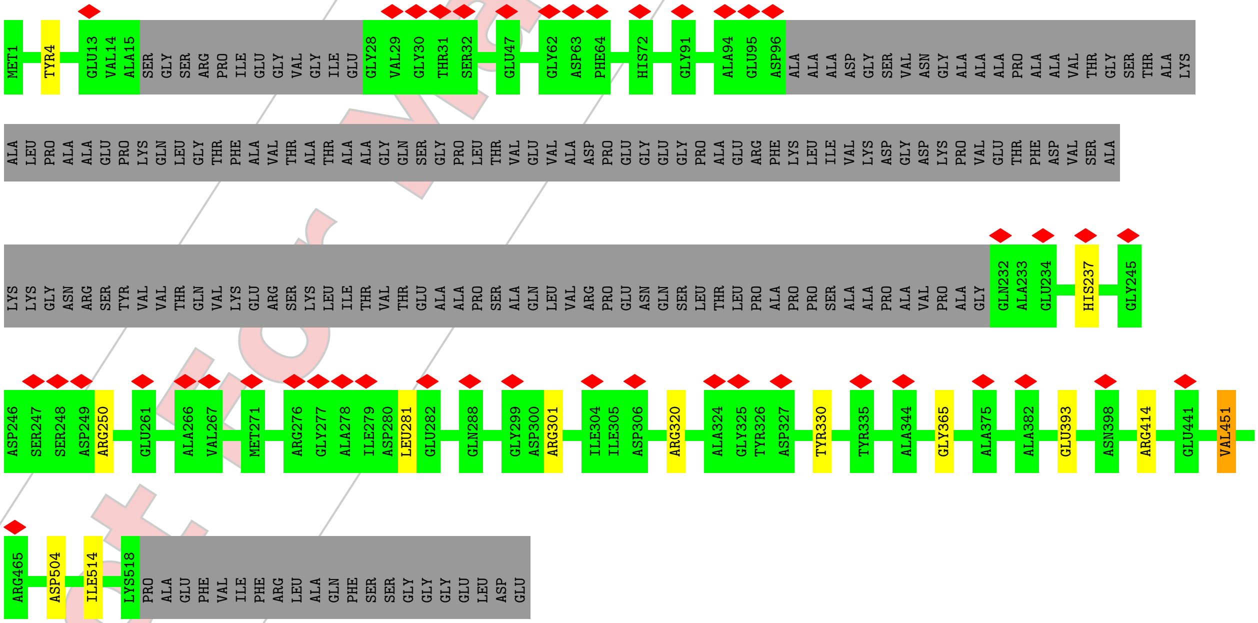

• Molecule 1: Putative phage tail sheath protein

Not For Manuscript Review

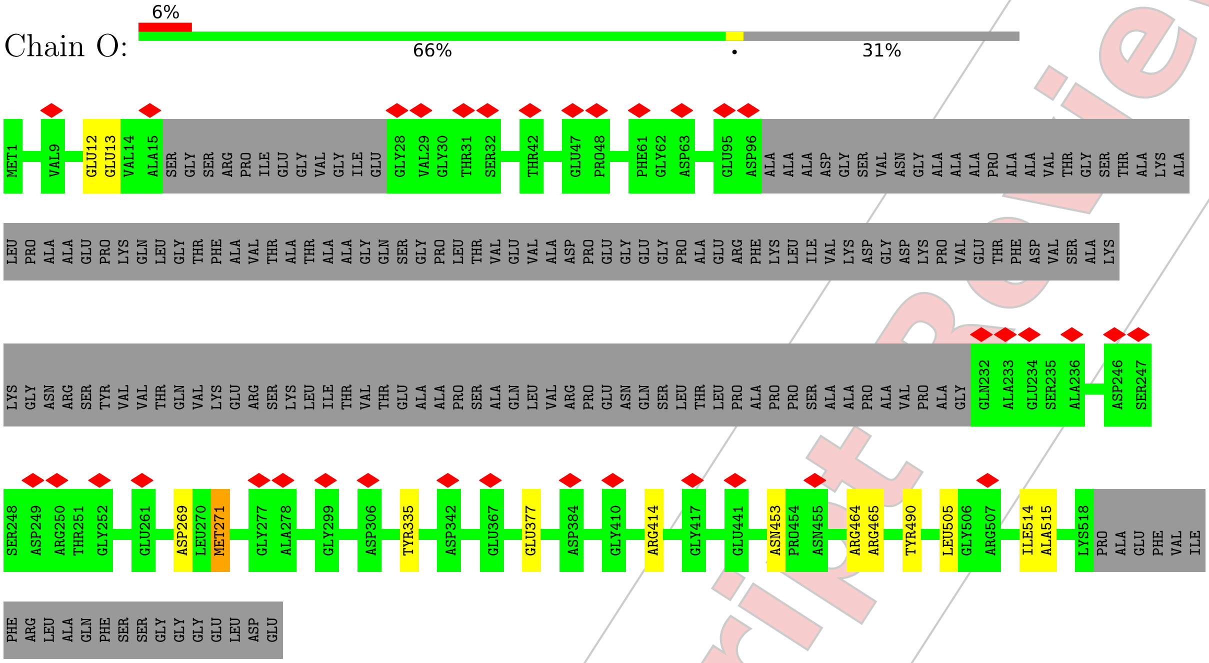

• Molecule 1: Putative phage tail sheath protein

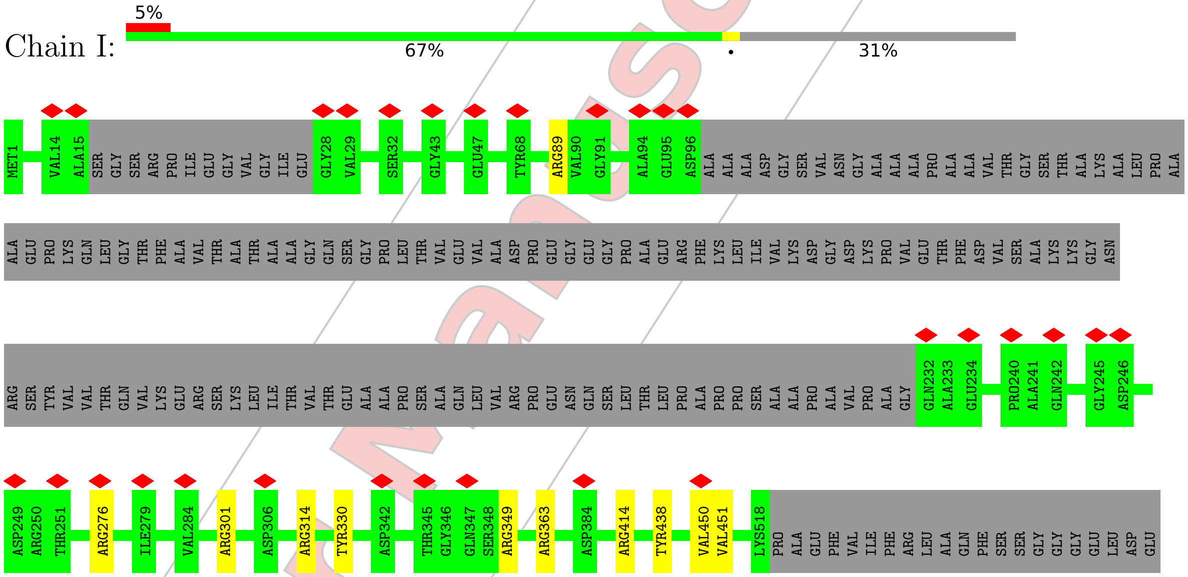

• Molecule 1: Putative phage tail sheath protein

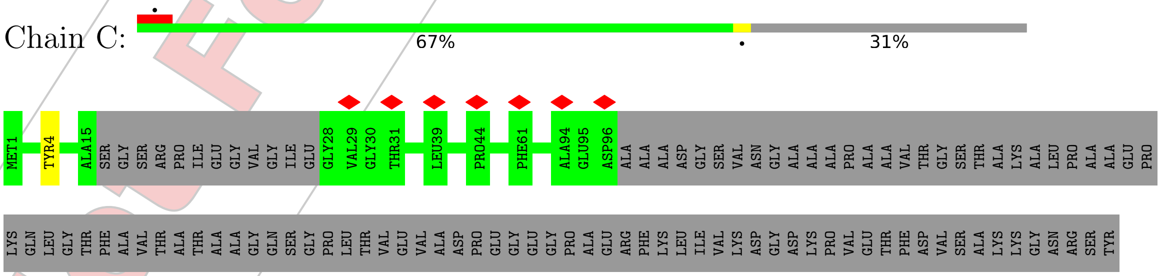

Not For Manuscript Review

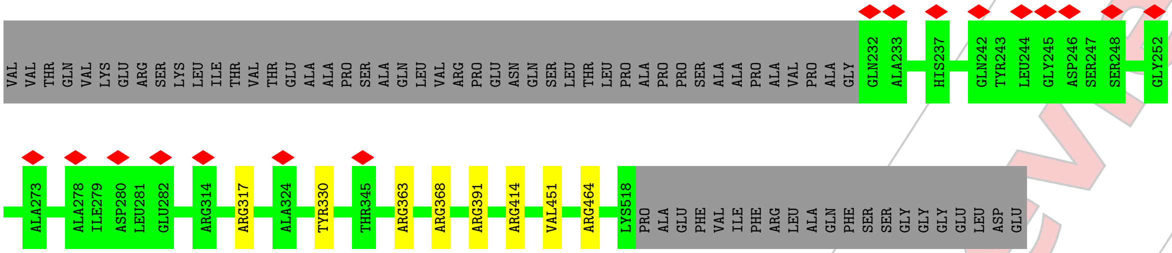

• Molecule 2: Phage tail protein

Chain v:

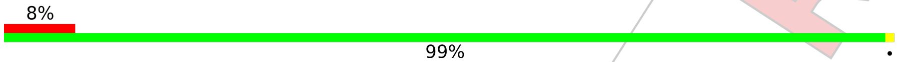

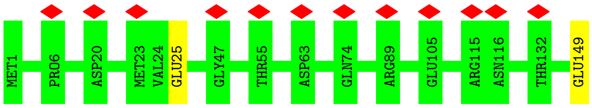

• Molecule 2: Phage tail protein

Chain p:

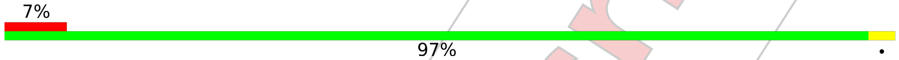

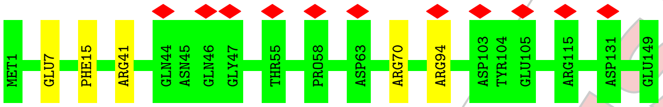

• Molecule 2: Phage tail protein

Chain j:

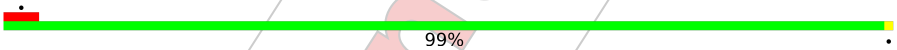

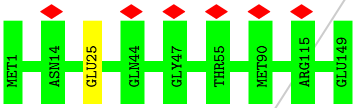

• Molecule 2: Phage tail protein

Chain d:

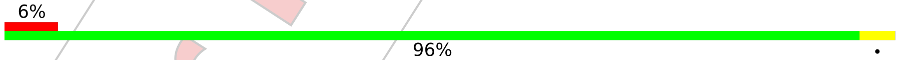

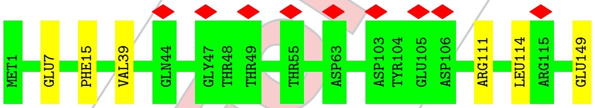

• Molecule 2: Phage tail protein

Chain w:

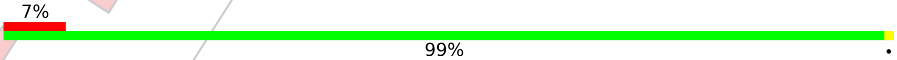

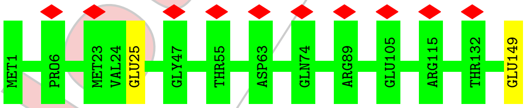

• Molecule 2: Phage tail protein

Not For Manuscript Review

Chain q:

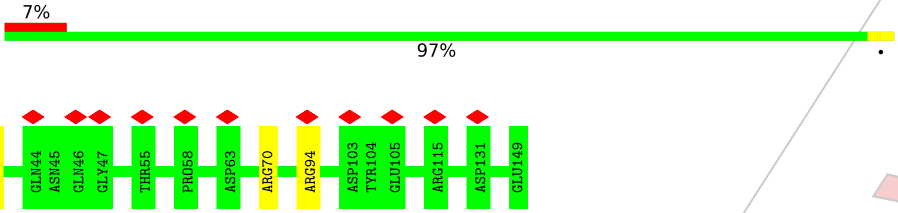

• Molecule 2: Phage tail protein

Chain k:

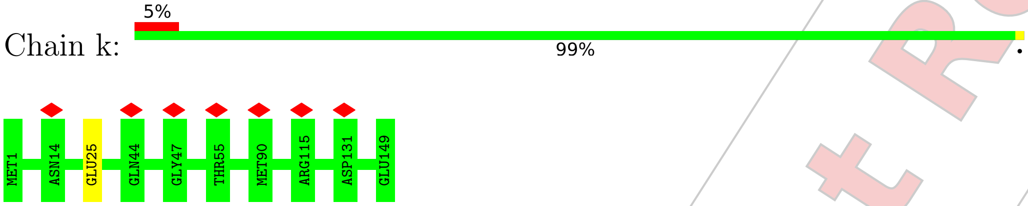

• Molecule 2: Phage tail protein

Chain e:

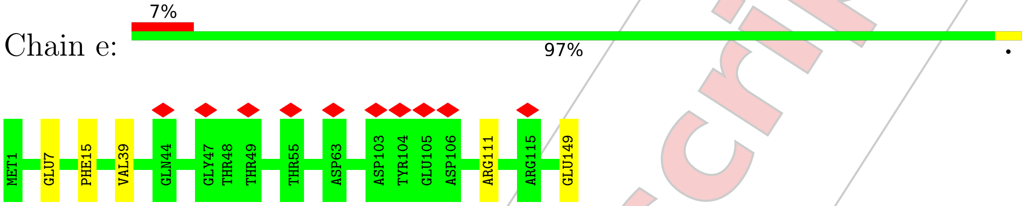

• Molecule 2: Phage tail protein

Chain x:

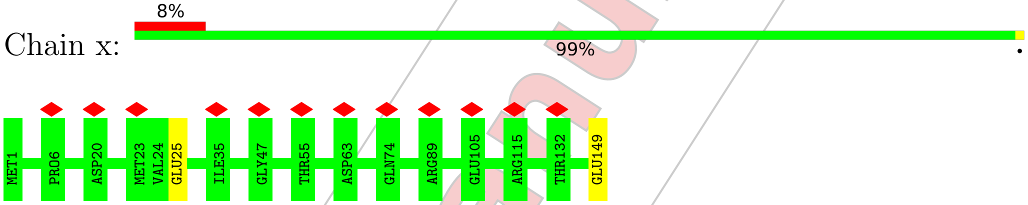

• Molecule 2: Phage tail protein

Chain r:

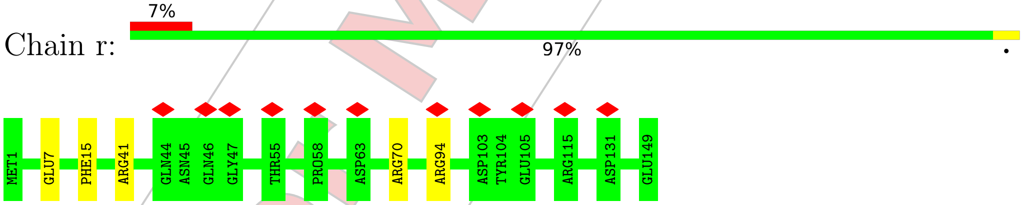

• Molecule 2: Phage tail protein

Chain l:

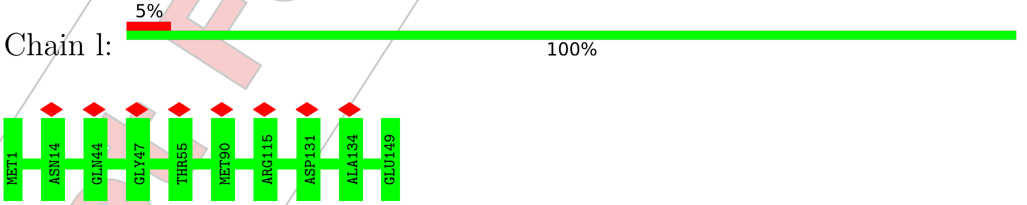

• Molecule 2: Phage tail protein

Chain f:

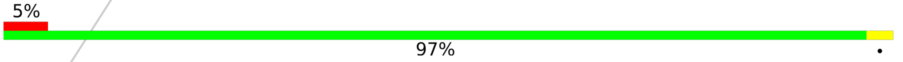

Not For Manuscript Review

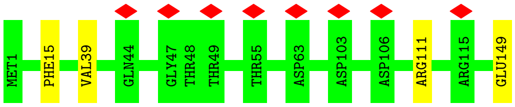

• Molecule 2: Phage tail protein

Chain s:
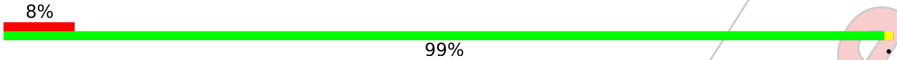

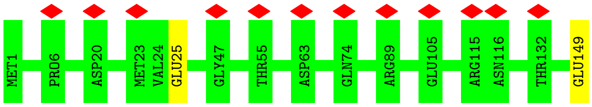

• Molecule 2: Phage tail protein

Chain m:
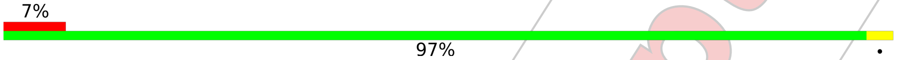

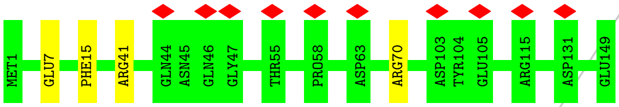

• Molecule 2: Phage tail protein

Chain g:
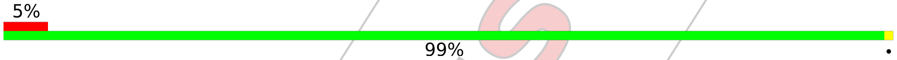

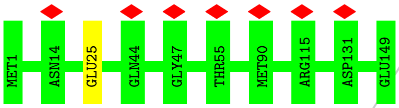

• Molecule 2: Phage tail protein

Chain a:
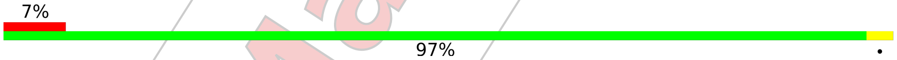

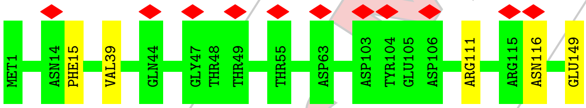

• Molecule 2: Phage tail protein

Chain t:
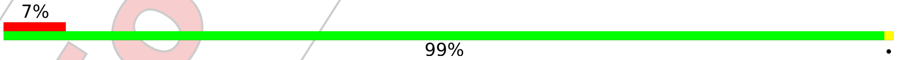

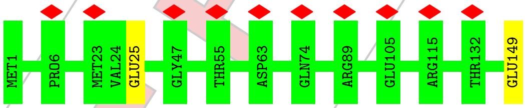

• Molecule 2: Phage tail protein

Chain n:
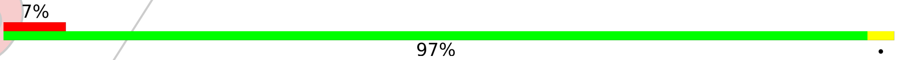

Not For Manuscript Review

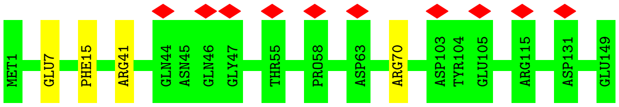

• Molecule 2: Phage tail protein

Chain h:

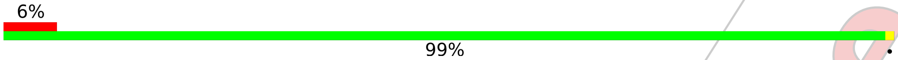

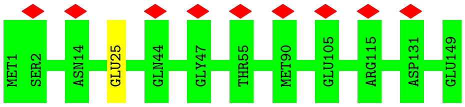

• Molecule 2: Phage tail protein

Chain b:

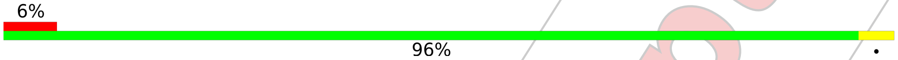

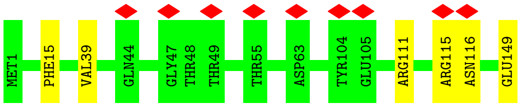

• Molecule 2: Phage tail protein

Chain u:

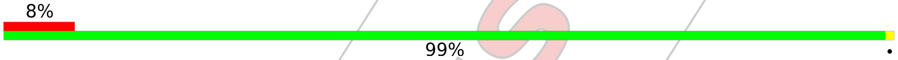

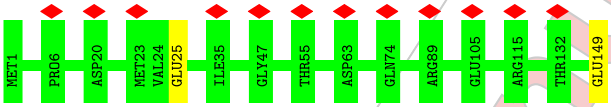

• Molecule 2: Phage tail protein

Chain o:

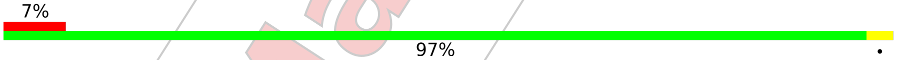

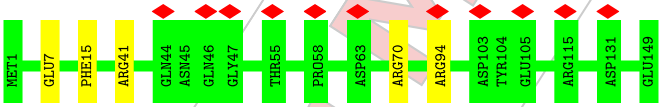

• Molecule 2: Phage tail protein

Chain i:

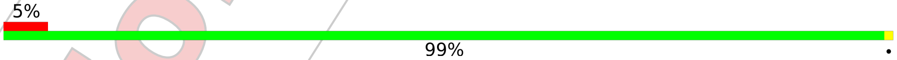

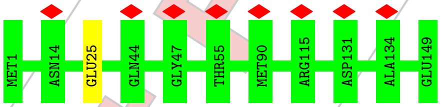

• Molecule 2: Phage tail protein

Chain c:

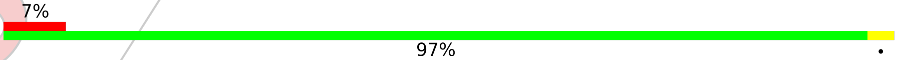

Not For Manuscript Review

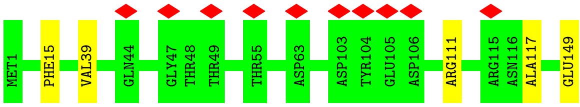

Not For Manuscript Review

4 Experimental information (i)

Property	Value	Source
EM reconstruction method	HELICAL	Depositor
Imposed symmetry	HELICAL, twist=23.10°, rise=38.50 Å, axial sym=C6	Depositor
Number of segments used	18822	Depositor
Resolution determination method	FSC 0.143 CUT-OFF	Depositor
CTF correction method	PHASE FLIPPING AND AMPLITUDE CORRECTION	Depositor
Microscope	FEI TITAN KRIOS	Depositor
Voltage (kV)	300	Depositor
Electron dose ($e^-/\text{Å}^2$)	60	Depositor
Minimum defocus (nm)	1500	Depositor
Maximum defocus (nm)	3500	Depositor
Magnification	Not provided	
Image detector	GATAN K2 SUMMIT (4k x 4k)	Depositor
Maximum map value	0.183	Depositor
Minimum map value	-0.093	Depositor
Average map value	0.001	Depositor
Map value standard deviation	0.010	Depositor
Recommended contour level	0.03	Depositor
Map size (Å)	448.0, 448.0, 448.0	wwPDB
Map dimensions	320, 320, 320	wwPDB
Map angles (°)	90.0, 90.0, 90.0	wwPDB
Pixel spacing (Å)	1.4, 1.4, 1.4	Depositor

Not For Manuscript Review

5 Model quality (i)

5.1 Standard geometry (i)

The Z score for a bond length (or angle) is the number of standard deviations the observed value is removed from the expected value. A bond length (or angle) with $|Z| > 5$ is considered an outlier worth inspection. RMSZ is the root-mean-square of all Z scores of the bond lengths (or angles).

Mol	Chain	Bond lengths		Bond angles	
		RMSZ	#|Z| >5	RMSZ	#|Z| >5
1	A	1.01	1/2941 (0.0%)	0.95	11/4013 (0.3%)
1	B	1.01	1/2941 (0.0%)	0.95	11/4013 (0.3%)
1	C	1.01	0/2941	0.95	11/4013 (0.3%)
1	D	1.01	1/2941 (0.0%)	0.95	11/4013 (0.3%)
1	E	1.01	0/2941	0.94	11/4013 (0.3%)
1	F	1.01	1/2941 (0.0%)	0.95	11/4013 (0.3%)
1	G	0.96	1/2941 (0.0%)	0.96	9/4013 (0.2%)
1	H	0.96	1/2941 (0.0%)	0.96	9/4013 (0.2%)
1	I	0.96	1/2941 (0.0%)	0.96	9/4013 (0.2%)
1	J	0.96	1/2941 (0.0%)	0.96	9/4013 (0.2%)
1	K	0.96	1/2941 (0.0%)	0.96	9/4013 (0.2%)
1	L	0.96	1/2941 (0.0%)	0.96	9/4013 (0.2%)
1	M	1.02	4/2941 (0.1%)	0.88	5/4013 (0.1%)
1	N	1.02	4/2941 (0.1%)	0.89	5/4013 (0.1%)
1	O	1.02	5/2941 (0.2%)	0.89	5/4013 (0.1%)
1	P	1.01	5/2941 (0.2%)	0.89	5/4013 (0.1%)
1	Q	1.02	4/2941 (0.1%)	0.89	4/4013 (0.1%)
1	R	1.00	5/2941 (0.2%)	0.88	4/4013 (0.1%)
1	S	0.97	4/2941 (0.1%)	0.94	5/4013 (0.1%)
1	T	0.98	3/2941 (0.1%)	0.96	5/4013 (0.1%)
1	U	0.98	3/2941 (0.1%)	0.96	5/4013 (0.1%)
1	V	0.98	3/2941 (0.1%)	0.96	5/4013 (0.1%)
1	W	0.98	3/2941 (0.1%)	0.96	5/4013 (0.1%)
1	X	0.98	3/2941 (0.1%)	0.96	5/4013 (0.1%)
2	a	0.99	3/1166 (0.3%)	0.97	1/1585 (0.1%)
2	b	0.99	3/1166 (0.3%)	0.97	1/1585 (0.1%)
2	c	0.99	3/1166 (0.3%)	0.96	1/1585 (0.1%)
2	d	1.03	4/1166 (0.3%)	0.97	1/1585 (0.1%)
2	e	0.99	4/1166 (0.3%)	0.97	1/1585 (0.1%)
2	f	1.00	3/1166 (0.3%)	0.96	1/1585 (0.1%)
2	g	1.02	1/1166 (0.1%)	0.97	0/1585
2	h	1.02	1/1166 (0.1%)	0.97	0/1585
2	i	1.02	1/1166 (0.1%)	0.97	0/1585
2	j	1.02	1/1166 (0.1%)	0.97	0/1585

Not For Manuscript Review

Mol	Chain	Bond lengths		Bond angles	
		RMSZ	#|Z| >5	RMSZ	#|Z| >5
2	k	1.02	1/1166 (0.1%)	0.97	0/1585
2	l	1.02	0/1166	0.97	0/1585
2	m	0.97	2/1166 (0.2%)	0.97	2/1585 (0.1%)
2	n	0.97	2/1166 (0.2%)	0.96	2/1585 (0.1%)
2	o	0.97	2/1166 (0.2%)	0.97	2/1585 (0.1%)
2	p	0.97	2/1166 (0.2%)	0.97	2/1585 (0.1%)
2	q	0.97	2/1166 (0.2%)	0.97	2/1585 (0.1%)
2	r	0.97	2/1166 (0.2%)	0.96	2/1585 (0.1%)
2	s	0.96	3/1166 (0.3%)	0.98	0/1585
2	t	0.96	3/1166 (0.3%)	0.98	0/1585
2	u	0.96	3/1166 (0.3%)	0.97	0/1585
2	v	0.96	3/1166 (0.3%)	0.97	0/1585
2	w	0.96	3/1166 (0.3%)	0.97	0/1585
2	x	0.96	3/1166 (0.3%)	0.97	0/1585
All	All	0.99	111/98568 (0.1%)	0.95	196/134352 (0.1%)

All (111) bond length outliers are listed below:

Mol	Chain	Res	Type	Atoms	Z	Observed(Å)	Ideal(Å)
2	u	25	GLU	CD-OE1	-6.41	1.18	1.25
2	v	25	GLU	CD-OE1	-6.38	1.18	1.25
2	x	25	GLU	CD-OE1	-6.36	1.18	1.25
2	t	25	GLU	CD-OE1	-6.36	1.18	1.25
2	s	25	GLU	CD-OE1	-6.33	1.18	1.25
2	f	39	VAL	CB-CG1	-6.30	1.39	1.52
2	b	39	VAL	CB-CG1	-6.30	1.39	1.52
2	w	25	GLU	CD-OE1	-6.29	1.18	1.25
2	e	39	VAL	CB-CG1	-6.29	1.39	1.52
2	m	7	GLU	CD-OE1	-6.28	1.18	1.25
2	a	39	VAL	CB-CG1	-6.28	1.39	1.52
2	c	39	VAL	CB-CG1	-6.27	1.39	1.52
2	d	39	VAL	CB-CG1	-6.27	1.39	1.52
2	o	7	GLU	CD-OE1	-6.26	1.18	1.25
2	p	7	GLU	CD-OE1	-6.24	1.18	1.25
2	q	7	GLU	CD-OE1	-6.23	1.18	1.25
2	n	7	GLU	CD-OE1	-6.22	1.18	1.25
2	r	7	GLU	CD-OE1	-6.19	1.18	1.25
1	M	12	GLU	CD-OE2	-6.12	1.19	1.25
1	O	12	GLU	CD-OE2	-6.08	1.19	1.25
1	N	12	GLU	CD-OE2	-6.06	1.19	1.25
1	P	12	GLU	CD-OE2	-6.01	1.19	1.25
1	Q	12	GLU	CD-OE2	-6.00	1.19	1.25

Continued on next page...

Not For Manuscript Review

Continued from previous page...

Mol	Chain	Res	Type	Atoms	Z	Observed(Å)	Ideal(Å)
1	R	12	GLU	CD-OE2	-5.97	1.19	1.25
1	S	330	TYR	CB-CG	-5.86	1.42	1.51
2	e	7	GLU	CD-OE1	-5.85	1.19	1.25
1	V	330	TYR	CB-CG	-5.84	1.42	1.51
1	T	330	TYR	CB-CG	-5.83	1.43	1.51
1	X	330	TYR	CB-CG	-5.83	1.43	1.51
1	W	330	TYR	CB-CG	-5.82	1.43	1.51
1	U	330	TYR	CB-CG	-5.81	1.43	1.51
2	d	7	GLU	CD-OE1	-5.76	1.19	1.25
1	X	393	GLU	CD-OE1	-5.68	1.19	1.25
1	T	393	GLU	CD-OE1	-5.65	1.19	1.25
1	V	393	GLU	CD-OE1	-5.64	1.19	1.25
1	S	393	GLU	CD-OE1	-5.64	1.19	1.25
1	U	393	GLU	CD-OE1	-5.63	1.19	1.25
2	s	25	GLU	CG-CD	-5.61	1.43	1.51
1	Q	490	TYR	CB-CG	-5.59	1.43	1.51
1	R	490	TYR	CB-CG	-5.59	1.43	1.51
1	W	393	GLU	CD-OE1	-5.58	1.19	1.25
1	M	490	TYR	CB-CG	-5.57	1.43	1.51
2	t	25	GLU	CG-CD	-5.57	1.43	1.51
2	u	25	GLU	CG-CD	-5.57	1.43	1.51
2	w	25	GLU	CG-CD	-5.57	1.43	1.51
2	x	25	GLU	CG-CD	-5.57	1.43	1.51
1	N	490	TYR	CB-CG	-5.56	1.43	1.51
2	v	25	GLU	CG-CD	-5.56	1.43	1.51
1	P	490	TYR	CB-CG	-5.54	1.43	1.51
1	O	490	TYR	CB-CG	-5.53	1.43	1.51
1	J	450	VAL	CB-CG1	-5.41	1.41	1.52
1	K	450	VAL	CB-CG1	-5.37	1.41	1.52
1	H	450	VAL	CB-CG1	-5.37	1.41	1.52
1	I	450	VAL	CB-CG1	-5.37	1.41	1.52
1	L	450	VAL	CB-CG1	-5.36	1.41	1.52
1	G	450	VAL	CB-CG1	-5.36	1.41	1.52
2	f	149	GLU	CB-CG	5.35	1.62	1.52
2	b	149	GLU	CB-CG	5.35	1.62	1.52
2	c	149	GLU	CB-CG	5.34	1.62	1.52
2	a	149	GLU	CB-CG	5.33	1.62	1.52
2	n	15	PHE	CB-CG	-5.32	1.42	1.51
2	d	149	GLU	CB-CG	5.31	1.62	1.52
2	e	149	GLU	CB-CG	5.30	1.62	1.52
2	m	15	PHE	CB-CG	-5.28	1.42	1.51
2	q	15	PHE	CB-CG	-5.27	1.42	1.51

Continued on next page...

Not For Manuscript Review

Continued from previous page...

Mol	Chain	Res	Type	Atoms	Z	Observed(Å)	Ideal(Å)
2	r	15	PHE	CB-CG	-5.26	1.42	1.51
2	o	15	PHE	CB-CG	-5.26	1.42	1.51
2	b	15	PHE	CB-CG	-5.25	1.42	1.51
2	d	15	PHE	CB-CG	-5.24	1.42	1.51
2	a	15	PHE	CB-CG	-5.24	1.42	1.51
2	p	15	PHE	CB-CG	-5.22	1.42	1.51
2	c	15	PHE	CB-CG	-5.22	1.42	1.51
2	f	15	PHE	CB-CG	-5.21	1.42	1.51
2	v	149	GLU	CB-CG	5.20	1.62	1.52
2	e	15	PHE	CB-CG	-5.19	1.42	1.51
2	w	149	GLU	CB-CG	5.19	1.62	1.52
2	t	149	GLU	CB-CG	5.17	1.61	1.52
2	x	149	GLU	CB-CG	5.16	1.61	1.52
2	u	149	GLU	CB-CG	5.16	1.61	1.52
1	P	13	GLU	CD-OE2	-5.16	1.20	1.25
1	M	13	GLU	CD-OE2	-5.16	1.20	1.25
2	s	149	GLU	CB-CG	5.15	1.61	1.52
1	Q	13	GLU	CD-OE2	-5.15	1.20	1.25
1	O	13	GLU	CD-OE2	-5.13	1.20	1.25
1	U	393	GLU	CG-CD	-5.09	1.44	1.51
2	g	25	GLU	CD-OE1	-5.09	1.20	1.25
1	V	393	GLU	CG-CD	-5.09	1.44	1.51
1	R	13	GLU	CD-OE2	-5.09	1.20	1.25
1	W	393	GLU	CG-CD	-5.08	1.44	1.51
2	h	25	GLU	CD-OE1	-5.08	1.20	1.25
1	B	511	GLU	CD-OE2	-5.07	1.20	1.25
2	j	25	GLU	CD-OE1	-5.07	1.20	1.25
2	i	25	GLU	CD-OE1	-5.06	1.20	1.25
1	T	393	GLU	CG-CD	-5.06	1.44	1.51
1	O	13	GLU	CD-OE1	-5.05	1.20	1.25
1	Q	377	GLU	CD-OE2	-5.05	1.20	1.25
2	k	25	GLU	CD-OE1	-5.05	1.20	1.25
1	D	511	GLU	CD-OE2	-5.04	1.20	1.25
1	R	13	GLU	CD-OE1	-5.04	1.20	1.25
1	X	393	GLU	CG-CD	-5.04	1.44	1.51
1	S	490	TYR	CB-CG	-5.04	1.44	1.51
1	P	13	GLU	CD-OE1	-5.03	1.20	1.25
1	S	393	GLU	CG-CD	-5.03	1.44	1.51
1	N	13	GLU	CD-OE2	-5.03	1.20	1.25
1	M	377	GLU	CD-OE2	-5.02	1.20	1.25
1	F	511	GLU	CD-OE2	-5.02	1.20	1.25
1	N	13	GLU	CD-OE1	-5.01	1.20	1.25

Continued on next page...

Not For Manuscript Review

Continued from previous page...

Mol	Chain	Res	Type	Atoms	Z	Observed(Å)	Ideal(Å)
1	A	511	GLU	CD-OE2	-5.01	1.20	1.25
1	P	377	GLU	CD-OE2	-5.00	1.20	1.25
1	R	377	GLU	CD-OE2	-5.00	1.20	1.25
1	O	377	GLU	CD-OE2	-5.00	1.20	1.25

All (196) bond angle outliers are listed below:

Mol	Chain	Res	Type	Atoms	Z	Observed(^{o})	Ideal(^{o})
1	X	301	ARG	NE-CZ-NH1	10.12	125.36	120.30
1	V	301	ARG	NE-CZ-NH1	10.12	125.36	120.30
1	W	301	ARG	NE-CZ-NH1	10.05	125.33	120.30
1	S	301	ARG	NE-CZ-NH1	10.01	125.31	120.30
1	U	301	ARG	NE-CZ-NH1	9.97	125.28	120.30
1	T	301	ARG	NE-CZ-NH1	9.95	125.27	120.30
1	G	314	ARG	NE-CZ-NH2	-9.34	115.63	120.30
1	H	314	ARG	NE-CZ-NH2	-9.34	115.63	120.30
1	K	314	ARG	NE-CZ-NH2	-9.31	115.64	120.30
1	J	314	ARG	NE-CZ-NH2	-9.29	115.66	120.30
1	L	314	ARG	NE-CZ-NH2	-9.29	115.66	120.30
2	b	111	ARG	NE-CZ-NH2	-9.27	115.66	120.30
1	I	314	ARG	NE-CZ-NH2	-9.27	115.67	120.30
2	d	111	ARG	NE-CZ-NH2	-9.24	115.68	120.30
2	c	111	ARG	NE-CZ-NH2	-9.23	115.69	120.30
2	e	111	ARG	NE-CZ-NH2	-9.22	115.69	120.30
2	f	111	ARG	NE-CZ-NH2	-9.21	115.69	120.30
2	a	111	ARG	NE-CZ-NH2	-9.21	115.70	120.30
1	P	414	ARG	NE-CZ-NH2	-9.19	115.70	120.30
1	O	414	ARG	NE-CZ-NH2	-9.15	115.72	120.30
1	N	414	ARG	NE-CZ-NH2	-9.14	115.73	120.30
1	R	414	ARG	NE-CZ-NH2	-9.12	115.74	120.30
1	M	414	ARG	NE-CZ-NH2	-9.06	115.77	120.30
1	Q	414	ARG	NE-CZ-NH2	-9.04	115.78	120.30
1	B	414	ARG	NE-CZ-NH2	-8.96	115.82	120.30
1	D	414	ARG	NE-CZ-NH2	-8.90	115.85	120.30
1	K	414	ARG	NE-CZ-NH2	-8.88	115.86	120.30
1	F	414	ARG	NE-CZ-NH2	-8.85	115.88	120.30
1	J	414	ARG	NE-CZ-NH2	-8.83	115.89	120.30
1	E	414	ARG	NE-CZ-NH2	-8.82	115.89	120.30
1	C	414	ARG	NE-CZ-NH2	-8.82	115.89	120.30
1	A	414	ARG	NE-CZ-NH2	-8.81	115.90	120.30
1	G	414	ARG	NE-CZ-NH2	-8.79	115.90	120.30
1	I	414	ARG	NE-CZ-NH2	-8.79	115.90	120.30

Continued on next page...

Not For Manuscript Review

Continued from previous page...

Mol	Chain	Res	Type	Atoms	Z	Observed(^{o})	Ideal(^{o})
1	H	414	ARG	NE-CZ-NH2	-8.76	115.92	120.30
1	L	414	ARG	NE-CZ-NH2	-8.72	115.94	120.30
1	F	464	ARG	NE-CZ-NH1	7.97	124.29	120.30
1	E	464	ARG	NE-CZ-NH1	7.97	124.28	120.30
1	C	464	ARG	NE-CZ-NH1	7.97	124.28	120.30
1	X	414	ARG	NE-CZ-NH2	-7.92	116.34	120.30
1	T	414	ARG	NE-CZ-NH2	-7.90	116.35	120.30
1	A	464	ARG	NE-CZ-NH1	7.89	124.24	120.30
1	D	464	ARG	NE-CZ-NH1	7.88	124.24	120.30
1	U	414	ARG	NE-CZ-NH2	-7.88	116.36	120.30
1	V	414	ARG	NE-CZ-NH2	-7.87	116.37	120.30
1	B	464	ARG	NE-CZ-NH1	7.86	124.23	120.30
1	S	414	ARG	NE-CZ-NH2	-7.84	116.38	120.30
1	W	414	ARG	NE-CZ-NH2	-7.79	116.40	120.30
1	E	363	ARG	NE-CZ-NH2	-7.56	116.52	120.30
1	F	363	ARG	NE-CZ-NH2	-7.50	116.55	120.30
1	B	363	ARG	NE-CZ-NH2	-7.50	116.55	120.30
1	A	363	ARG	NE-CZ-NH2	-7.49	116.56	120.30
1	C	363	ARG	NE-CZ-NH2	-7.44	116.58	120.30
1	D	363	ARG	NE-CZ-NH2	-7.41	116.60	120.30
1	D	317	ARG	NE-CZ-NH1	7.18	123.89	120.30
1	C	317	ARG	NE-CZ-NH1	7.17	123.89	120.30
1	A	317	ARG	NE-CZ-NH1	7.17	123.89	120.30
1	B	317	ARG	NE-CZ-NH1	7.17	123.88	120.30
1	E	317	ARG	NE-CZ-NH1	7.14	123.87	120.30
1	B	414	ARG	NE-CZ-NH1	7.14	123.87	120.30
1	F	414	ARG	NE-CZ-NH1	7.12	123.86	120.30
1	C	414	ARG	NE-CZ-NH1	7.12	123.86	120.30
1	D	414	ARG	NE-CZ-NH1	7.11	123.86	120.30
1	F	317	ARG	NE-CZ-NH1	7.10	123.85	120.30
1	E	414	ARG	NE-CZ-NH1	7.08	123.84	120.30
1	A	414	ARG	NE-CZ-NH1	7.07	123.83	120.30
1	P	414	ARG	NE-CZ-NH1	6.90	123.75	120.30
1	N	414	ARG	NE-CZ-NH1	6.89	123.74	120.30
1	R	414	ARG	NE-CZ-NH1	6.82	123.71	120.30
1	C	368	ARG	NE-CZ-NH2	-6.75	116.93	120.30
1	B	368	ARG	NE-CZ-NH2	-6.74	116.93	120.30
1	E	368	ARG	NE-CZ-NH2	-6.72	116.94	120.30
1	Q	414	ARG	NE-CZ-NH1	6.71	123.65	120.30
1	O	414	ARG	NE-CZ-NH1	6.71	123.65	120.30
1	I	349	ARG	NE-CZ-NH2	-6.69	116.95	120.30
1	D	368	ARG	NE-CZ-NH2	-6.69	116.95	120.30

Continued on next page...

Not For Manuscript Review

Continued from previous page...

Mol	Chain	Res	Type	Atoms	Z	Observed(°)	Ideal(°)
1	M	414	ARG	NE-CZ-NH1	6.69	123.64	120.30
1	H	349	ARG	NE-CZ-NH2	-6.68	116.96	120.30
1	J	349	ARG	NE-CZ-NH2	-6.67	116.96	120.30
1	A	368	ARG	NE-CZ-NH2	-6.66	116.97	120.30
1	K	349	ARG	NE-CZ-NH2	-6.66	116.97	120.30
1	F	368	ARG	NE-CZ-NH2	-6.62	116.99	120.30
1	G	349	ARG	NE-CZ-NH2	-6.58	117.01	120.30
1	L	349	ARG	NE-CZ-NH2	-6.56	117.02	120.30
1	X	250	ARG	NE-CZ-NH2	-6.52	117.04	120.30
1	L	276	ARG	NE-CZ-NH2	-6.47	117.06	120.30
1	W	250	ARG	NE-CZ-NH2	-6.47	117.07	120.30
1	J	276	ARG	NE-CZ-NH2	-6.45	117.08	120.30
1	U	365	ASP	CB-CG-OD1	6.43	124.09	118.30
1	I	276	ARG	NE-CZ-NH2	-6.42	117.09	120.30
1	X	365	ASP	CB-CG-OD1	6.40	124.06	118.30
2	m	70	ARG	NE-CZ-NH2	-6.39	117.10	120.30
1	T	365	ASP	CB-CG-OD1	6.39	124.05	118.30
1	T	250	ARG	NE-CZ-NH2	-6.39	117.10	120.30
2	p	70	ARG	NE-CZ-NH2	-6.38	117.11	120.30
1	V	365	ASP	CB-CG-OD1	6.38	124.04	118.30
1	W	365	ASP	CB-CG-OD1	6.38	124.04	118.30
1	S	365	ASP	CB-CG-OD1	6.37	124.03	118.30
1	S	250	ARG	NE-CZ-NH2	-6.36	117.12	120.30
1	U	250	ARG	NE-CZ-NH2	-6.35	117.12	120.30
1	G	276	ARG	NE-CZ-NH2	-6.34	117.13	120.30
1	K	276	ARG	NE-CZ-NH2	-6.33	117.13	120.30
2	o	70	ARG	NE-CZ-NH2	-6.30	117.15	120.30
1	V	250	ARG	NE-CZ-NH2	-6.29	117.15	120.30
1	H	276	ARG	NE-CZ-NH2	-6.29	117.16	120.30
2	q	70	ARG	NE-CZ-NH2	-6.28	117.16	120.30
2	r	70	ARG	NE-CZ-NH2	-6.26	117.17	120.30
2	n	70	ARG	NE-CZ-NH2	-6.24	117.18	120.30
1	A	391	ARG	NE-CZ-NH2	-6.09	117.25	120.30
1	J	330	TYR	CB-CG-CD2	-6.07	117.36	121.00
2	m	41	ARG	NE-CZ-NH1	6.04	123.32	120.30
1	H	330	TYR	CB-CG-CD2	-6.03	117.39	121.00
2	r	41	ARG	NE-CZ-NH1	6.02	123.31	120.30
1	B	391	ARG	NE-CZ-NH2	-6.01	117.29	120.30
1	K	438	TYR	CB-CG-CD2	-6.01	117.40	121.00
1	E	391	ARG	NE-CZ-NH2	-6.01	117.30	120.30
1	H	438	TYR	CB-CG-CD2	-5.99	117.41	121.00
1	L	330	TYR	CB-CG-CD2	-5.99	117.41	121.00

Continued on next page...

Not For Manuscript Review

Continued from previous page...

Mol	Chain	Res	Type	Atoms	Z	Observed(°)	Ideal(°)
1	I	330	TYR	CB-CG-CD2	-5.98	117.41	121.00
1	K	330	TYR	CB-CG-CD2	-5.97	117.42	121.00
1	G	330	TYR	CB-CG-CD2	-5.96	117.42	121.00
2	q	41	ARG	NE-CZ-NH1	5.95	123.28	120.30
1	J	438	TYR	CB-CG-CD2	-5.95	117.43	121.00
1	C	391	ARG	NE-CZ-NH2	-5.94	117.33	120.30
1	I	438	TYR	CB-CG-CD2	-5.93	117.44	121.00
1	L	438	TYR	CB-CG-CD2	-5.93	117.44	121.00
1	G	438	TYR	CB-CG-CD2	-5.92	117.44	121.00
1	G	89	ARG	NE-CZ-NH2	-5.92	117.34	120.30
2	p	41	ARG	NE-CZ-NH1	5.91	123.26	120.30
1	K	89	ARG	NE-CZ-NH2	-5.91	117.34	120.30
1	H	89	ARG	NE-CZ-NH2	-5.91	117.35	120.30
1	J	89	ARG	NE-CZ-NH2	-5.90	117.35	120.30
1	D	391	ARG	NE-CZ-NH2	-5.89	117.35	120.30
1	I	89	ARG	NE-CZ-NH2	-5.88	117.36	120.30
1	J	363	ARG	NE-CZ-NH2	-5.88	117.36	120.30
1	F	391	ARG	NE-CZ-NH2	-5.88	117.36	120.30
1	G	363	ARG	NE-CZ-NH2	-5.87	117.37	120.30
2	n	41	ARG	NE-CZ-NH1	5.85	123.23	120.30
1	L	89	ARG	NE-CZ-NH2	-5.85	117.38	120.30
1	L	363	ARG	NE-CZ-NH2	-5.84	117.38	120.30
1	H	363	ARG	NE-CZ-NH2	-5.84	117.38	120.30
2	o	41	ARG	NE-CZ-NH1	5.82	123.21	120.30
1	K	363	ARG	NE-CZ-NH2	-5.81	117.39	120.30
1	I	363	ARG	NE-CZ-NH2	-5.75	117.42	120.30
1	A	4	TYR	CB-CG-CD2	-5.62	117.63	121.00
1	L	301	ARG	NE-CZ-NH1	5.56	123.08	120.30
1	B	4	TYR	CB-CG-CD2	-5.53	117.68	121.00
1	D	4	TYR	CB-CG-CD2	-5.53	117.68	121.00
1	F	4	TYR	CB-CG-CD2	-5.52	117.69	121.00
1	E	4	TYR	CB-CG-CD2	-5.51	117.69	121.00
1	C	4	TYR	CB-CG-CD2	-5.50	117.70	121.00
1	G	301	ARG	NE-CZ-NH1	5.48	123.04	120.30
1	T	301	ARG	NE-CZ-NH2	-5.46	117.57	120.30
1	K	301	ARG	NE-CZ-NH1	5.46	123.03	120.30
1	I	301	ARG	NE-CZ-NH1	5.46	123.03	120.30
1	P	335	TYR	CB-CG-CD2	-5.44	117.73	121.00
1	J	301	ARG	NE-CZ-NH1	5.43	123.02	120.30
1	C	464	ARG	NE-CZ-NH2	-5.41	117.59	120.30
1	F	464	ARG	NE-CZ-NH2	-5.40	117.60	120.30
1	V	301	ARG	NE-CZ-NH2	-5.40	117.60	120.30

Continued on next page...

Not For Manuscript Review

Continued from previous page...

Mol	Chain	Res	Type	Atoms	Z	Observed(^{o})	Ideal(^{o})
1	E	464	ARG	NE-CZ-NH2	-5.39	117.60	120.30
1	M	335	TYR	CB-CG-CD2	-5.39	117.76	121.00
1	N	335	TYR	CB-CG-CD2	-5.39	117.76	121.00
1	S	301	ARG	NE-CZ-NH2	-5.39	117.61	120.30
1	X	301	ARG	NE-CZ-NH2	-5.38	117.61	120.30
1	U	301	ARG	NE-CZ-NH2	-5.38	117.61	120.30
1	O	335	TYR	CB-CG-CD2	-5.34	117.79	121.00
1	R	335	TYR	CB-CG-CD2	-5.33	117.80	121.00
1	H	301	ARG	NE-CZ-NH1	5.32	122.96	120.30
1	A	464	ARG	NE-CZ-NH2	-5.32	117.64	120.30
1	Q	335	TYR	CB-CG-CD2	-5.29	117.83	121.00
1	B	391	ARG	NE-CZ-NH1	5.28	122.94	120.30
1	B	464	ARG	NE-CZ-NH2	-5.28	117.66	120.30
1	D	464	ARG	NE-CZ-NH2	-5.27	117.67	120.30
1	W	301	ARG	NE-CZ-NH2	-5.26	117.67	120.30
1	E	391	ARG	NE-CZ-NH1	5.25	122.92	120.30
1	D	391	ARG	NE-CZ-NH1	5.23	122.91	120.30
1	E	330	TYR	CB-CG-CD2	-5.20	117.88	121.00
1	F	330	TYR	CB-CG-CD2	-5.19	117.89	121.00
1	A	391	ARG	NE-CZ-NH1	5.19	122.90	120.30
1	C	391	ARG	NE-CZ-NH1	5.19	122.89	120.30
1	C	330	TYR	CB-CG-CD2	-5.19	117.89	121.00
1	D	330	TYR	CB-CG-CD2	-5.18	117.89	121.00
1	F	391	ARG	NE-CZ-NH1	5.17	122.89	120.30
1	R	464	ARG	NE-CZ-NH1	5.17	122.89	120.30
1	Q	464	ARG	NE-CZ-NH1	5.17	122.88	120.30
1	O	464	ARG	NE-CZ-NH1	5.15	122.88	120.30
1	B	330	TYR	CB-CG-CD2	-5.15	117.91	121.00
1	P	465	ARG	NE-CZ-NH2	-5.15	117.73	120.30
1	P	464	ARG	NE-CZ-NH1	5.14	122.87	120.30
1	M	464	ARG	NE-CZ-NH1	5.13	122.86	120.30
1	M	465	ARG	NE-CZ-NH2	-5.11	117.75	120.30
1	A	330	TYR	CB-CG-CD2	-5.11	117.94	121.00
1	O	465	ARG	NE-CZ-NH2	-5.06	117.77	120.30
1	N	464	ARG	NE-CZ-NH1	5.06	122.83	120.30
1	N	465	ARG	NE-CZ-NH2	-5.05	117.78	120.30

There are no chirality outliers.

There are no planarity outliers.

Not For Manuscript Review

Not For Manuscript Review

5.2 Too-close contacts (i)

Due to software issues we are unable to calculate clashes - this section is therefore empty.

5.3 Torsion angles (i)

5.3.1 Protein backbone (i)

In the following table, the Percentiles column shows the percent Ramachandran outliers of the chain as a percentile score with respect to all PDB entries followed by that with respect to all EM entries.

The Analysed column shows the number of residues for which the backbone conformation was analysed, and the total number of residues.

Mol	Chain	Analysed	Favoured	Allowed	Outliers	Percentiles	
1	A	365/539 (68%)	347 (95%)	17 (5%)	1 (0%)	41	75
1	B	365/539 (68%)	345 (94%)	18 (5%)	2 (0%)	29	67
1	C	365/539 (68%)	348 (95%)	16 (4%)	1 (0%)	41	75
1	D	365/539 (68%)	347 (95%)	17 (5%)	1 (0%)	41	75
1	E	365/539 (68%)	347 (95%)	18 (5%)	0	100	100
1	F	365/539 (68%)	348 (95%)	16 (4%)	1 (0%)	41	75
1	G	365/539 (68%)	340 (93%)	24 (7%)	1 (0%)	41	75
1	H	365/539 (68%)	336 (92%)	27 (7%)	2 (0%)	29	67
1	I	365/539 (68%)	341 (93%)	23 (6%)	1 (0%)	41	75
1	J	365/539 (68%)	341 (93%)	23 (6%)	1 (0%)	41	75
1	K	365/539 (68%)	342 (94%)	22 (6%)	1 (0%)	41	75
1	L	365/539 (68%)	340 (93%)	24 (7%)	1 (0%)	41	75
1	M	365/539 (68%)	341 (93%)	21 (6%)	3 (1%)	19	57
1	N	365/539 (68%)	344 (94%)	17 (5%)	4 (1%)	14	51
1	O	365/539 (68%)	341 (93%)	20 (6%)	4 (1%)	14	51
1	P	365/539 (68%)	341 (93%)	21 (6%)	3 (1%)	19	57
1	Q	365/539 (68%)	342 (94%)	20 (6%)	3 (1%)	19	57
1	R	365/539 (68%)	344 (94%)	18 (5%)	3 (1%)	19	57
1	S	365/539 (68%)	329 (90%)	35 (10%)	1 (0%)	41	75
1	T	365/539 (68%)	332 (91%)	29 (8%)	4 (1%)	14	51
1	U	365/539 (68%)	332 (91%)	27 (7%)	6 (2%)	9	44

Continued on next page...

Continued from previous page...

Mol	Chain	Analysed	Favoured	Allowed	Outliers	Percentiles	
1	V	365/539 (68%)	332 (91%)	29 (8%)	4 (1%)	14	51
1	W	365/539 (68%)	332 (91%)	28 (8%)	5 (1%)	11	46
1	X	365/539 (68%)	332 (91%)	29 (8%)	4 (1%)	14	51
2	a	147/149 (99%)	136 (92%)	10 (7%)	1 (1%)	22	60
2	b	147/149 (99%)	134 (91%)	12 (8%)	1 (1%)	22	60
2	c	147/149 (99%)	135 (92%)	11 (8%)	1 (1%)	22	60
2	d	147/149 (99%)	134 (91%)	12 (8%)	1 (1%)	22	60
2	e	147/149 (99%)	136 (92%)	11 (8%)	0	100	100
2	f	147/149 (99%)	136 (92%)	11 (8%)	0	100	100
2	g	147/149 (99%)	144 (98%)	3 (2%)	0	100	100
2	h	147/149 (99%)	144 (98%)	3 (2%)	0	100	100
2	i	147/149 (99%)	144 (98%)	3 (2%)	0	100	100
2	j	147/149 (99%)	144 (98%)	3 (2%)	0	100	100
2	k	147/149 (99%)	144 (98%)	3 (2%)	0	100	100
2	l	147/149 (99%)	144 (98%)	3 (2%)	0	100	100
2	m	147/149 (99%)	140 (95%)	7 (5%)	0	100	100
2	n	147/149 (99%)	140 (95%)	7 (5%)	0	100	100
2	o	147/149 (99%)	140 (95%)	7 (5%)	0	100	100
2	p	147/149 (99%)	140 (95%)	7 (5%)	0	100	100
2	q	147/149 (99%)	140 (95%)	7 (5%)	0	100	100
2	r	147/149 (99%)	140 (95%)	7 (5%)	0	100	100
2	s	147/149 (99%)	143 (97%)	4 (3%)	0	100	100
2	t	147/149 (99%)	143 (97%)	4 (3%)	0	100	100
2	u	147/149 (99%)	143 (97%)	4 (3%)	0	100	100
2	v	147/149 (99%)	143 (97%)	4 (3%)	0	100	100
2	w	147/149 (99%)	143 (97%)	4 (3%)	0	100	100
2	x	147/149 (99%)	143 (97%)	4 (3%)	0	100	100
All	All	12288/16512 (74%)	11537 (94%)	690 (6%)	61 (0%)	32	67

All (61) Ramachandran outliers are listed below:

Mol	Chain	Res	Type
1	V	4	TYR

Continued on next page...

Not For Manuscript Review

Continued from previous page...

Mol	Chain	Res	Type
1	P	515	ALA
1	W	4	TYR
1	Q	515	ALA
1	X	4	TYR
1	R	515	ALA
1	M	515	ALA
1	T	4	TYR
1	N	515	ALA
1	U	4	TYR
1	O	515	ALA
1	P	514	ILE
1	Q	514	ILE
1	R	514	ILE
1	M	514	ILE
1	N	514	ILE
1	B	83	SER
1	O	514	ILE
1	P	271	MET
1	M	271	MET
2	a	116	ASN
2	b	116	ASN
1	O	271	MET
2	c	117	ALA
1	V	237	HIS
1	D	451	VAL
1	W	237	HIS
1	X	237	HIS
1	F	451	VAL
1	A	451	VAL
1	T	237	HIS
1	N	271	MET
1	B	451	VAL
1	U	237	HIS
1	C	451	VAL
1	J	451	VAL
2	d	114	LEU
1	K	451	VAL
1	L	451	VAL
1	G	451	VAL
1	H	451	VAL
1	U	281	LEU
1	O	505	LEU

Continued on next page...

Not For Manuscript Review

Continued from previous page...

Mol	Chain	Res	Type
1	I	451	VAL
1	W	504	ASP
1	H	253	PHE
1	U	504	ASP
1	V	451	VAL
1	W	451	VAL
1	X	451	VAL
1	S	451	VAL
1	T	451	VAL
1	U	451	VAL
1	V	514	ILE
1	W	514	ILE
1	X	514	ILE
1	T	514	ILE
1	U	514	ILE
1	R	90	VAL
1	N	90	VAL
1	Q	90	VAL

5.3.2 Protein sidechains (i)

In the following table, the Percentiles column shows the percent sidechain outliers of the chain as a percentile score with respect to all PDB entries followed by that with respect to all EM entries.

The Analysed column shows the number of residues for which the sidechain conformation was analysed, and the total number of residues.

Mol	Chain	Analysed	Rotameric	Outliers	Percentiles	
1	A	295/416 (71%)	295 (100%)	0	100	100
1	B	295/416 (71%)	294 (100%)	1 (0%)	92	95
1	C	295/416 (71%)	295 (100%)	0	100	100
1	D	295/416 (71%)	295 (100%)	0	100	100
1	E	295/416 (71%)	293 (99%)	2 (1%)	84	90
1	F	295/416 (71%)	295 (100%)	0	100	100
1	G	295/416 (71%)	295 (100%)	0	100	100
1	H	295/416 (71%)	295 (100%)	0	100	100
1	I	295/416 (71%)	295 (100%)	0	100	100
1	J	295/416 (71%)	295 (100%)	0	100	100

Continued on next page...

Not For Manuscript Review

Continued from previous page...

Mol	Chain	Analysed	Rotameric	Outliers	Percentiles	
1	K	295/416 (71%)	295 (100%)	0	100	100
1	L	295/416 (71%)	295 (100%)	0	100	100
1	M	295/416 (71%)	291 (99%)	4 (1%)	67	81
1	N	295/416 (71%)	292 (99%)	3 (1%)	76	86
1	O	295/416 (71%)	292 (99%)	3 (1%)	76	86
1	P	295/416 (71%)	291 (99%)	4 (1%)	67	81
1	Q	295/416 (71%)	292 (99%)	3 (1%)	76	86
1	R	295/416 (71%)	290 (98%)	5 (2%)	60	78
1	S	295/416 (71%)	292 (99%)	3 (1%)	76	86
1	T	295/416 (71%)	293 (99%)	2 (1%)	84	90
1	U	295/416 (71%)	293 (99%)	2 (1%)	84	90
1	V	295/416 (71%)	293 (99%)	2 (1%)	84	90
1	W	295/416 (71%)	293 (99%)	2 (1%)	84	90
1	X	295/416 (71%)	293 (99%)	2 (1%)	84	90
2	a	129/129 (100%)	129 (100%)	0	100	100
2	b	129/129 (100%)	128 (99%)	1 (1%)	81	89
2	c	129/129 (100%)	129 (100%)	0	100	100
2	d	129/129 (100%)	129 (100%)	0	100	100
2	e	129/129 (100%)	129 (100%)	0	100	100
2	f	129/129 (100%)	129 (100%)	0	100	100
2	g	129/129 (100%)	129 (100%)	0	100	100
2	h	129/129 (100%)	129 (100%)	0	100	100
2	i	129/129 (100%)	129 (100%)	0	100	100
2	j	129/129 (100%)	129 (100%)	0	100	100
2	k	129/129 (100%)	129 (100%)	0	100	100
2	l	129/129 (100%)	129 (100%)	0	100	100
2	m	129/129 (100%)	129 (100%)	0	100	100
2	n	129/129 (100%)	129 (100%)	0	100	100
2	o	129/129 (100%)	128 (99%)	1 (1%)	81	89
2	p	129/129 (100%)	128 (99%)	1 (1%)	81	89
2	q	129/129 (100%)	128 (99%)	1 (1%)	81	89

Continued on next page...

Not For Manuscript Review

Continued from previous page...

Mol	Chain	Analysed	Rotameric	Outliers	Percentiles	
2	r	129/129 (100%)	128 (99%)	1 (1%)	81	89
2	s	129/129 (100%)	129 (100%)	0	100	100
2	t	129/129 (100%)	129 (100%)	0	100	100
2	u	129/129 (100%)	129 (100%)	0	100	100
2	v	129/129 (100%)	129 (100%)	0	100	100
2	w	129/129 (100%)	129 (100%)	0	100	100
2	x	129/129 (100%)	129 (100%)	0	100	100
All	All	10176/13080 (78%)	10133 (100%)	43 (0%)	91	94

All (43) residues with a non-rotameric sidechain are listed below:

Mol	Chain	Res	Type
1	V	320	ARG
1	V	451	VAL
1	P	89	ARG
1	P	269	ASP
1	P	271	MET
1	P	507	ARG
2	p	94	ARG
1	W	320	ARG
1	W	451	VAL
1	Q	269	ASP
1	Q	270	LEU
1	Q	453	ASN
2	q	94	ARG
1	E	452	PHE
1	E	505	LEU
1	X	320	ARG
1	X	451	VAL
1	R	89	ARG
1	R	269	ASP
1	R	270	LEU
1	R	274	TYR
1	R	453	ASN
2	r	94	ARG
1	S	237	HIS
1	S	320	ARG
1	S	451	VAL
1	M	269	ASP
1	M	271	MET

Continued on next page...

Not For Manuscript Review

Continued from previous page...

Mol	Chain	Res	Type
1	M	453	ASN
1	M	507	ARG
1	T	320	ARG
1	T	451	VAL
1	N	269	ASP
1	N	271	MET
1	N	453	ASN
1	B	507	ARG
2	b	115	ARG
1	U	320	ARG
1	U	451	VAL
1	O	269	ASP
1	O	271	MET
1	O	453	ASN
2	o	94	ARG

Sometimes sidechains can be flipped to improve hydrogen bonding and reduce clashes. All (54) such sidechains are listed below:

Mol	Chain	Res	Type
1	V	72	HIS
1	V	80	ASN
2	v	116	ASN
1	J	294	HIS
2	j	67	GLN
1	D	311	GLN
2	d	14	ASN
2	d	53	ASN
2	d	116	ASN
1	W	72	HIS
2	w	116	ASN
1	K	294	HIS
1	K	356	HIS
1	E	311	GLN
1	E	455	ASN
2	e	14	ASN
2	e	53	ASN
1	X	72	HIS
1	X	80	ASN
2	x	116	ASN
1	R	371	HIS
1	L	294	HIS
2	l	67	GLN

Continued on next page...

Not For Manuscript Review

Continued from previous page...

Mol	Chain	Res	Type
1	F	311	GLN
2	f	14	ASN
2	f	53	ASN
1	S	72	HIS
2	s	116	ASN
1	M	371	HIS
1	G	294	HIS
1	G	356	HIS
2	g	67	GLN
1	A	311	GLN
2	a	14	ASN
2	a	53	ASN
1	T	72	HIS
1	T	80	ASN
2	t	116	ASN
1	N	371	HIS
1	B	80	ASN
1	B	311	GLN
2	b	14	ASN
2	b	53	ASN
2	b	116	ASN
1	U	72	HIS
1	U	80	ASN
2	u	116	ASN
1	O	371	HIS
1	I	294	HIS
1	I	356	HIS
1	C	311	GLN
2	c	14	ASN
2	c	53	ASN
2	c	116	ASN

5.3.3 RNA (i)

There are no RNA molecules in this entry.

5.4 Non-standard residues in protein, DNA, RNA chains (i)

There are no non-standard protein/DNA/RNA residues in this entry.

Not For Manuscript Review

5.5 Carbohydrates (i)

There are no monosaccharides in this entry.

5.6 Ligand geometry (i)

There are no ligands in this entry.

5.7 Other polymers (i)

There are no such residues in this entry.

5.8 Polymer linkage issues (i)

There are no chain breaks in this entry.

Not For Manuscript Review

6 Map visualisation (i)

This section contains visualisations of the EMDB entry D_1292126605. These allow visual inspection of the internal detail of the map and identification of artifacts.

Images derived from a raw map, generated by summing the deposited half-maps, are presented below the corresponding image components of the primary map to allow further visual inspection and comparison with those of the primary map.

6.1 Orthogonal projections (i)

6.1.1 Primary map

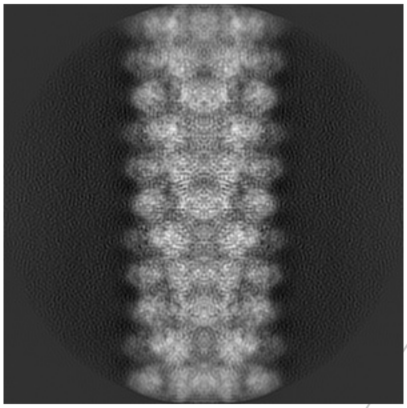

X

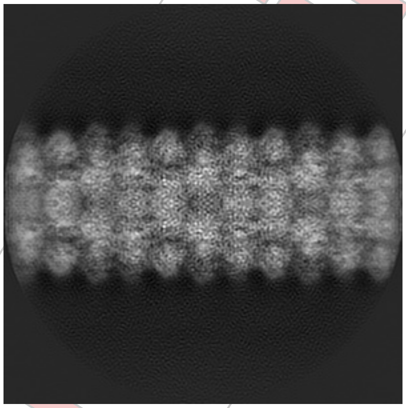

Y

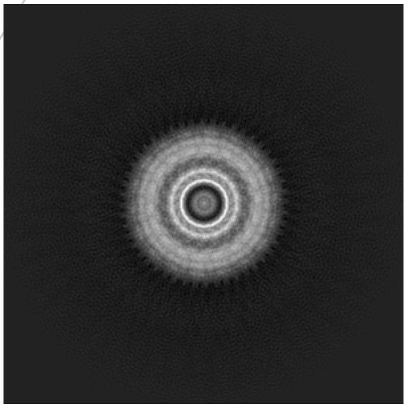

Z

6.1.2 Raw map

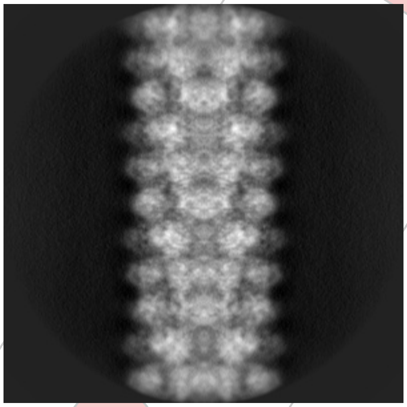

X

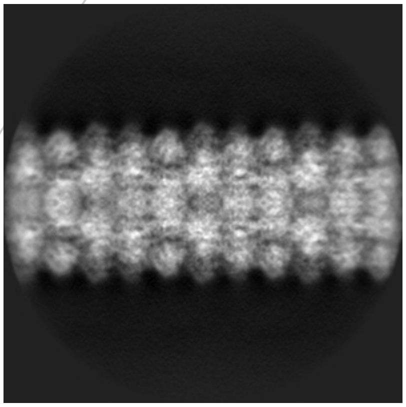

Y

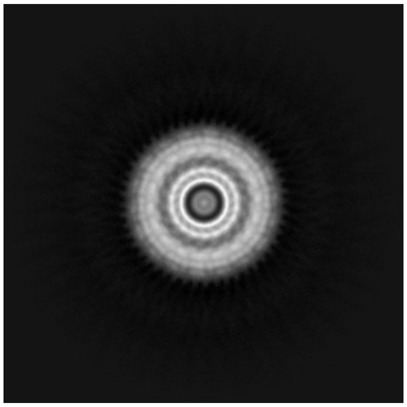

Z

The images above show the map projected in three orthogonal directions.

Not For Manuscript Review

6.2 Central slices (i)

6.2.1 Primary map

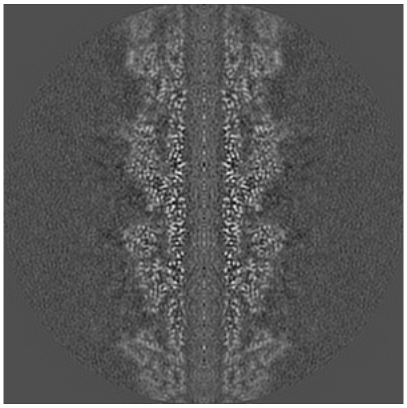

X Index: 160

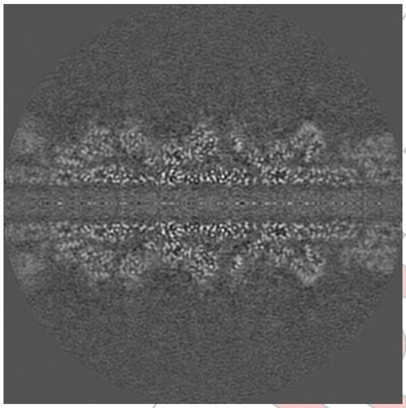

Y Index: 160

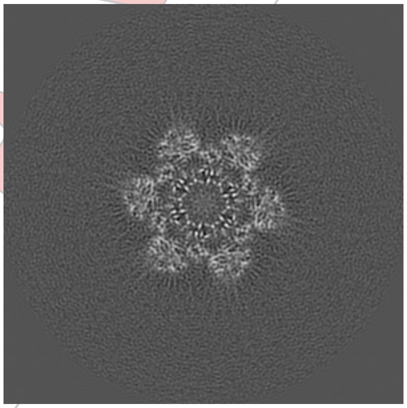

Z Index: 160

6.2.2 Raw map

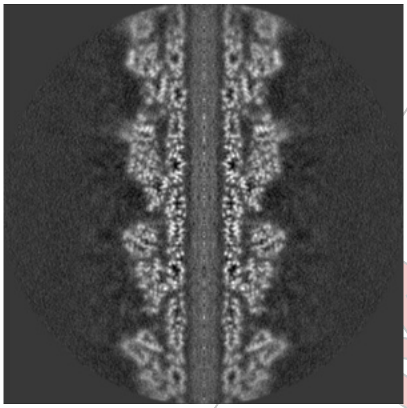

X Index: 160

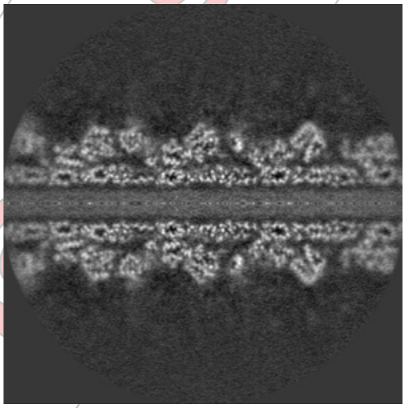

Y Index: 160

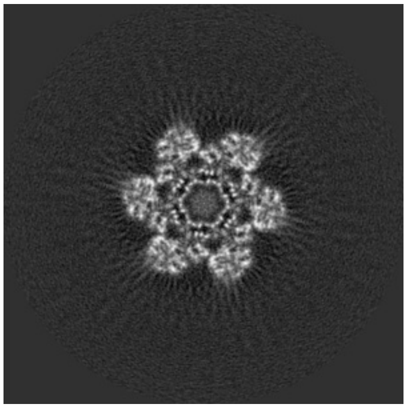

Z Index: 160

The images above show central slices of the map in three orthogonal directions.

Not For Manuscript Review

6.3 Largest variance slices (i)

6.3.1 Primary map

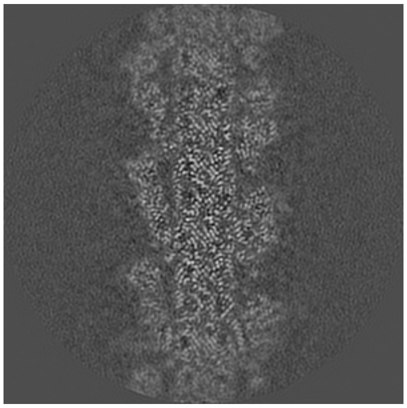

X Index: 143

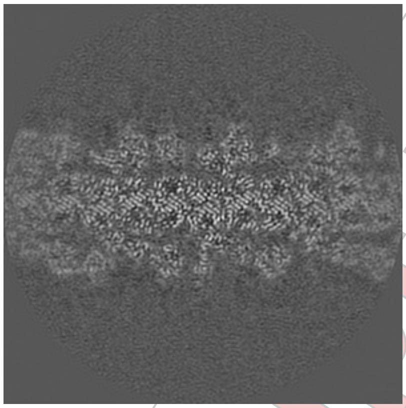

Y Index: 178

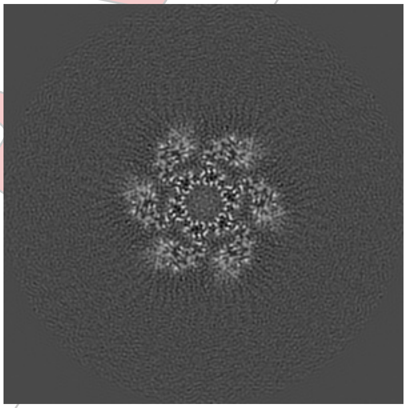

Z Index: 163

6.3.2 Raw map

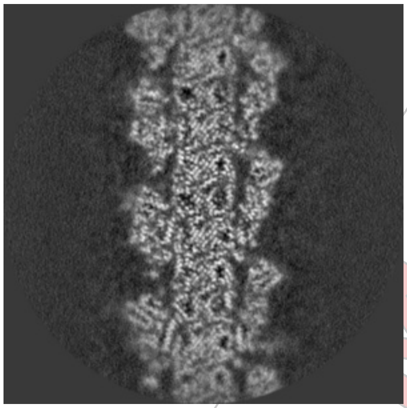

X Index: 177

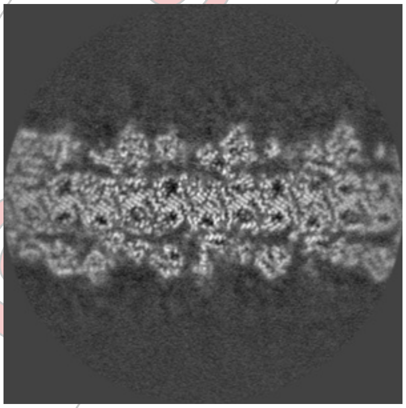

Y Index: 178

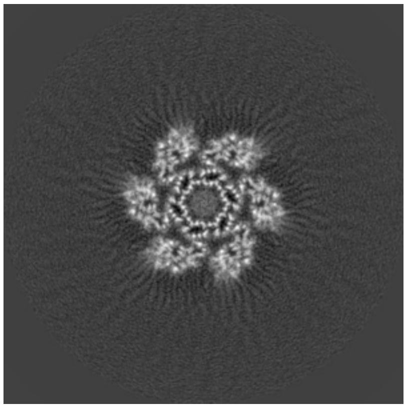

Z Index: 163

The images above show the largest variance slices of the map in three orthogonal directions.

Not For Manuscript Review

6.4 Orthogonal surface views (i)

6.4.1 Primary map

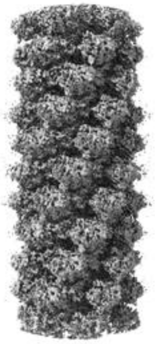

X

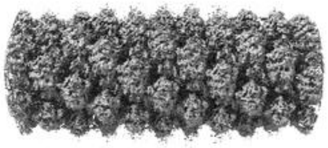

Y

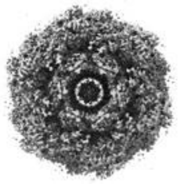

Z

The images above show the 3D surface view of the map at the recommended contour level 0.03. These images, in conjunction with the slice images, may facilitate assessment of whether an appropriate contour level has been provided.

6.4.2 Raw map

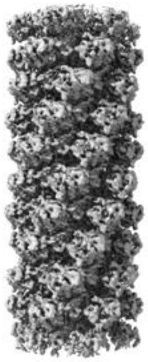

X

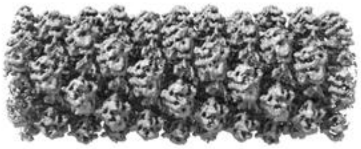

Y

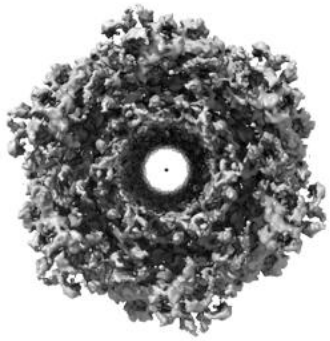

Z

These images show the 3D surface of the raw map. The raw map's contour level was selected so that its surface encloses the same volume as the primary map does at its recommended contour level.

6.5 Mask visualisation (i)

This section was not generated. No masks/segmentation were deposited.

Not For Manuscript Review

7 Map analysis (i)

This section contains the results of statistical analysis of the map.

7.1 Map-value distribution (i)

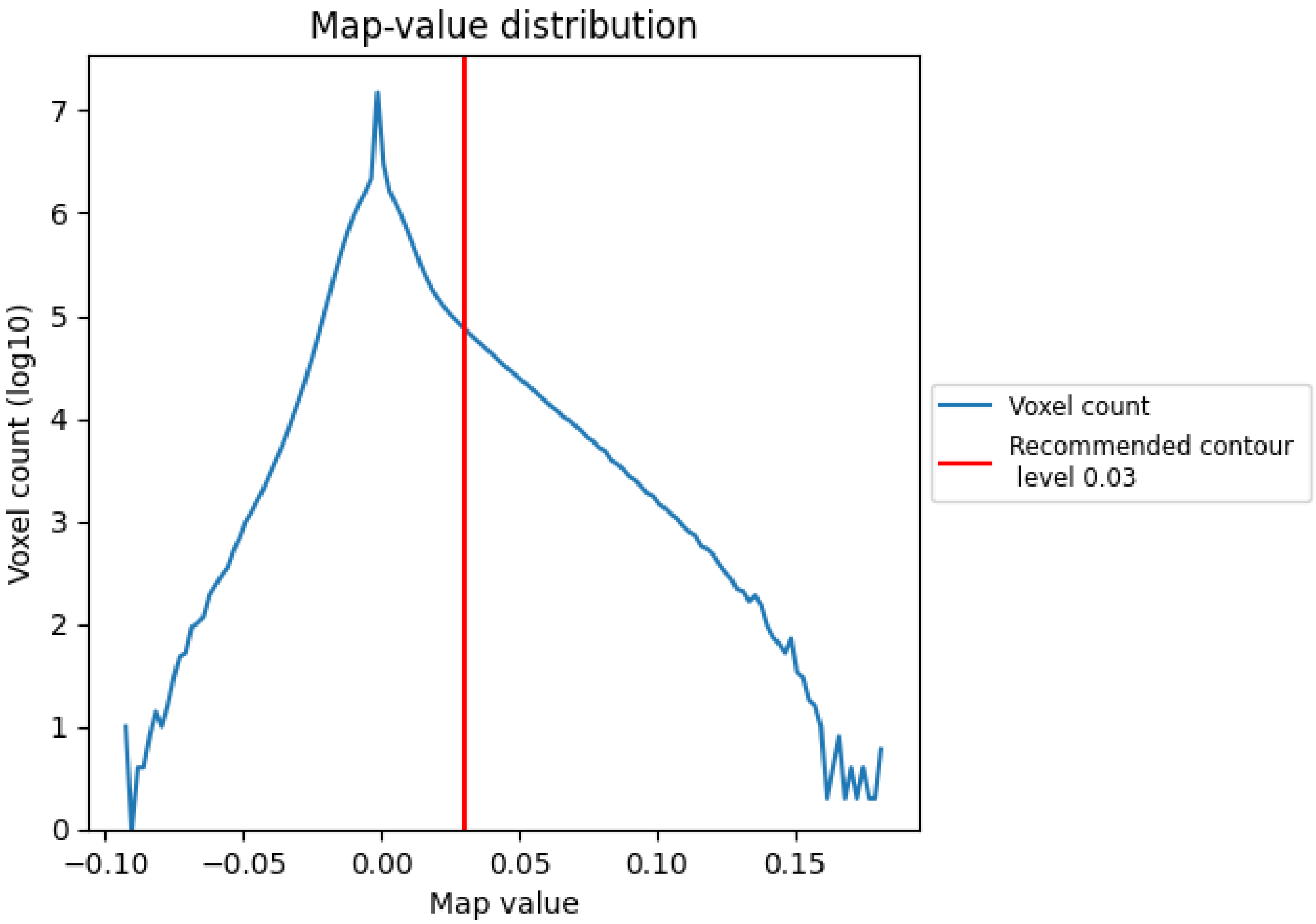

The map-value distribution is plotted in 128 intervals along the x-axis. The y-axis is logarithmic. A spike in this graph at zero usually indicates that the volume has been masked.

Not For Review

7.2 Volume estimate (i)

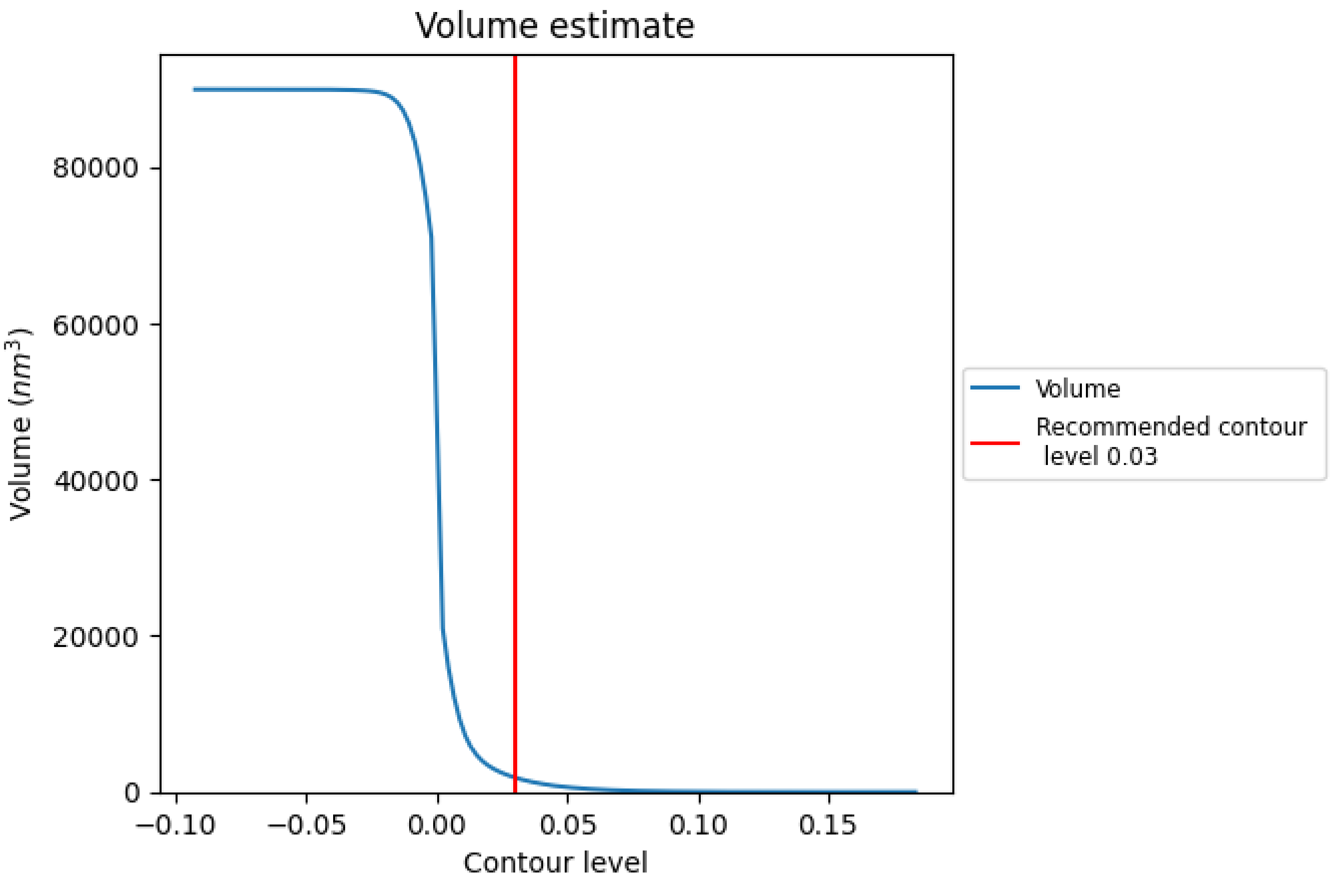

The volume at the recommended contour level is 1828 nm^3; this corresponds to an approximate mass of 1651 kDa.

The volume estimate graph shows how the enclosed volume varies with the contour level. The recommended contour level is shown as a vertical line and the intersection between the line and the curve gives the volume of the enclosed surface at the given level.

Not For Manuscript Review

7.3 Rotationally averaged power spectrum ⓘ

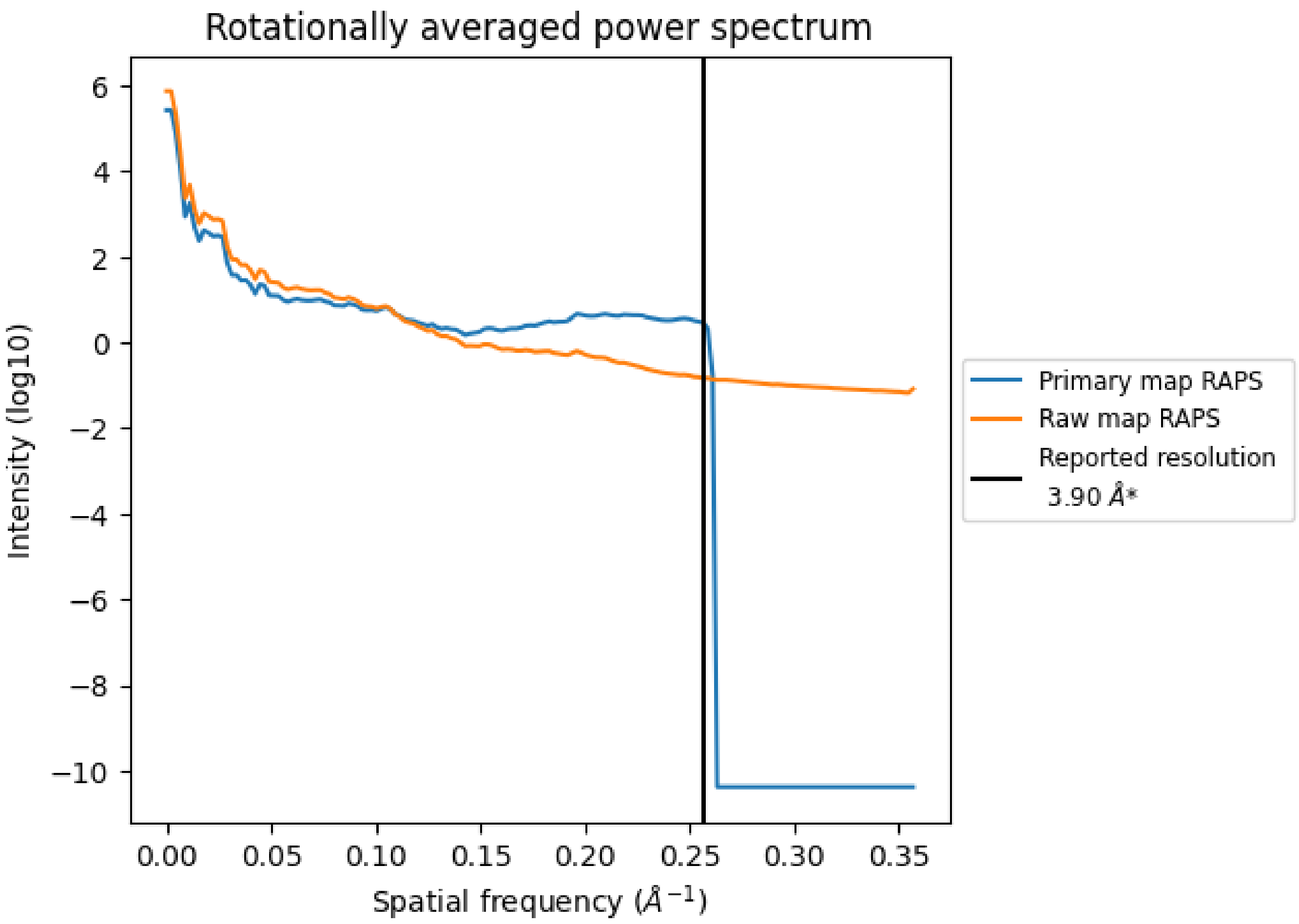

*Reported resolution corresponds to spatial frequency of 0.256 $Å^{-1}$

Not For Manuscript Review

8 Fourier-Shell correlation (i)

Fourier-Shell Correlation (FSC) is the most commonly used method to estimate the resolution of single-particle and subtomogram-averaged maps. The shape of the curve depends on the imposed symmetry, mask and whether or not the two 3D reconstructions used were processed from a common reference. The reported resolution is shown as a black line. A curve is displayed for the half-bit criterion in addition to lines showing the 0.143 gold standard cut-off and 0.5 cut-off.

8.1 FSC (i)

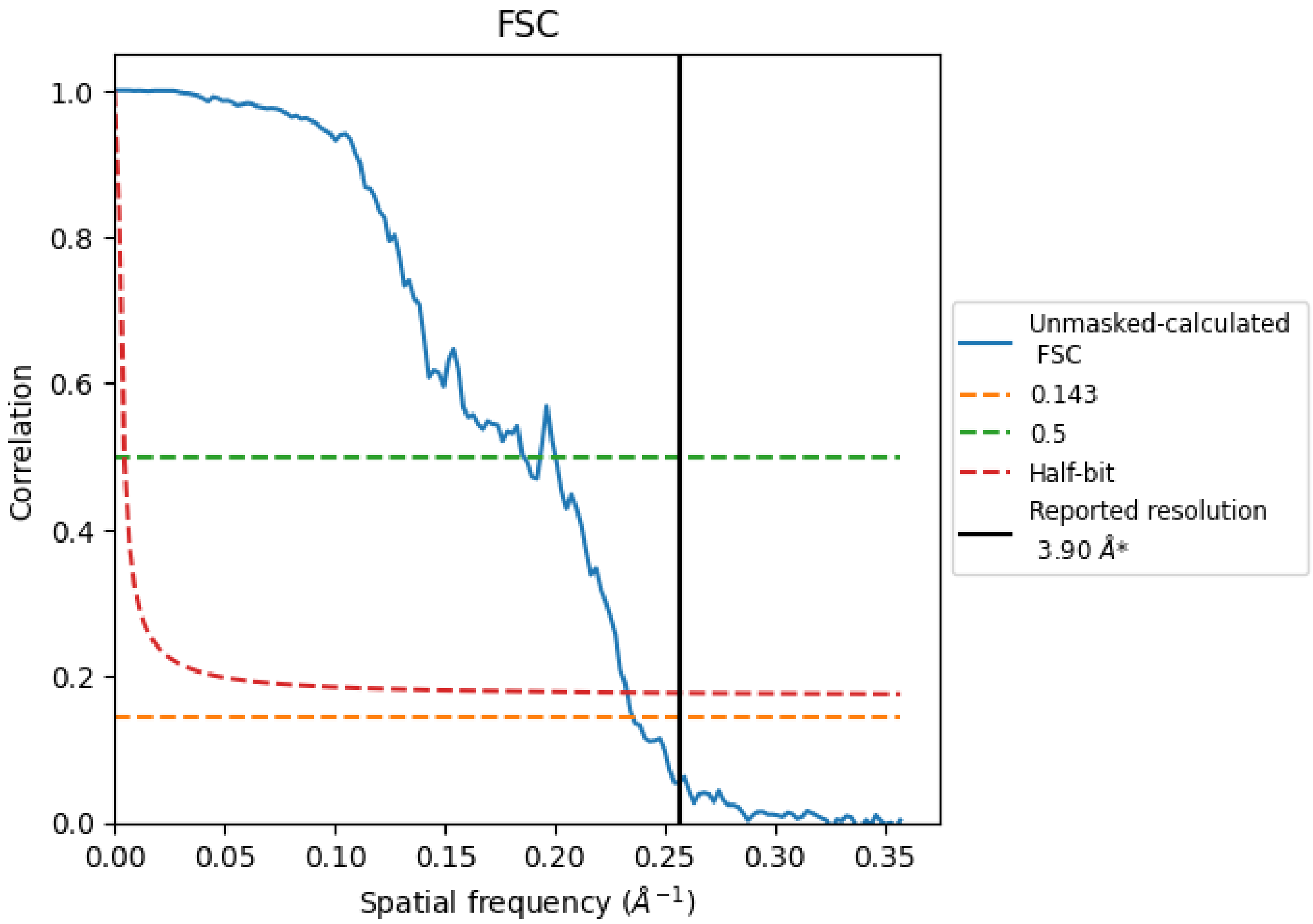

*Reported resolution corresponds to spatial frequency of 0.256 Å^{-1}

Not For Manuscript Review

8.2 Resolution estimates (i)

Resolution estimate (Å)	Estimation criterion (FSC cut-off)		
	0.143	0.5	Half-bit
Reported by author	3.90	-	-
Author-provided FSC curve	-	-	-
Unmasked-calculated*	4.24	5.39	4.29

*Resolution estimate based on FSC curve calculated by comparison of deposited half-maps.

Not For Manuscript Review

9 Map-model fit (i)

This section contains information regarding the fit between EMDB map D_1292126605 and PDB model D_1292126605. Per-residue inclusion information can be found in section 3 on page 8.

9.1 Map-model overlay (i)

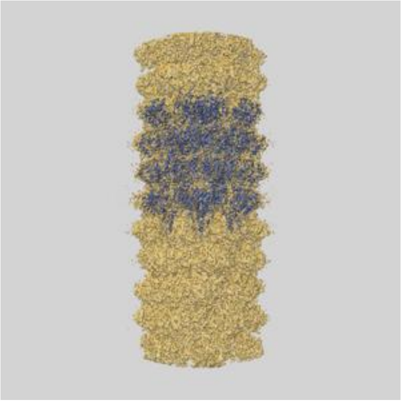

X

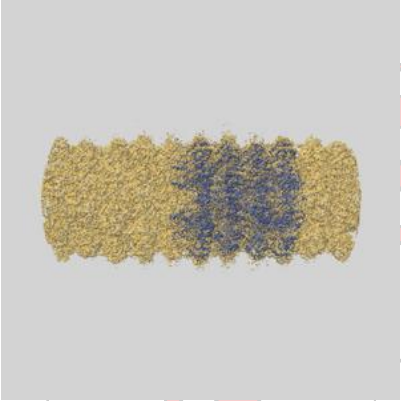

Y

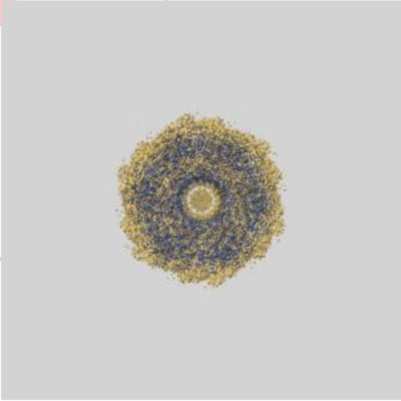

Z

The images above show the 3D surface view of the map at the recommended contour level 0.03 at 50% transparency in yellow overlaid with a ribbon representation of the model coloured in blue. These images allow for the visual assessment of the quality of fit between the atomic model and the map.

Not For Manuscript Review

9.2 Q-score mapped to coordinate model (i)

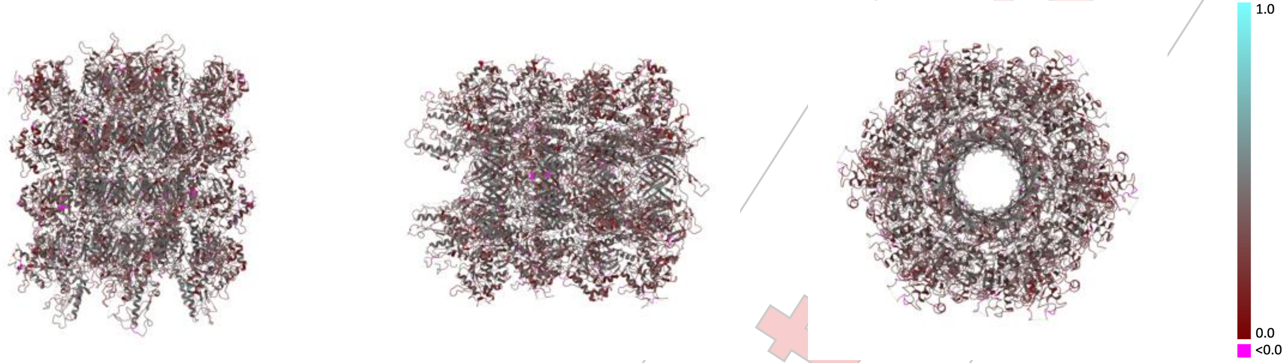

X Y Z

The images above show the model with each residue coloured according its Q-score. This shows their resolvability in the map with higher Q-score values reflecting better resolvability. Please note: Q-score is calculating the resolvability of atoms, and thus high values are only expected at resolutions at which atoms can be resolved. Low Q-score values may therefore be expected for many entries.

9.3 Atom inclusion mapped to coordinate model (i)

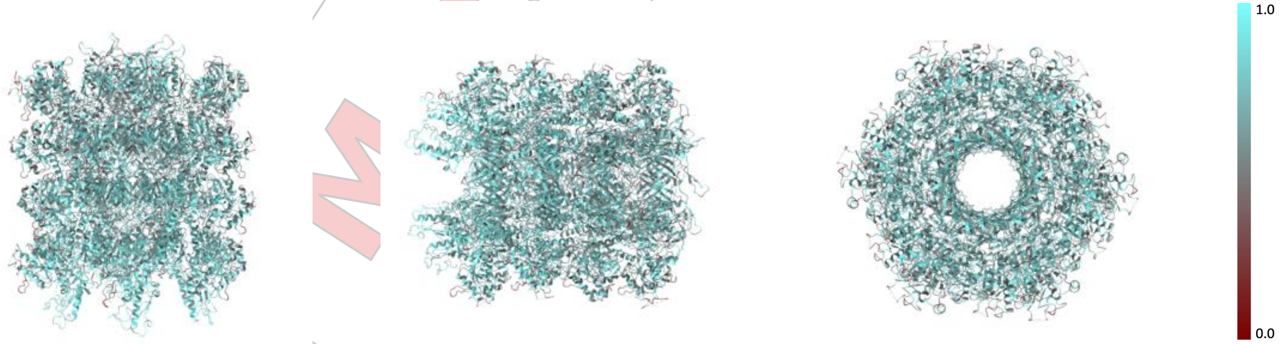

X Y Z

The images above show the model with each residue coloured according to its atom inclusion. This shows to what extent they are inside the map at the recommended contour level (0.03).

Not For Manuscript Review

9.4 Atom inclusion (i)

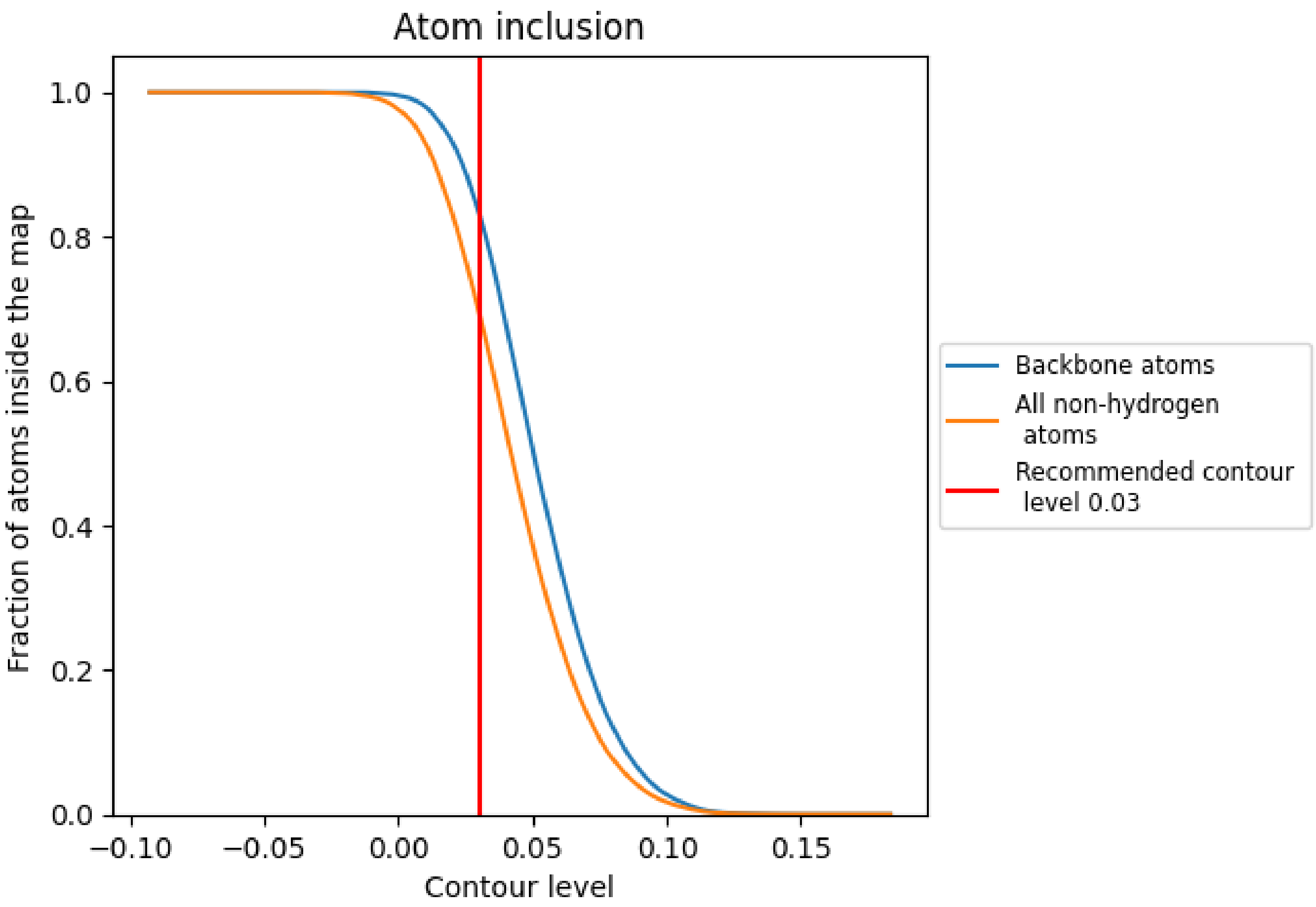

At the recommended contour level, 83% of all backbone atoms, 70% of all non-hydrogen atoms, are inside the map.

Not For Manuscript Review

9.5 Map-model fit summary (i)

The table lists the average atom inclusion at the recommended contour level (0.03) and Q-score for the entire model and for each chain.

Chain	Atom inclusion	Q-score
All	0.6971	0.3370
A	0.7313	0.3460
B	0.7320	0.3400
C	0.7384	0.3440
D	0.7324	0.3460
E	0.7277	0.3400
F	0.7417	0.3460
G	0.7134	0.3290
H	0.7159	0.3260
I	0.7173	0.3300
J	0.7152	0.3310
K	0.7184	0.3270
L	0.7169	0.3270
M	0.6933	0.3210
N	0.6929	0.3230
O	0.6915	0.3210
P	0.6904	0.3210
Q	0.6912	0.3230
R	0.6926	0.3230
S	0.6474	0.3060
T	0.6553	0.3100
U	0.6535	0.3120
V	0.6510	0.3130
W	0.6496	0.3130
X	0.6517	0.3120
a	0.7274	0.3790
b	0.7159	0.3760
c	0.7239	0.3770
d	0.7319	0.3780
e	0.7301	0.3780
f	0.7336	0.3760
g	0.7124	0.3640
h	0.7124	0.3660
i	0.7204	0.3640
j	0.7133	0.3670

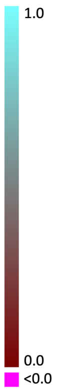

Continued on next page...

Not For Manuscript Review

Continued from previous page...

Chain	Atom inclusion	Q-score
k	0.7168	0.3670
l	0.7168	0.3640
m	0.6708	0.3630
n	0.6752	0.3630
o	0.6717	0.3600
p	0.6726	0.3600
q	0.6779	0.3650
r	0.6726	0.3620
s	0.6646	0.3560
t	0.6593	0.3530
u	0.6593	0.3540
v	0.6593	0.3550
w	0.6575	0.3560
x	0.6611	0.3550

Not For Manuscript Review
