# Supplementary material for: Cytoplasmic contractile injection systems mediate cell death in Streptomyces
Source: Nat Microbiol. 2023 Mar 9;8(4):711–26. doi: 10.1038/s41564-023-01341-x (PMC10066040; doi:10.1038/s41564-023-01341-x)
Supplement: Supplementary file 3 — Peer Review File [file 41564_2023_1341_MOESM3_ESM.doc]

Peer Review Information

**Journal: Nature Microbiology**

**Manuscript Title:** Cytoplasmic contractile injection systems mediate cell death in Streptomyces

**Corresponding author name(s): Professor Martin Pilhofer**

**Reviewer Comments & Decisions:**

***EA, duplicate for each version as needed, and then delete this instruction.***

**Decision Letter, initial version:**

| **Message:** | 16th September 2022  Dear Martin,  Thank you for your patience while your manuscript "Cytoplasmic contractile injection systems mediate cell death in Streptomyces" was under peer review at Nature Microbiology. It has now been seen by our referees, whose expertise and comments you will find at the end of this email. In the light of their advice, we have decided that unfortunately we cannot offer to publish your manuscript in Nature Microbiology.  From the reports, you will see that while they find your work of some potential interest, the referees raise concerns about the insufficient level of mechanistic/biological insights provided, and about the strength of the novel conclusions that can be drawn at this stage. In particular, referee #1 mentions that “The exact mechanism of action of CISs in Streptomyces remains somewhat unclear”. Referee #2 also states that a MOA would make the study stronger. Referee #4 feels that additional experimental work is required to exclude that the observed decrease in GFP signal could be potentially also explained by decreased protein synthesis (or poor GFP folding) rather than cell death/lysis. Given the insufficient mechanistic insights, and the limited insight into the relevance of the findings for Streptomyces biology, unfortunately, we cannot further consider your manuscript at this stage.  Since you opted out of transfer consultations, we have not consulted with editors from other journals, but if you would be interested in this option, I'm happy to consult with my colleagues at Nature Communications.  I am sorry that we cannot be more positive on this occasion, but hope that you find the referees' comments helpful when preparing your paper for resubmission elsewhere.  Yours sincerely,  [redacted]  ******************* Reviewer Expertise:  Referee #1: Secretion systems; structural biology Referee #2: Cryo electron microscopy Referee #3: Bacterial structural biology; cryoEM Referee #4: Bacterial injection apparatus  Reviewers Comments:  Reviewer #1 (Remarks to the Author):  The MS “Cytoplasmic contractile injection systems mediate cell death in Streptomyces” describes a very large (huge!) body of work that includes genetic manipulation of Streptomyces coelicolor, several types of cryo-electron microscopy (cryo-electron tomography, cryo-focused ion beam specimen milling, single particle imaging, and negative staining), fluorescence microscopy, correlative cryo-fluorescence and cryo-electron microscopy, mass-spectrometry, and many molecular biology assays. It is a truly impressive MS that is a pleasure to read - a real tour de force! The exact mechanism of action of CISs in Streptomyces remains somewhat unclear, but the MS clearly demonstrates that CISs play a critical role in the development program of Streptomyces. Importantly, even though Streptomyces CISs are most similar to extracellular CISs that mediate cytotoxic activity outside of the producer cell, Streptomyces CISs appear to function inside the cell by a yet unknown mechanism.  I am very impressed with the quality of the data and presentation of the results. I have only a few comments that are aimed at the clarification of some aspects and further improvement of the presentation.  L. 218. will lead -> leads  L. 277 Conclusions -> Discussion  L. 742. Data availability. In principle, reviewers are expected to analyze validation reports, etc., so the data should have been deposited to databases prior to the submission of the paper.  Fig. 1f, 1g Please present additional tomograms that demonstrate localization of CISs in the cytoplasm (a Supplementary or Extended View figure, perhaps?).  Fig. 2c, 2f. Here and elsewhere, use a consistent orientation of the sheath subunit with respect to the sixfold - desirably, show a sideview. Panels b and e appear to show the sheath with the baseplate in the up position. Phage people draw tails with the baseplate down, so the sheath subunit is usually shown with the N- and C-terminal linker arms pointing downwards as well. So, for consistency and for reaching out to a larger audience, it might be prudent to show the sheath in that orientation. The sheath subunit in panels c and f can also be shown in a “sideway” view with the linker arms pointing downward and the tube subunit next to it.  Fig. 4: c-f panels. Do the three different colors indicate three biological repeats? e panel: the CCCP + DMSO experiment is not described in the text.   Extended Data Fig. 1c  No MW marker and no major bands identified. So, I am not sure what we are looking at (and looking for) in this figure.  ED Fig. 2c. The second line label in the sequence alignment should be 1-99, not 1-95.  ED Fig 3a. Does this panel show the contracted sheath subunit as stated in the legend? A comparison of the atomic models of the contracted and non-contractile sheath subunit is needed. Why is this mutant non-contractile? Is the topology of the the N-terminal linker arms altered in a similar way to the non-contractile T6SS sheath mutant? This needs to be explained in the text. ED Fig 4a. As the sheath subunit is not in a “standard” orientation, the location of the insertion site with respect to the overall structure of the sheath is difficult to understand. The disordered domain should also be indicated.  ED Fig 7. The scale of the first and third columns must be different despite the same size scale bar. If the scale is indeed the same, an explanation of why the structures have different apparent sizes is needed.    Reviewer #2 (Remarks to the Author):  The manuscript by Casu and colleagues reveals that the contractile injection system (CIS) in a bacterium Streptomyces modulates cell death. For this the authors identified the components of the system using sequence analysis tools, persormed structural analysis of purified contracted sheath and extended theath plus the inner tubeby single particle cryo-EM. The authors further performed cellular imaging by electron cryotomography and showed that the injections systems are not attached to the cell walls and that the fraction of contracted systems was higher in partially lysed and ghost cells. Finally the authors defined a new cellular role for the CIS in S. coelicolor - it is related to stress-related cell death and is involved in regulation of sporulation as shown by analysing the mutants with CIS knocked out or with a non-functional CIS. In conclusion the authors emphasised that the role of CIS in Streptomyces is different to the other known CISs.  The experiments are well designed and executed, the paper is well structured and written. I have several important comments: 1. The authors were succesful in obtaining high resolution structural information about the CIS by single particle cryo-EM, however all they say is that is is the same as the other systems and all they use it for is to design a non/contractile mutant and add a fluorophore. However this was probably possible to do even without knowing the structure, by just looking at the sequence of an Alphafold2 predictions? Is there anything different to the model CISs? 2. I am curious if this CIS is supposed to be recycled by a mechanism similar to ClpV in T6SS? Does the genome contain a ClpV homologue? 3. However, most importantly, the authors claim to show the new functional role of CIS in Streptomyces, but the mechanism of action is unclear which makes the message rather descriptive. The authors suggest that there may be membrane- or cell wall-targeting effectors involved. If true this would have been very exciting, could the athors identify/suggest/conditionally express some of those putative effectors and show the same mechanism as the CIS? This would make the paper much more interesting.    Reviewer #3 (Remarks to the Author):  REVIEW OF NMICROBIOL-22071688  SUMMARY  In this study, Casu et al. present a comprehensive structural and functional analysis of the Streptomyces contractile injection system (CIS). This work builds upon recent findings that CISs, syringe-like protein complexes that can secrete effector cargo proteins, are much more widespread and performing different functions than previously thought. For example, type VI secretion systems (T6SSs) are anchored to the inner membrane of gram-negative bacteria and are specialized in attacking other cells. Recently reported thylakoid CISs from cyanobacteria target the cell’s own outer membrane in a type of programmed cell death. Or, what would seemingly be the mode of action here, conventional extracellular CISs (eCISs) are assembled as free-floating particles in the cytoplasm for later release into the medium, then targeting other cells. Surprisingly though, the authors here first show through bioinformatics analyses that the S. coelicolor and S. venezuelae CIS gene clusters both lack a tail fiber protein (Afp13) and a tail measure protein (Afp14), hinting that this CIS does not target other cells. Furthermore, experimental evidence is given that the Streptomyces CIS is assembled and confined to the cytoplasm, in stark contrast to other known CISs.  The authors use single particle cryo-electron microscopy (cryo-EM) to resolve the structure of the contracted S. coelicolor CIS (CISSc) at 3.6 Å resolution, the first from a gram-positive bacterium to be reported. This structure is then used to inform the engineering of a non-functional extended CISSc as well as a fluorescently tagged variant, allowing them to perform time-resolved subcellular localization by fluorescence light microscopy (fLM) as well as correlated light and electron microscopy (CLEM). Notably, the authors also did cryo-electron tomography (cryo-ET) of native cells, which showed that the level of contraction of the CISSc correlates with cellular integrity, as they reveal that clusters CISSc tend to be found closer to the membrane of damaged cells. Especially in “ghost” cells where essentially only the peptidoglycan wall is left, clusters of contracted CISSc can be observed.  In possession of the mutants and imaging tools, the study goes on to elegantly investigate the function of the Streptomyces CIS under various cellular stress conditions (+Nisin, +CCCP, +UV) as well as in the multicellular development of Streptomyces colonies and their production of secondary metabolites.  As a non-expert in the field of CISs, nor in Streptomyces, I found the study to be concisely presented and easy to follow. Complementary imaging techniques are well employed to shed light on the unusual function of a little studied protein complex in a model organism of high importance to agriculture and antibiotics production. I fully recommend the publication of this study only with minor corrections.  MAJOR COMMENTS  Given the central importance of the cryo-EM structure in the study, the experimental densities with fitted models should be shown in a main figure. Currently they are only shown zoomed out in the local resolution plots in Extended Data Figs. 2 and 3, making it difficult to assess the quality of the map and the respective model, considering that this resolution range is particularly tricky to model. For example, a figure similar to Ext. Data. Fig. 3a with the EM density instead of a surface model could be shown, for the WT and the engineered non-contractile mutant.  Furthermore, no “Table 1” information was provided with details on the map and model statistics.  On the biological aspects of the work, my comments are curiosities of a non-expert that do not hinder publication of the study, and authors should address them at their discretion:  While it is conceivable, in light of the evidence presented, that CISSc are recruited to the membrane, are there any explanations for how this happens and what is the molecular mechanism that triggers their firing?  Furthermore, what makes CIS particles cluster together (Fig. 3d-f)? Does that matter or is just a coincidence?  Finally, why would CISSc mutants sporulate earlier compared to WT?  It would be interesting to see more details on these aspects, even if just speculation on the possible mechanisms.  MINOR COMMENTS  Fig. 4 should be made colorblind friendly. For example, red color should be replaced with magenta. Ext. Data. Fig. 5: same as above Ext. Data. Fig. 6: same as above While not mandatory, I encourage the authors to share their experimental cryo-EM and cryo-ET raw data on the Electron Microscopy Image Archie (EMPIAR: https://www.ebi.ac.uk/empiar/).  TYPOS  Line 612: coverslip Line 644: thickness of Line 857, Fig. 5a legend: …that *was* attached…  [redacted]    Reviewer #4 (Remarks to the Author):  The authors identified and described the structure and function of a novel contractile injection system in Streptomyces. Unlike other CIS, which deliver effectors extracellularly to affect target cells, they showed the CIS from the model organism Streptomyces coelicolor is important for the development of Streptomyces, as the absence of the CIS leads to premature spore formation and low secondary metabolite production. This study is very well executed, the data are well presented and described. The study is novel and very interesting even for a broad audience.  To further support the claims that the authors make, it would be interesting to try to image CIS localization during stress. Would it be possible to see if CIS slow their movement when interacting with the membrane (during the sheath contraction event)? Would this correlate with cell death? Also, could the authors explain why they use loss of cytosolic GFP signal as a marker for cell death? Why not to use for example propidium iodide or some other DNA stain that allows for testing membrane permeability? Or could you measure cell death some other way? My concern is that the observed decrease in GFP signal could be potentially also explained by decreased protein synthesis (or poor GFP folding) rather than cell death/lysis.  Authors speculate that contracting CIS could induce hyphal cell death, which would then impact Streptomyces development. However, the fact that CIS mutants sporulate earlier might also indicate that such mutants might not be able to tolerate moderate stress (like growing in liquid culture for 3 days) as well as wildtype and that such mutants simply enter dormancy (sporulation) prematurely. Could authors comment on this?   Minor issues: Line 26: Fluorescence light microscopy furthermore revealed that CISSc contraction mediates cell death upon encountering different types of stress... As the authors mentioned in the text, the fluorescence imaging show CIS as static foci since the resolution is too low to see sheath contraction. However here it sounds like the authors imaged the contraction event. Maybe say “functional CISsc mediates cell death”?  The authors claim that the tape measure protein is missing in the CIS cluster, but then described both extended and contracted structures to have homogeneous length, which suggests the existence of a tape measure protein. Maybe the sequence identity is too low to identify the corresponding gene? In the FIB milled S. venezuelae the extended structure looks much longer ( > 350 nm), are they also homogeneous in length? Maybe there is a gene in S. venezuelae homologous to S. coelicolor but with an extension of certain repetitive sequence that could be the tape measure protein? Could authors comment on this?  From the Figure 3g, it seems that the CIS structures are observed frequently by cryoET, since each tomogram (corresponding to a small segment of the hyphea ~ 1um) contains ~ 6 CIS structures on average. What percentage of tomograms contain the CIS structures? If the CIS structures indeed appear everywhere (as seen by cryoET), then how do you explain the distribution of YPet foci that are far less frequent (based on the fLM)?  Line 182: ‘partially lysed hyphae’ with a mostly disrupted/vesiculated cytoplasmic membrane (reduced contrast in 2D), indicative of cytoplasmic leakage The “partially lysed hyphae” in Fig3c resembles the apical cross-membrane described in https://www.nature.com/articles/ncomms11836 Fig1f, which is a normal membrane structure that compartmentalize the multinucleoid hyphae. How do you distinguish the partially lysed hyphae from hyphae segment containing cross-membrane? Both of them contain vesiculated cytoplasmic membrane and lack cytosol/DNA.  Line 270: “...observed delay in sporulation ... in CIS mutant” This needs rephrasing as Figure 5b,c shows that the CIS mutants sporulated earlier.  Line 274: Deletion of CIS affects secondary metabolite production. CIS mutants sporulate earlier and the spores are metabolically dormant. Does the CIS mutation affect metabolic rate of the Streptomyces in general or specifically inhibit the production of secondary metabolites?  Line 284: “ … for binding of a potential target cell. This also speaks against a typical T6SS …” It sounds like the T6SS encode a tail fiber like protein, which it does not. Please rephrase.  Line833: Figure 4 a,b. If you do the stress experiment with Cis2-YPet strain, do you see the YPet foci appear where the GFP signal is getting weaker first?  Line892: Extended Figure 3a. It is difficult to see the mutated linker I21 to G25 region is actually continuous from the N terminus. Could you please try to improve visualization of this region?  Supplementary Movie 1: It is 180 degree rotated relative to the figure 2g/h.     *******************  ** Although we cannot publish your paper, it may be appropriate for another journal in the Nature Portfolio. If you wish to explore the journals and transfer your manuscript please use our <a href="https://mts-nmicrobiol.nature.com/cgi-bin/main.plex?el=A5Cg1Hri1A5LkG4X4A9ftdl2CrQXe6LZZVUlshg2MNKAZ">manuscript transfer portal</a>. You will not have to re-supply manuscript metadata and files, but please note that this link can only be used once and remains active until used. For more information, please see our <a href="http://www.nature.com/authors/author_resources/transfer_manuscripts.html?WT.mc_id=EMI_NPG_1511_AUTHORTRANSF&WT.ec_id=AUTHOR">manuscript transfer FAQ</a> page.  Note that any decision to opt in to In Review at the original journal is not sent to the receiving journal on transfer. You can opt in to <i><a href="https://www.nature.com/nature-research/for-authors/in-review">In Review</a></i> at receiving journals that support this service by choosing to modify your manuscript on transfer. In Review is available for primary research manuscript types only. |
| --- | --- |

**Author Rebuttal to Initial comments**

Reviewer Expertise:

Referee #1: Secretion systems; structural biology Referee #2: Cryo electron microscopy

Referee #3: Bacterial structural biology; cryoEM Referee #4: Bacterial injection apparatus

Reviewers Comments:

Reviewer #1 (Remarks to the Author):

The MS “Cytoplasmic contractile injection systems mediate cell death in Streptomyces” describes a very large (huge!) body of work that includes genetic manipulation of Streptomyces coelicolor, several types of cryo-electron microscopy (cryo-electron tomography, cryo-focused ion beam specimen milling, single particle imaging, and negative staining), fluorescence microscopy, correlative cryo-fluorescence and cryo-electron microscopy, mass- spectrometry, and many molecular biology assays. It is a truly impressive MS that is a pleasure to read - a real tour de force! The exact mechanism of action of CISs in Streptomyces remains somewhat unclear, but the MS clearly demonstrates that CISs play a critical role in the development program of Streptomyces. Importantly, even though Streptomyces CISs are most similar to extracellular CISs that mediate cytotoxic activity outside of the producer cell, Streptomyces CISs appear to function inside the cell by a yet unknown mechanism.

I am very impressed with the quality of the data and presentation of the results. I have only a few comments that are aimed at the clarification of some aspects and further improvement of the presentation.

L. 218. will lead -> leads

L. 277 Conclusions -> Discussion

L. 742. Data availability.

In principle, reviewers are expected to analyze validation reports, etc., so the data should have been deposited to databases prior to the submission of the paper. 🡺 This will be addressed accordingly.

Fig. 1f, 1g

Please present additional tomograms that demonstrate localization of CISs in the cytoplasm (a Supplementary or Extended View figure, perhaps?). 🡺 This will be addressed accordingly.

Fig. 2c, 2f. Here and elsewhere, use a consistent orientation of the sheath subunit with respect to the sixfold - desirably, show a sideview. Panels b and e appear to show the sheath with the baseplate in the up position. Phage people draw tails with the baseplate down, so the sheath subunit is usually shown with the N- and C- terminal linker arms pointing downwards as well. So, for consistency and for reaching out to a larger audience, it might be prudent to show the sheath in that orientation. The sheath subunit in panels c and f can also be shown in a “sideway” view with the linker arms pointing downward and the tube subunit next to it.

🡺 This will be addressed accordingly.

Fig. 4:

c-f panels. Do the three different colors indicate three biological repeats?

🡺 Yes, and we will add this to the legend.

e panel: the CCCP + DMSO experiment is not described in the text. 🡺 This will be addressed accordingly.

Extended Data Fig. 1c No MW marker and no major bands identified. So, I am not sure what we are looking at (and looking for) in this figure. 🡺 It is a loading control. We will clarify this.

ED Fig. 2c. The second line label in the sequence alignment should be 1-99, not 1-95.

🡺 This will be addressed accordingly.

ED Fig 3a. Does this panel show the contracted sheath subunit as stated in the legend?

A comparison of the atomic models of the contracted and non-contractile sheath subunit is needed. Why is this mutant non-contractile? Is the topology of the the N-terminal linker arms altered in a similar way to the non- contractile T6SS sheath mutant? This needs to be explained in the text. 🡺 This will be addressed accordingly.

ED Fig 4a. As the sheath subunit is not in a “standard” orientation, the location of the insertion site with respect to the overall structure of the sheath is difficult to understand. The disordered domain should also be indicated.

🡺 This will be addressed accordingly.

ED Fig 7. The scale of the first and third columns must be different despite the same size scale bar. If the scale is indeed the same, an explanation of why the structures have different apparent sizes is needed.

🡺 This will be addressed accordingly. Reviewer #2 (Remarks to the Author):

The manuscript by Casu and colleagues reveals that the contractile injection system (CIS) in a bacterium Streptomyces modulates cell death. For this the authors identified the components of the system using sequence analysis tools, persormed structural analysis of purified contracted sheath and extended theath plus the inner tubeby single particle cryo-EM. The authors further performed cellular imaging by electron cryotomography and showed that the injections systems are not attached to the cell walls and that the fraction of contracted systems was higher in partially lysed and ghost cells. Finally the authors defined a new cellular role for the CIS in S. coelicolor - it is related to stress-related cell death and is involved in regulation of sporulation as shown by analysing the mutants with CIS knocked out or with a non-functional CIS. In conclusion the authors emphasised that the role of CIS in Streptomyces is different to the other known CISs.

The experiments are well designed and executed, the paper is well structured and written. I have several important comments:

1. The authors were succesful in obtaining high resolution structural information about the CIS by single particle cryo-EM, however all they say is that is is the same as the other systems and all they use it for is to design a non/contractile mutant and add a fluorophore. However this was probably possible to do even without knowing the structure, by just looking at the sequence of an Alphafold2 predictions? Is there anything different to the model CISs?

🡺 It was not possible to design the mutant based on Alphafold2. The high-resolution structure was essential. We will clarify this point and add a comparison to other systems.

2. I am curious if this CIS is supposed to be recycled by a mechanism similar to ClpV in T6SS? Does the genome contain a ClpV homologue?

🡺 We could not detect a ClpV homologue in the genome. We will add this information to the text.

3. However, most importantly, the authors claim to show the new functional role of CIS in Streptomyces, but the mechanism of action is unclear which makes the message rather descriptive. The authors suggest that there may be membrane- or cell wall-targeting effectors involved. If true this would have been very exciting, could the athors identify/suggest/conditionally express some of those putative effectors and show the same mechanism as the CIS? This would make the paper much more interesting.

🡺 Since submission, we did in fact identify potential effectors in the CIS gene cluster. Interestingly, when over- expressed in *E. coli*, these effectors are toxic. We will further investigate these effectors and include the data in the revised version of the manuscript.

Reviewer #3 (Remarks to the Author):

REVIEW OF NMICROBIOL-22071688 SUMMARY

In this study, Casu et al. present a comprehensive structural and functional analysis of the Streptomyces contractile injection system (CIS). This work builds upon recent findings that CISs, syringe-like protein complexes that can secrete effector cargo proteins, are much more widespread and performing different functions than previously thought. For example, type VI secretion systems (T6SSs) are anchored to the inner membrane of gram-negative bacteria and are specialized in attacking other cells. Recently reported thylakoid CISs from cyanobacteria target the cell’s own outer membrane in a type of programmed cell death. Or, what would seemingly be the mode of action here, conventional extracellular CISs (eCISs) are assembled as free-floating particles in the cytoplasm for later release into the medium, then targeting other cells. Surprisingly though, the authors here first show through bioinformatics analyses that the S. coelicolor and S. venezuelae CIS gene clusters both lack a

tail fiber protein (Afp13) and a tail measure protein (Afp14), hinting that this CIS does not target other cells. Furthermore, experimental evidence is given that the Streptomyces CIS is assembled and confined to the cytoplasm, in stark contrast to other known CISs.

The authors use single particle cryo-electron microscopy (cryo-EM) to resolve the structure of the contracted S. coelicolor CIS (CISSc) at 3.6 Å resolution, the first from a gram-positive bacterium to be reported. This structure is then used to inform the engineering of a non-functional extended CISSc as well as a fluorescently tagged variant, allowing them to perform time-resolved subcellular localization by fluorescence light microscopy (fLM) as well as correlated light and electron microscopy (CLEM). Notably, the authors also did cryo-electron tomography (cryo-ET) of native cells, which showed that the level of contraction of the CISSc correlates with cellular integrity, as they reveal that clusters CISSc tend to be found closer to the membrane of damaged cells. Especially in “ghost” cells where essentially only the peptidoglycan wall is left, clusters of contracted CISSc can be observed.

In possession of the mutants and imaging tools, the study goes on to elegantly investigate the function of the Streptomyces CIS under various cellular stress conditions (+Nisin, +CCCP, +UV) as well as in the multicellular development of Streptomyces colonies and their production of secondary metabolites.

As a non-expert in the field of CISs, nor in Streptomyces, I found the study to be concisely presented and easy to follow. Complementary imaging techniques are well employed to shed light on the unusual function of a little studied protein complex in a model organism of high importance to agriculture and antibiotics production. I fully recommend the publication of this study only with minor corrections.

MAJOR COMMENTS

Given the central importance of the cryo-EM structure in the study, the experimental densities with fitted models should be shown in a main figure. Currently they are only shown zoomed out in the local resolution plots in Extended Data Figs. 2 and 3, making it difficult to assess the quality of the map and the respective model, considering that this resolution range is particularly tricky to model. For example, a figure similar to Ext. Data. Fig. 3a with the EM density instead of a surface model could be shown, for the WT and the engineered non- contractile mutant.

🡺 This will be addressed accordingly.

Furthermore, no “Table 1” information was provided with details on the map and model statistics. 🡺 This will be addressed accordingly.

On the biological aspects of the work, my comments are curiosities of a non-expert that do not hinder publication of the study, and authors should address them at their discretion:

While it is conceivable, in light of the evidence presented, that CISSc are recruited to the membrane, are there any explanations for how this happens and what is the molecular mechanism that triggers their firing?

🡺 At this point we are unable to provide additional experimental evidence for the mechanism of action. That said, we did detect a putative tail fiber-like gene in the vicinity of the gene cluster. We will include this additional information in the manuscript and discuss its implications.

Furthermore, what makes CIS particles cluster together (Fig. 3d-f)? Does that matter or is just a coincidence?

🡺 We will discuss this observation.

Finally, why would CISSc mutants sporulate earlier compared to WT?

🡺 It is possible and has been argued previously that suicide is an integral part of the *Streptomyces* development (https://doi.org/10.1038/nrmicro3178). Sporulation is a response to nutrient depletion. It is conceivable that developmentally induced cell death of part of the mycelium results in the release of nutrients that prolong the vegetative growth phase in the wild type compared to CISs mutants.

It would be interesting to see more details on these aspects, even if just speculation on the possible mechanisms. MINOR COMMENTS

🡺 Minor comments will be addressed accordingly.

Fig. 4 should be made colorblind friendly. For example, red color should be replaced with magenta. Ext. Data. Fig. 5: same as above

Ext. Data. Fig. 6: same as above

While not mandatory, I encourage the authors to share their experimental cryo-EM and cryo-ET raw data on the Electron Microscopy Image Archie (EMPIAR: https://[www.ebi.ac.uk/empiar/).](http://www.ebi.ac.uk/empiar/))

TYPOS

Line 612: coverslip Line 644: thickness of

Line 857, Fig. 5a legend: …that *was* attached…

[redacted]

Reviewer #4 (Remarks to the Author):

The authors identified and described the structure and function of a novel contractile injection system in Streptomyces. Unlike other CIS, which deliver effectors extracellularly to affect target cells, they showed the CIS from the model organism Streptomyces coelicolor is important for the development of Streptomyces, as the absence of the CIS leads to premature spore formation and low secondary metabolite production. This study is very well executed, the data are well presented and described. The study is novel and very interesting even for a broad audience.

To further support the claims that the authors make, it would be interesting to try to image CIS localization during stress. Would it be possible to see if CIS slow their movement when interacting with the membrane (during the sheath contraction event)? Would this correlate with cell death?

🡺 We show that fluorescently tagged CIS particles are largely static during unperturbed growth (Figure 2g and h and Supplementary Movie 1). We have attempted to visualise the CIS localisation dynamics during treatment with nisin but were unable to resolve any effects on their movement or interaction with the membrane by light microscopy (data not shown).

Also, could the authors explain why they use loss of cytosolic GFP signal as a marker for cell death? Why not to use for example propidium iodide or some other DNA stain that allows for testing membrane permeability? Or could you measure cell death some other way? My concern is that the observed decrease in GFP signal could be potentially also explained by decreased protein synthesis (or poor GFP folding) rather than cell death/lysis.

🡺 We initially used propidium iodide and found that it resulted in heterogeneous and non-reproducible staining. A codon-optimized and cytoplasmic sfGFP produced from a constitutive promoter was our next best choice. The control experiments, including untreated cells and different mutant backgrounds, demonstrates that the reduction in sfGFP fluorescence clearly correlates with the presence of functional CIS and signs of cell lysis/death (Figure 4 and Extended Data Figure 5 and 6).

Authors speculate that contracting CIS could induce hyphal cell death, which would then impact Streptomyces development. However, the fact that CIS mutants sporulate earlier might also indicate that such mutants might not be able to tolerate moderate stress (like growing in liquid culture for 3 days) as well as wildtype and that such mutants simply enter dormancy (sporulation) prematurely. Could authors comment on this?

🡺 We note that *Streptomyces coelicolor* only sporulates on solid medium and we therefore cannot experimentally test this idea. As discussed above, a potential explanation for the earlier sporulation phenotype in the CIS mutant is that CIS-mediated hyphal cell death results in the release of nutrients that in the case of the wild-type, result in a later onset of sporulation on solid medium.

Minor issues:

Line 26: Fluorescence light microscopy furthermore revealed that CISSc contraction mediates cell death upon encountering different types of stress...

As the authors mentioned in the text, the fluorescence imaging show CIS as static foci since the resolution is too low to see sheath contraction. However here it sounds like the authors imaged the contraction event. Maybe say “functional CISsc mediates cell death”?

🡺 We will revise and clarify this statement.

The authors claim that the tape measure protein is missing in the CIS cluster, but then described both extended and contracted structures to have homogeneous length, which suggests the existence of a tape measure protein. Maybe the sequence identity is too low to identify the corresponding gene? In the FIB milled S. venezuelae the extended structure looks much longer ( > 350 nm), are they also homogeneous in length? Maybe there is a gene in S. venezuelae homologous to S. coelicolor but with an extension of certain repetitive sequence that could be the tape measure protein? Could authors comment on this?

🡺 We did not detect a putative tape measure protein based on sequence identity and we agree that this does not formally exclude the presence of the corresponding gene. We will soften the language surrounding the statement about the absence of a tape measure protein from the CIS cluster.

Based on our *in situ* imaging of CIS particles, CISs from *S. coelicolor* and *S. venezuelae* are similar in length

From the Figure 3g, it seems that the CIS structures are observed frequently by cryoET, since each tomogram (corresponding to a small segment of the hyphea ~ 1um) contains ~ 6 CIS structures on average. What percentage of tomograms contain the CIS structures? If the CIS structures indeed appear everywhere (as seen by cryoET), then how do you explain the distribution of YPet foci that are far less frequent (based on the fLM)?

🡺 ~50% of tomograms contain CIS structures. We speculate that the somewhat lower frequency of CISs observed by fLM is based on the fact that the label on contracted CISs inside a dying hypha may be degraded over time.

Line 182: ‘partially lysed hyphae’ with a mostly disrupted/vesiculated cytoplasmic membrane (reduced contrast in 2D),	indicative	of	cytoplasmic	leakage The “partially lysed hyphae” in Fig3c resembles the apical cross-membrane described in https://[www.nature.com/articles/ncomms11836](http://www.nature.com/articles/ncomms11836) Fig1f, which is a normal membrane structure that compartmentalize the multinucleoid hyphae. How do you distinguish the partially lysed hyphae from hyphae segment containing cross-membrane? Both of them contain vesiculated cytoplasmic membrane and lack cytosol/DNA.

🡺 The observation of cross-membranes in live hyphae is somewhat controversially discussed in the field. There is also experimental evidence that suggests that the presence of cross-membranes is associated with cell death (https://[www.nature.com/articles/s41467-017-01596-z). In addition, the cross-membranes described in the paper](http://www.nature.com/articles/s41467-017-01596-z).Inaddition%2Cthecross-membranesdescribedinthepaper) highlighted by the reviewer are visible in a defined subsection of hyphae while the membranous structures we observed in partially lysed hyphae are much more extensive. We will provide additional images to make the extent of membrane damage clearer in the revised manuscript.

Line 270: “...observed delay in sporulation ... in CIS mutant”

This needs rephrasing as Figure 5b,c shows that the CIS mutants sporulated earlier.

🡺 This will be addressed accordingly.

Line 274: Deletion of CIS affects secondary metabolite production.

CIS mutants sporulate earlier and the spores are metabolically dormant. Does the CIS mutation affect metabolic rate of the Streptomyces in general or specifically inhibit the production of secondary metabolites?

🡺 We have performed metabolomic analyses comparing the wildtype and the CIS mutant grown in different liquid media but did not observe any markedly effects on the metabolic rate between the different strains. The production of many secondary metabolites (e.g., actinorhodin) is genetically and temporarily tightly coordinated with cellular development, and thus their production is likely to be affected by the altered timing of sporulation in the CIS mutant.

Line 284: “ … for binding of a potential target cell. This also speaks against a typical T6SS …” It sounds like the T6SS encode a tail fiber like protein, which it does not. Please rephrase.

🡺 This will be addressed accordingly.

Line833: Figure 4 a,b.

If you do the stress experiment with Cis2-YPet strain, do you see the YPet foci appear where the GFP signal is getting weaker first?

🡺 We have not imaged these two markers together due to the overlap in their fluorescent spectra which we cannot resolve with our microscope. We have performed a similar experiment in which we monitored the distribution and the dynamics of Cis2-YPet foci following the transfer of *Streptomyces* hyphae onto agarose pads containing nisin. We did not observe the appearance of additional Cis2-YPet foci under these experimental conditions.

Line892: Extended Figure 3a.

It is difficult to see the mutated linker I21 to G25 region is actually continuous from the N terminus. Could you please try to improve visualization of this region?

🡺 This will be addressed accordingly.

Supplementary Movie 1:

It is 180 degree rotated relative to the figure 2g/h.

🡺 This will be addressed accordingly.

**Decision Letter, first revision:**

| **Message:** | 3rd October 2022  Dear Martin,  Thank you for your letter asking us to reconsider our decision on your Article entitled "Cytoplasmic contractile injection systems mediate cell death in Streptomyces". After careful consideration we have decided that we would be willing to consider a revised version of your manuscript.  Please add the data on potential effectors and the additional information on the putative tail fiber-like gene to the manuscript. Please also address all other referee comments. However, editorially we will not require you to add additional data on the MOA.  Along with your revised manuscript, you should also submit a separate point-by-point response to all of the concerns raised by the referees, in each case describing what changes have been made to the manuscript or, alternatively, if no action has been taken, providing a compelling argument for why that is the case. If we feel that a substantial attempt has been made to address the referees' comments, this response will be sent back to the referees - along with the revised manuscript - so that they can judge whether their concerns have been addressed satisfactorily or otherwise.  I should stress, however, that we would be reluctant to engage our referees again unless we thought that their comments had been addressed in full.  When revising your paper:  - ensure it complies with our format requirements for Letters as set out in our guide to authors at www.nature.com/nmicrobiol/authors/index.html  - state in a cover note the length of the text, methods and legends; the number of references and the number of display items.  Please ensure that all correspondence is marked with your Nature Microbiology reference number in the subject line.  [redacted]  We hope to receive your revised paper within four weeks. If you cannot send it within this time, please let us know so that we can close your file. In this event, we will still be happy to reconsider your paper at a later date so long as nothing similar has been accepted for publication at Nature Microbiology or published elsewhere in the meantime. Should you miss the four-week deadline and your paper is eventually published, the received date will be that of the revised, not the original, version.  I would appreciate it if you could tell me if you think you will be able to submit a revised manuscript, and also the likely timescale.  I look forward to hearing from you soon.  [redacted] |
| --- | --- |
|  |  |
|  |  |

**Author Rebuttal, first revision:**

Reviewer Expertise:

Referee #1: Secretion systems; structural biology Referee #2: Cryo electron microscopy

Referee #3: Bacterial structural biology; cryoEM Referee #4: Bacterial injection apparatus

Reviewers Comments:

Reviewer #1 (Remarks to the Author):

The MS “Cytoplasmic contractile injection systems mediate cell death in Streptomyces” describes a very large (huge!) body of work that includes genetic manipulation of Streptomyces coelicolor, several types of cryo-electron microscopy (cryo-electron tomography, cryo-focused ion beam specimen milling, single particle imaging, and negative staining), fluorescence microscopy, correlative cryo-fluorescence and cryo-electron microscopy, mass- spectrometry, and many molecular biology assays. It is a truly impressive MS that is a pleasure to read - a real tour de force! The exact mechanism of action of CISs in Streptomyces remains somewhat unclear, but the MS clearly demonstrates that CISs play a critical role in the development program of Streptomyces. Importantly, even though Streptomyces CISs are most similar to extracellular CISs that mediate cytotoxic activity outside of the producer cell, Streptomyces CISs appear to function inside the cell by a yet unknown mechanism.

I am very impressed with the quality of the data and presentation of the results.

🡺 Thank you for your encouraging comments.

I have only a few comments that are aimed at the clarification of some aspects and further improvement of the presentation.

L. 218. will lead -> leads

🡺 Revised as suggested.

L. 277 Conclusions -> Discussion

🡺 Revised as suggested.

L. 742. Data availability.

In principle, reviewers are expected to analyze validation reports, etc., so the data should have been deposited to databases prior to the submission of the paper.

🡺 Representative tomograms (EMD-16200, EMD-16201, EMD-16202, EMD-16203, EMD-16204, EMD-16205,

EMD-16206, EMD-16208 and EMD-16210) and SPA cryoEM maps (EMD-16098 and EMD-16101) have been deposited in the Electron Microscopy Data Bank. Atomic models (PDB: 8BKY and PDB: 8BL4) have been deposited in the Protein Data Bank.

Fig. 1f, 1g

Please present additional tomograms that demonstrate localization of CISs in the cytoplasm (a Supplementay or Extended View figure, perhaps?).

🡺 We added additional tomograms in Extended Data Figure 1 d/e.

Fig. 2c, 2f. Here and elsewhere, use a consistent orientation of the sheath subunit with respect to the sixfold - desirably, show a sideview. Panels b and e appear to show the sheath with the baseplate in the up position. Phage people draw tails with the baseplate down, so the sheath subunit is usually shown with the N- and C- terminal linker arms pointing downwards as well. So, for consistency and for reaching out to a larger audience, it might be prudent to show the sheath in that orientation. The sheath subunit in panels c and f can also be shown in a “sideway” view with the linker arms pointing downward and the tube subunit next to it.

🡺 Thank you for the suggestion. We now indicate the orientation of the structures shown in Fig. 2. Since for non- phage CISs (in particular the T6SS) it is common that the apparatus is shown in the opposite direction, we chose to keep the “baseplate up” orientation.

Fig. 4:

c-f panels. Do the three different colors indicate three biological repeats?

🡺 Yes, and we added this information to the figure legend.

e panel: the CCCP + DMSO experiment is not described in the text.

🡺 We revised the text accordingly (lines 228-230).

Extended Data Fig. 1c No MW marker and no major bands identified. So, I am not sure what we are looking at (and looking for) in this figure.

🡺 Extended Data Fig. 1c shows a Coomassie-stained SDS PAGE gel. The panel serves as a loading control for the automated Western blot experiment presented in Fig. 1e. We clarified this in the legend of Extended Data Figure 1c.

ED Fig. 2c. The second line label in the sequence alignment should be 1-99, not 1-95.

🡺 Revised as suggested.

ED Fig 3a. Does this panel show the contracted sheath subunit as stated in the legend?

🡺 Yes.

A comparison of the atomic models of the contracted and non-contractile sheath subunit is needed.

🡺 We added this information as ED Fig. 3h/i. Why is this mutant non-contractile?

🡺The insertion of the five amino acids IEGVG extends the N-terminal sheath linker and thereby abrogates the propagation of contraction. This approach has previously successfully been used in *V. cholerae* (https://[www.nature.com/articles/s41564-017-0020-7).](http://www.nature.com/articles/s41564-017-0020-7))

Is the topology of the the N-terminal linker arms altered in a similar way to the non-contractile T6SS sheath mutant? This needs to be explained in the text.

🡺 Yes, it is. We addressed this in the text (lines 158-160) and we added two new panels to the ED Figure 3h/i to illustrate the topology of the linker arms better.

ED Fig 4a. As the sheath subunit is not in a “standard” orientation, the location of the insertion site with respect to the overall structure of the sheath is difficult to understand. The disordered domain should also be indicated.

🡺 We added the position of the disordered domain 3 in all figures.

ED Fig 7. The scale of the first and third columns must be different despite the same size scale bar. If the scale is indeed the same, an explanation of why the structures have different apparent sizes is needed.

🡺 Revised as suggested. We apologize that the definition of the scale bar was incorrect.

Reviewer #2 (Remarks to the Author):

The manuscript by Casu and colleagues reveals that the contractile injection system (CIS) in a bacterium Streptomyces modulates cell death. For this the authors identified the components of the system using sequence analysis tools, persormed structural analysis of purified contracted sheath and extended theath plus the inner tubeby single particle cryo-EM. The authors further performed cellular imaging by electron cryotomography and showed that the injections systems are not attached to the cell walls and that the fraction of contracted systems was higher in partially lysed and ghost cells. Finally the authors defined a new cellular role for the CIS in S. coelicolor - it is related to stress-related cell death and is involved in regulation of sporulation as shown by analysing the mutants with CIS knocked out or with a non-functional CIS. In conclusion the authors emphasised that the role of CIS in Streptomyces is different to the other known CISs.

The experiments are well designed and executed, the paper is well structured and written. I have several important comments:

1. The authors were succesful in obtaining high resolution structural information about the CIS by single particle cryo-EM, however all they say is that is is the same as the other systems and all they use it for is to design a non/contractile mutant and add a fluorophore. However this was probably possible to do even without knowing the structure, by just looking at the sequence of an Alphafold2 predictions? Is there anything different to the model CISs?

🡺 As stated in the text, there are no significant differences to other model CIS. Nevertheless, it was impossible to design the mutant based on comparisons to other systems, Alphafold2 predictions of monomers, or sequence analyses. The exact amino acid position where the linker had to be inserted could only be determined by the high- resolution structure of the assembled contracted sheath.

2. I am curious if this CIS is supposed to be recycled by a mechanism similar to ClpV in T6SS? Does the genome contain a ClpV homologue?

🡺 We did not detect a ClpV homologue in the CIS gene cluster of *S. coelicolor* and *S. venezuelea*. We added this information to the manuscript (lines 96-97).

3. However, most importantly, the authors claim to show the new functional role of CIS in Streptomyces, but the mechanism of action is unclear which makes the message rather descriptive. The authors suggest that there may be membrane- or cell wall-targeting effectors involved. If true this would have been very exciting, could the athors identify/suggest/conditionally express some of those putative effectors and show the same mechanism as the CIS? This would make the paper much more interesting.

🡺 In order to address the reviewer’s comment, we have performed additional experiments and added the results to the manuscript as Fig. 6, ED Fig. 10, and Supplementary Table 4.

We have indeed identified three potential effectors (Sco4256-4258) in the CIS gene cluster (Fig. 6a) that appear to be specifically conserved in *Streptomyces* species that encode CIS (Supplementary Table 4). As suggested, we first expressed the three putative genes encoding the putative effectors in *E. coli* and found that the expression of these genes was toxic to *E. coli* (Fig. 6b). Furthermore, we generated a *S. coelicolor* mutant that lacked all three effector genes and showed that this mutant produced CISSc particles that lacked an electron density within the inner tubes (Fig. 6c-e). In addition, we demonstrate that the effector deletion mutant phenocopies other CISSc mutants in our functional assays, including cell viability upon stress, timing/amount of sporulation, and secondary metabolite production (Fig. 6f-i, ED Fig. 10). We therefore conclude that Sco4256-4258 are CISSc-associated effectors that are loaded into the CIS and released upon firing to mediate cell death. These new results are now reported in lines 278-305.

Reviewer #3 (Remarks to the Author):

REVIEW OF NMICROBIOL-22071688 SUMMARY

In this study, Casu et al. present a comprehensive structural and functional analysis of the Streptomyces contractile injection system (CIS). This work builds upon recent findings that CISs, syringe-like protein complexes that can secrete effector cargo proteins, are much more widespread and performing different functions than previously thought. For example, type VI secretion systems (T6SSs) are anchored to the inner membrane of gram-negative bacteria and are specialized in attacking other cells. Recently reported thylakoid CISs from cyanobacteria target the cell’s own outer membrane in a type of programmed cell death. Or, what would seemingly be the mode of action here, conventional extracellular CISs (eCISs) are assembled as free-floating particles in the cytoplasm for later release into the medium, then targeting other cells. Surprisingly though, the

authors here first show through bioinformatics analyses that the S. coelicolor and S. venezuelae CIS gene clusters both lack a tail fiber protein (Afp13) and a tail measure protein (Afp14), hinting that this CIS does not target other cells. Furthermore, experimental evidence is given that the Streptomyces CIS is assembled and confined to the cytoplasm, in stark contrast to other known CISs.

The authors use single particle cryo-electron microscopy (cryo-EM) to resolve the structure of the contracted S. coelicolor CIS (CISSc) at 3.6 Å resolution, the first from a gram-positive bacterium to be reported. This structure is then used to inform the engineering of a non-functional extended CISSc as well as a fluorescently tagged variant, allowing them to perform time-resolved subcellular localization by fluorescence light microscopy (fLM) as well as correlated light and electron microscopy (CLEM). Notably, the authors also did cryo-electron tomography (cryo-ET) of native cells, which showed that the level of contraction of the CISSc correlates with cellular integrity, as they reveal that clusters CISSc tend to be found closer to the membrane of damaged cells. Especially in “ghost” cells where essentially only the peptidoglycan wall is left, clusters of contracted CISSc can be observed.

In possession of the mutants and imaging tools, the study goes on to elegantly investigate the function of the Streptomyces CIS under various cellular stress conditions (+Nisin, +CCCP, +UV) as well as in the multicellular development of Streptomyces colonies and their production of secondary metabolites.

As a non-expert in the field of CISs, nor in Streptomyces, I found the study to be concisely presented and easy to follow. Complementary imaging techniques are well employed to shed light on the unusual function of a little studied protein complex in a model organism of high importance to agriculture and antibiotics production. I fully recommend the publication of this study only with minor corrections.

🡺 Thank you for these encouraging comments. MAJOR COMMENTS

Given the central importance of the cryo-EM structure in the study, the experimental densities with fitted models should be shown in a main figure. Currently they are only shown zoomed out in the local resolution plots in Extended Data Figs. 2 and 3, making it difficult to assess the quality of the map and the respective model, considering that this resolution range is particularly tricky to model. For example, a figure similar to Ext. Data. Fig. 3a with the EM density instead of a surface model could be shown, for the WT and the engineered non- contractile mutant.

🡺 We revised Fig. 2c/g according to the reviewer’s suggestion.

Furthermore, no “Table 1” information was provided with details on the map and model statistics.

🡺 This data has now been added to Supplementary Table 2.

On the biological aspects of the work, my comments are curiosities of a non-expert that do not hinder publication of the study, and authors should address them at their discretion:

While it is conceivable, in light of the evidence presented, that CISSc are recruited to the membrane, are there any explanations for how this happens and what is the molecular mechanism that triggers their firing?

🡺 At this point we are unable to provide additional experimental evidence for the mechanism of action. That said, we did detect a putative membrane adaptor (*sco4242*) adjacent to the CIS gene cluster. This gene was found just downstream of putative baseplate components. In other systems, this region often harbors tail fiber-like genes. We also found that homologues of *sco4242* can be detected in many other CIS-positive *Streptomyces* strains, however, they are absent from CIS-negative strains. This conservation together with the presence of a predicted trans- membrane domain in Sco4242 suggests a functional relationship to CISSc and a potential role as an adaptor which could mediate the interaction of cytoplasmic CISSc particles with the cytoplasmic membrane to deliver the effector proteins.

We now included this additional information in lines 322-335 and in Supplementary Table 4.

Furthermore, what makes CIS particles cluster together (Fig. 3d-f)? Does that matter or is just a coincidence?

🡺 The clustering of contracted sheath structures is a phenomenon that can be observed across diverse systems, including also the classical T6SS-i. The mechanism for this clustering is not understood. Clustering of extended structures has been reported for the T6SS-iv in *Ca. Amoebophilus asiaticus* (https://pubmed.ncbi.nlm.nih.gov/28818949/), where the interactions are mediated by lateral baseplate interactions. The extended CISSc observed here were mostly seen as individual structures. Occasional clustering does likely not represent a specific mechanism.

Finally, why would CISSc mutants sporulate earlier compared to WT?

It would be interesting to see more details on these aspects, even if just speculation on the possible mechanisms.

🡺 It is possible and has been argued previously that suicide is an integral part of *Streptomyces* development (https://doi.org/10.1038/nrmicro3178). Sporulation is a response to nutrient depletion. It is conceivable that developmentally induced cell death of part of the mycelium results in the release of nutrients that prolong the vegetative growth phase in the wild type compared to CISSc mutants. We now discuss this in lines 336-345.

MINOR COMMENTS

Fig. 4 should be made colorblind friendly. For example, red color should be replaced with magenta. Ext. Data. Fig. 5: same as above

Ext. Data. Fig. 6: same as above

🡺 We will change colors in case this is required upon editorial assessment by the journal.

While not mandatory, I encourage the authors to share their experimental cryo-EM and cryo-ET raw data on the Electron Microscopy Image Archie (EMPIAR: https://[www.ebi.ac.uk/empiar/).](http://www.ebi.ac.uk/empiar/))

🡺 EM data has been deposited at EMDB and PDB. We will consider submission to EMPIAR upon acceptance.

TYPOS

🡺 Thanks for pointing out typos – they have all been corrected.

Line 612: coverslip Line 644: thickness of

Line 857, Fig. 5a legend: …that *was* attached…

[redacted]

Reviewer #4 (Remarks to the Author):

The authors identified and described the structure and function of a novel contractile injection system in Streptomyces. Unlike other CIS, which deliver effectors extracellularly to affect target cells, they showed the CIS from the model organism Streptomyces coelicolor is important for the development of Streptomyces, as the absence of the CIS leads to premature spore formation and low secondary metabolite production. This study is very well executed, the data are well presented and described. The study is novel and very interesting even for a broad audience.

🡺 Thank you for your encouraging comments.

To further support the claims that the authors make, it would be interesting to try to image CIS localization during stress. Would it be possible to see if CIS slow their movement when interacting with the membrane (during the sheath contraction event)? Would this correlate with cell death?

🡺 We show that fluorescently tagged CISSc particles are largely static during unperturbed growth (Figure 2i/j and Supplementary Movie 1). We have attempted to visualize CISSc localization and dynamics during treatment with nisin but we were unfortunately unable to resolve any effects on their movement or interaction with the membrane by light microscopy (data not shown). This is possibly due to limitations in resolution.

Also, could the authors explain why they use loss of cytosolic GFP signal as a marker for cell death? Why not to use for example propidium iodide or some other DNA stain that allows for testing membrane permeability? Or could you measure cell death some other way? My concern is that the observed decrease in GFP signal could be potentially also explained by decreased protein synthesis (or poor GFP folding) rather than cell death/lysis.

🡺 In our initial experiments, we used propidium iodide (and other stains) and found that it resulted in heterogeneous and non-reproducible staining. A codon-optimized and cytoplasmic sfGFP produced from a constitutive promoter was our next best choice. In order to address the point raised by the reviewer, we had included ED Fig. 5, where we used a correlative cryo-fLM cryoEM approach, which allowed us to assess the integrity of cells with a “dead stain” i.e. presence of FM5-95 signal and absence of sfGFP signal. Such cells had a “ghost”-like appearance indicating cell death. Furthermore, our other control experiments (including untreated cells and different mutant backgrounds) demonstrated that the reduction in sfGFP fluorescence clearly correlates with the presence of functional CIS (Figure 4 and Extended Data Figure 6). The explanation that a non-functional CISSc would lead to a decrease in general protein synthesis or misfolding seems rather unlikely.

Authors speculate that contracting CIS could induce hyphal cell death, which would then impact Streptomyces development. However, the fact that CIS mutants sporulate earlier might also indicate that such mutants might not be able to tolerate moderate stress (like growing in liquid culture for 3 days) as well as wildtype and that such mutants simply enter dormancy (sporulation) prematurely. Could authors comment on this?

🡺 We note that *Streptomyces coelicolor* only sporulates on solid medium and we therefore cannot experimentally test this idea. As discussed above, a potential explanation for the earlier sporulation phenotype in the CISSc mutant is that CISSc-mediated hyphal cell death results in the release of nutrients that in the case of the wild-type, result in a later onset of sporulation on solid medium. This is now discussed in lines 340-345.

Minor issues:

Line 26: Fluorescence light microscopy furthermore revealed that CISSc contraction mediates cell death upon encountering different types of stress...

As the authors mentioned in the text, the fluorescence imaging show CIS as static foci since the resolution is too low to see sheath contraction. However here it sounds like the authors imaged the contraction event. Maybe say “functional CISsc mediates cell death”?

🡺 Revised as suggested.

The authors claim that the tape measure protein is missing in the CIS cluster, but then described both extended and contracted structures to have homogeneous length, which suggests the existence of a tape measure protein. Maybe the sequence identity is too low to identify the corresponding gene? In the FIB milled S. venezuelae the extended structure looks much longer ( > 350 nm), are they also homogeneous in length? Maybe there is a gene in S. venezuelae homologous to S. coelicolor but with an extension of certain repetitive sequence that could be the tape measure protein? Could authors comment on this?

🡺 We did not detect a canonical tape measure protein based on sequence identity and we agree that this does not formally exclude the presence of a gene with similar function. We therefore added the word “typical” to the text (line 95). Based on our *in situ* imaging of CISSc particles, CIS from *S. coelicolor* and *S. venezuelae* are similar in length.

From the Figure 3g, it seems that the CIS structures are observed frequently by cryoET, since each tomogram (corresponding to a small segment of the hyphea ~ 1um) contains ~ 6 CIS structures on average. What percentage of tomograms contain the CIS structures? If the CIS structures indeed appear everywhere (as seen by cryoET), then how do you explain the distribution of YPet foci that are far less frequent (based on the fLM)?

🡺 Approximately 50% of our tomograms contained CISSc structures. The comparison of frequencies between fLM and cryoET is somewhat difficult. A difference between both analyses likely results from the fact that contracted CISSc can be observed by cryoET, however, these structures are mainly found in dying/ghost cells, in which the fLM signal (yPET) may have been degraded over time (leading to a lower frequency).

Line 182: ‘partially lysed hyphae’ with a mostly disrupted/vesiculated cytoplasmic membrane (reduced contrast in 2D), indicative of cytoplasmic leakage. The “partially lysed hyphae” in Fig3c resembles the apical cross-membrane described in https://[www.nature.com/articles/ncomms11836](http://www.nature.com/articles/ncomms11836) Fig1f, which is a normal membrane structure that compartmentalize the multinucleoid hyphae. How do you distinguish the partially lysed hyphae from hyphae segment containing cross-membrane? Both of them contain vesiculated cytoplasmic membrane and lack cytosol/DNA.

🡺 The observation of cross-membranes in live hyphae is somewhat controversially discussed in the field. There is also experimental evidence that suggests that the presence of cross-membranes is associated with cell death (https://[www.nature.com/articles/s41467-017-01596-z). In addition, the cross-membranes described in the paper](http://www.nature.com/articles/s41467-017-01596-z).Inaddition%2Cthecross-membranesdescribedinthepaper) highlighted by the reviewer are visible in a defined subsection of hyphae, while the membranous structures we observed in partially lysed hyphae are much more extensive. We provided additional images to make the extent of membrane damage more clear in the revised manuscript in ED Figure 1d/e.

Line 270: “...observed delay in sporulation ... in CIS mutant”

This needs rephrasing as Figure 5b,c shows that the CIS mutants sporulated earlier.

🡺 This was addressed accordingly.

Line 274: Deletion of CIS affects secondary metabolite production.

CIS mutants sporulate earlier and the spores are metabolically dormant. Does the CIS mutation affect metabolic rate of the Streptomyces in general or specifically inhibit the production of secondary metabolites?

🡺 We have performed metabolomic analyses comparing the wild-type and the CISSc-deficient mutant grown in different liquid media but we did not observe any significant effects on the metabolic rate between the different strains. The production of many secondary metabolites (e.g. actinorhodin) is genetically and temporarily tightly coordinated with cellular development, and thus their production is likely to be affected by the altered timing of sporulation in the CIS mutant.

Line 284: “ … for binding of a potential target cell. This also speaks against a typical T6SS …” It sounds like the T6SS encode a tail fiber like protein, which it does not. Please rephrase.

🡺 This was addressed accordingly. Line833: Figure 4 a,b.

If you do the stress experiment with Cis2-YPet strain, do you see the YPet foci appear where the GFP signal is getting weaker first?

🡺 We have not imaged these two markers together due to the overlap in their fluorescent spectra which we cannot resolve with our microscope. We have performed a similar experiment in which we monitored the distribution and the dynamics of Cis2-YPet foci following the transfer of *Streptomyces* hyphae onto agarose pads containing nisin. We did not observe the appearance of additional Cis2-YPet foci under these experimental conditions.

Line892: Extended Figure 3a.

It is difficult to see the mutated linker I21 to G25 region is actually continuous from the N terminus. Could you please try to improve visualization of this region?

🡺 This was addressed accordingly in ED Figure 3a.

Supplementary Movie 1:

It is 180 degree rotated relative to the figure 2g/h.

🡺 We note this rotation in the legend of Supplementary Movie 1.

| **Final Decision Letter:** | | | |
| --- | --- | --- | --- |
|  | **Message:** | Our ref: NMICROBIOL-22071688C  16th January 2023  Dear Martin,  Thank you for your patience as we’ve prepared the guidelines for final submission of your Nature Microbiology manuscript, "Cytoplasmic contractile injection systems mediate cell death in Streptomyces" (NMICROBIOL-22071688C). Please carefully follow the step-by-step instructions provided in the attached file, and add a response in each row of the table to indicate the changes that you have made. Ensuring that each point is addressed will help to ensure that your revised manuscript can be swiftly handed over to our production team.  We would like to start working on your revised paper, with all of the requested files and forms, as soon as possible (preferably within two weeks). Please get in contact with us if you anticipate delays.  When you upload your final materials, please include a point-by-point response to any remaining reviewer comments.  If you have not done so already, please alert us to any related manuscripts from your group that are under consideration or in press at other journals, or are being written up for submission to other journals (see: https://www.nature.com/nature-research/editorial-policies/plagiarism#policy-on-duplicate-publication for details).  In recognition of the time and expertise our reviewers provide to Nature Microbiology’s editorial process, we would like to formally acknowledge their contribution to the external peer review of your manuscript entitled "Cytoplasmic contractile injection systems mediate cell death in Streptomyces". For those reviewers who give their assent, we will be publishing their names alongside the published article.  Nature Microbiology offers a Transparent Peer Review option for new original research manuscripts submitted after December 1st, 2019. As part of this initiative, we encourage our authors to support increased transparency into the peer review process by agreeing to have the reviewer comments, author rebuttal letters, and editorial decision letters published as a Supplementary item. When you submit your final files please clearly state in your cover letter whether or not you would like to participate in this initiative. Please note that failure to state your preference will result in delays in accepting your manuscript for publication.  **Cover suggestions**  As you prepare your final files we encourage you to consider whether you have any images or illustrations that may be appropriate for use on the cover of Nature Microbiology.  Covers should be both aesthetically appealing and scientifically relevant, and should be supplied at the best quality available. Due to the prominence of these images, we do not generally select images featuring faces, children, text, graphs, schematic drawings, or collages on our covers.  We accept TIFF, JPEG, PNG or PSD file formats (a layered PSD file would be ideal), and the image should be at least 300ppi resolution (preferably 600-1200 ppi), in CMYK colour mode.  If your image is selected, we may also use it on the journal website as a banner image, and may need to make artistic alterations to fit our journal style.  Please submit your suggestions, clearly labeled, along with your final files. We’ll be in touch if more information is needed.   Nature Microbiology has now transitioned to a unified Rights Collection system which will allow our Author Services team to quickly and easily collect the rights and permissions required to publish your work. Approximately 10 days after your paper is formally accepted, you will receive an email in providing you with a link to complete the grant of rights. If your paper is eligible for Open Access, our Author Services team will also be in touch regarding any additional information that may be required to arrange payment for your article.  Please note that you will not receive your proofs until the publishing agreement has been received through our system.  Please note that <i>Nature Microbiology</i> is a Transformative Journal (TJ). Authors may publish their research with us through the traditional subscription access route or make their paper immediately open access through payment of an article-processing charge (APC). Authors will not be required to make a final decision about access to their article until it has been accepted. <a href="https://www.springernature.com/gp/open-research/transformative-journals"> Find out more about Transformative Journals</a>  **Authors may need to take specific actions to achieve <a href="https://www.springernature.com/gp/open-research/funding/policy-compliance-faqs"> compliance</a> with funder and institutional open access mandates.** If your research is supported by a funder that requires immediate open access (e.g. according to <a href="https://www.springernature.com/gp/open-research/plan-s-compliance">Plan S principles</a>) then you should select the gold OA route, and we will direct you to the compliant route where possible. For authors selecting the subscription publication route, the journal’s standard licensing terms will need to be accepted, including <a href="https://www.nature.com/nature-portfolio/editorial-policies/self-archiving-and-license-to-publish">self-archiving policies</a>. Those licensing terms will supersede any other terms that the author or any third party may assert apply to any version of the manuscript.   For information regarding our different publishing models please see our <a href="https://www.springernature.com/gp/open-research/transformative-journals"> Transformative Journals </a> page. If you have any questions about costs, Open Access requirements, or our legal forms, please contact ASJournals@springernature.com.  [redacted]  If you have any further questions, please feel free to contact me.   [redacted]   Reviewer #1: Remarks to the Author: The revision of “Cytoplasmic contractile injection systems mediate cell death in Streptomyces” is an improved version of what was already an impressive MS. In the opinion of this scientist, the authors dealt with the concerns raised by the reviewers in the previous version of the MS adequately and fairly.  Despite being much improved, the revised MS contains one minor, but essential, point, which requires clarification. This point escaped the attention of all reviewers and hence was simply copied from the previous version. Namely, the SDS PAGE shown in the Extended Fig. 1c and the associated main text are confusing. There are no molecular weight markers on that gel and no labeled bands. What is the reader expected to see in that gel? A semi-purified sample of an extracellular CIS has a well-defined “signature” in SDS PAGE: tube+sheath+additional-high-copy-number-proteins. But this signature is usually indiscernible in total cell lysates even in CISs that are produced in high copy numbers per cell. Please explain the gel better (label the bands, add MW markers, etc.) or replace with a Western blot (if you have the antibodies). Basically, make the SDS PAGE easier to understand or remove it altogether and adjust the text accordingly.    Reviewer #3: Remarks to the Author: The manuscript "Cytoplasmic contractile injection systems mediate cell death in Streptomyces" by Casu et al has been significantly improved with the addition of new data and clarifications to the text in response to the referees' comments. All my concerns have been adequately addressed and I recommend the immediate publication of the article.  [redacted]    Reviewer #4: Remarks to the Author: I am fully satisfied with the answers and the revised manuscript.    Reviewer #5: Remarks to the Author: This revised manuscript by Casu and colleagues, which I had read via their preprint at bioRxiv but have not reviewed at an earlier stage, identifies contractile injection systems (CIS) in Streptomyces, which are prolific antibiotic producing bacteria with a multicellular lifestyle and complex developmental program. The authors perform a comprehensive bioinformatic approach combined with high-end imaging and genetics to show that these new CIS in Streptomycetes are conserved and involved in self-killing of filaments (also referred to as programmed cell death (PCD) in the community), during the transition from vegetative to aerial growth. Unlike other CIS, these systems operate inside the cell, which is unambiguously demonstrated, amongst others with beautiful cryoET imaging and Western blotting analyses. The data is of high quality and support the main conclusions of this study. In the revised version of the manuscript, the authors have identified 3 putative effector proteins, which, when deleted, phenocopy the CIS mutants described earlier in this study. Furthermore, these effectors inhibit growth of E. coli when produced in excess. While the exact mechanism of CIS related to PCD remains to be elucidated, I find this study intriguing and providing exciting new insights into these CIS. Also, the study provides major advances in the Streptomyces field in helping to understand how cellular stress is coupled to morphological development and programmed cell death.  I congratulate the authors with the beautiful study! |  |
